# BN/BO Doping of *peri*‐Acenoacenes: Modulating Excited States in Trapeziumene Congeners

**DOI:** 10.1002/anie.202517114

**Published:** 2025-09-16

**Authors:** Daniele Poletto, Mauro Marongiu, David Hernández‐Castillo, Rúben R. Ferreira, Martina Crosta, Pradip Kumar Mondal, Leticia González, Davide Bonifazi

**Affiliations:** ^1^ Institute of Organic Chemistry University of Vienna Vienna 1090 Austria; ^2^ Doctoral School in Chemistry (DoSChem) University of Vienna Währinger Straße 42 Vienna 1090 Austria; ^3^ Institute of Theoretical Chemistry Faculty of Chemistry University of Vienna Währinger Straße 17 Vienna 1090 Austria; ^4^ Elettra – Sincrotrone S.S. 14 Km 163.5 in Area Science Park, Basovizza Trieste 34149 Italy

**Keywords:** BN‐doping, Borylation reaction, Molecular graphenoids, Polycyclic aromatic hydrocarbons, Triplet and singlet emitters

## Abstract

The rational design of polycyclic aromatic hydrocarbons that combine chemical and physical robustness with finely tuned optoelectronic properties remains a key challenge in materials science. As an initial step toward this goal, we report the synthesis and comprehensive characterization of a new class of boron‐, nitrogen‐, and oxygen‐doped *peri*‐acenoacenes, termed (2,5,4)‐trapeziumene congeners. Analysis of these systems provides chemical descriptors that could guide the rational tailoring of their properties through peripheral doping. The target trapeziumene congeners were obtained via a sequence of Suzuki–Miyaura and Buchwald–Hartwig couplings, followed by directed borylation, giving both phenylborane and borinic derivatives with diverse peripheral doping sequences. Single‐crystal X‐ray diffraction revealed planar to slightly twisted backbones, with peripheral heteroatomic motifs that modulate π‐conjugation and intermolecular packing. Photophysical studies showed bright fluorescence (*Φ*_F_ up to 0.99), narrow Stokes shifts, and structured phosphorescence at 77 K. Electrochemical analysis demonstrated p‐type behavior and a progressive HOMO–LUMO gap widening upon N→O substitution. Theoretical investigations revealed that N→O substitution asymmetrically affects the excited states, blue‐shifting fluorescence while red‐shifting phosphorescence, through an asymmetric charge stabilization in the S_1_ and T_1_ excited states. This is accompanied by a progressive widening of the T_1_–T_2_ energy gap.

## Graphical abstract

A new family of (2,5,4)‐trapeziumenes bearing NBNBN, NBOBN, OBNBO, and OBOBO doping patterns is synthesized. Progressive N→O substitution enables precise control over the excited‐state landscape, increasing the S_1_ energy, decreasing the T_1_ energy, and widening the T_2_–T_1_ energy gap.

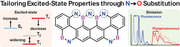

## Introduction

Organic small‐molecule‐based semiconductors have garnered significant attention for their tunable electronic properties, lightweight nature, ease of processing, and potential in flexible electronics. Beyond the challenge of controlling their solid‐state organization,^[^ ^1^ ^]^ which can affect charge transport and device efficiency, their susceptibility to oxidation (including ^1^O_2_), heat, and light exposure remains one of the major obstacles to broader application.^[^ ^2^, ^3^, ^4^, ^5^, ^6^ ^]^ Many high‐performance polycyclic aromatic hydrocarbons (PAHs), especially those with reactive zig‐zag peripheries, are particularly susceptible to degradation, leading to rapid chemical decomposition.^[^ ^7^, ^8^, ^9^, ^10^ ^]^

**Figure 1:**
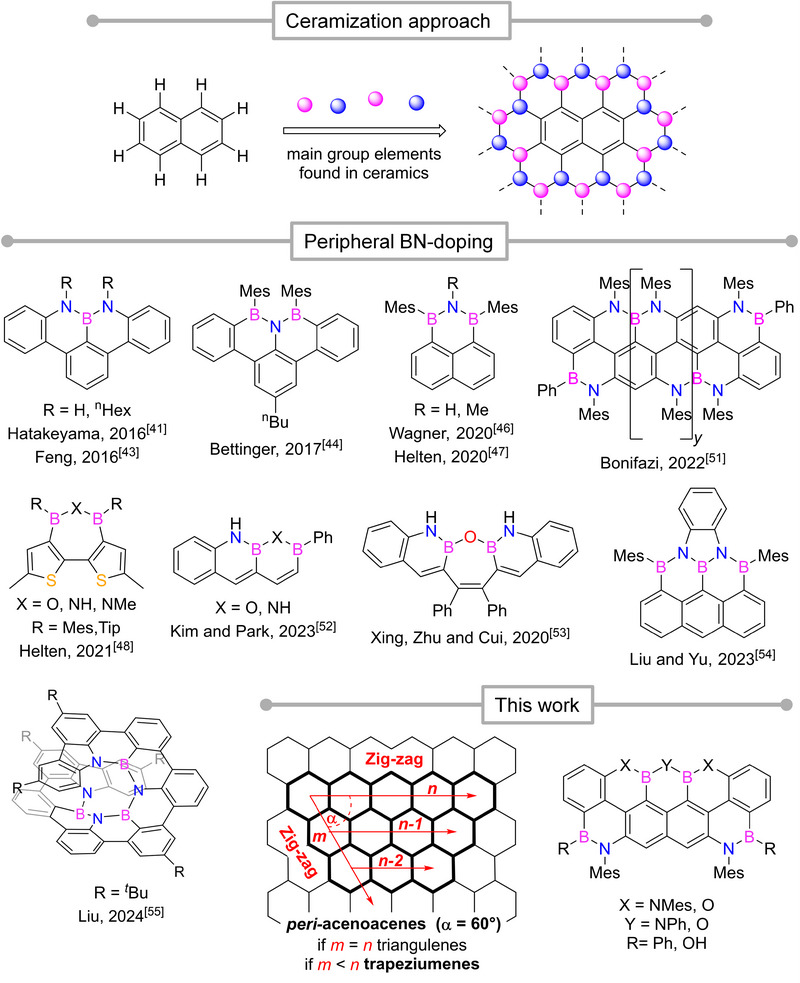
Above: Schematic representation of the molecular ceramization concept, using polycyclic aromatic hydrocarbons as precursors for hybrid organic‐inorganic ceramic materials. Colored spheres represent generic main‐group ceramic‐forming elements (e.g., B, N). Center: examples of previous peripheral BN‐patterns of various polycyclic aromatic hydrocarbons. Below: BN‐doping of trapeziumenes.

Among the diverse strategies to tune the chemical properties of PAHs, boron doping has attracted considerable interest due to its ability to lower LUMO energy levels.^[^ ^11^, ^12^, ^13^, ^14^, ^15^, ^16^ ^]^ A notable example is that of the boron–nitride (BN) doping, in which incorporation of polar B─N bonds, characteristic of ceramic materials such as hexagonal boron nitride (h‐BN), into PAHs yields hybrid organic–inorganic scaffolds with enhanced oxidative and thermal resilience,^[^ ^17^ ^]^ while enabling changing of their optoelectronic properties.^[^ ^18^, ^19^, ^20^, ^21^ ^]^ For instance, core BN‐doping has been investigated as a tool to alter the aromatic and optoelectronic properties of PAHs^[^ ^22^, ^23^, ^24^, ^25^, ^26^, ^27^, ^28^ ^]^ and graphenoids.^[^ ^29^, ^30^, ^31^, ^32^ ^]^ Extension of this concept to the molecular periphery has proven to be an effective strategy, offering greater synthetic flexibility and enabling access to otherwise unstable PAHs.^[^ ^33^, ^34^, ^35^, ^36^, ^37^ ^]^ Seminal examples (Figure 1, center) include the use of triheteroatomic doping units such as NBN or BNB (and their doping congeners bearing O‐atoms^[^ ^38^, ^39^, ^40^ ^]^ instead of N‐atoms), as demonstrated in NBN‐dibenzophenalenes,^[^ ^41^, ^42^ ^]^ NBN‐dibenzoheptazethrene,^[^ ^43^ ^]^ BNB‐benzo[fg]tetracene,^[^ ^44^ ^]^ BNB‐phenalenyls,^[^ ^45^, ^46^, ^47^ ^]^ and dithiophene‐fused oxadiborepins and azadiborepins.^[^ ^48^, ^49^, ^50^ ^]^ Our group has also contributed to this field by synthesizing BN‐doped zig‐zag nanoribbons, incorporating multiple peripheral NBN motifs.^[^ ^51^ ^]^ These systems exhibited p‐type semiconducting behavior alongside thermally activated delayed fluorescence (TADF) properties (Figure 1, center).

A logical next step would be to develop architectures featuring doping motifs that extend beyond the length of a three‐heteroatom skeleton, where ceramized peripheries offer a protective layer around the C(sp^2^)‐based framework while introducing the possibility of fine‐tuning optoelectronic properties (Figure 1, above). However, such an expansion remains a synthetic challenge, with only a few structures reported to date (Figure 1, center). In one example, tetra‐heteroatomic NBNB‐ and NBOB‐doped acene‐type structures were synthesized, displaying remarkable chemical stability and were successfully integrated as emissive materials in OLED‐type devices.^[^ ^52^ ^]^ Expanding to penta‐atomic patterns, bis‐BN‐naphthalene‐fused oxepins,^[^ ^53^ ^]^ and zigzag BNBNB‐embedded anthracene‐fused fluoranthene^[^ ^54^ ^]^ have also been shown to display improved properties. Notably, helicenes have enabled the construction of even longer doping patterns, incorporating up to seven heteroatoms in NBNBN and NBNBNBN sequences at their inner rims, exhibiting strong absorption, luminescence dissymmetry factors, and enhanced CPL brightness.^[^ ^55^ ^]^ A complementary approach employed 1,2,5‐azadiborolane as a modular unit to construct BCN hybrid polymers featuring repeating NBNBN motifs.^[^ ^56^ ^]^ While these examples highlight the feasibility and promise of extended BN‐doping, achieving a balance between chemical robustness and controlled optoelectronic modulation^[^ ^57^ ^]^ remains an open challenge. Addressing this requires rational design strategies capable of systematically controlling the electronic and structural impact of such BN‐doping patterns.

In this work, we tackle this challenge through the synthesis of heteroatom‐decorated zig–zag (XBXBX, X═N or O) peripheries of (2,5,4)‐*peri*‐acenoacenes, also known as (2,5,4)‐trapeziumenes (**1_y_**, with y being the heteroatomic sequence of the doping motif), via a heteroatom‐directed borylation reaction. By systematically substituting nitrogen with oxygen (N→O), a series of extended doping motifs was engineered, including NBNBN, NBOBN, OBNBO, and OBOBO. Their impact on optoelectronic transitions was investigated experimentally and using computational tools to devise a chemical descriptor for rationally tailoring excited‐state properties.

## Results and Discussion

### Synthesis

**Scheme 1:**
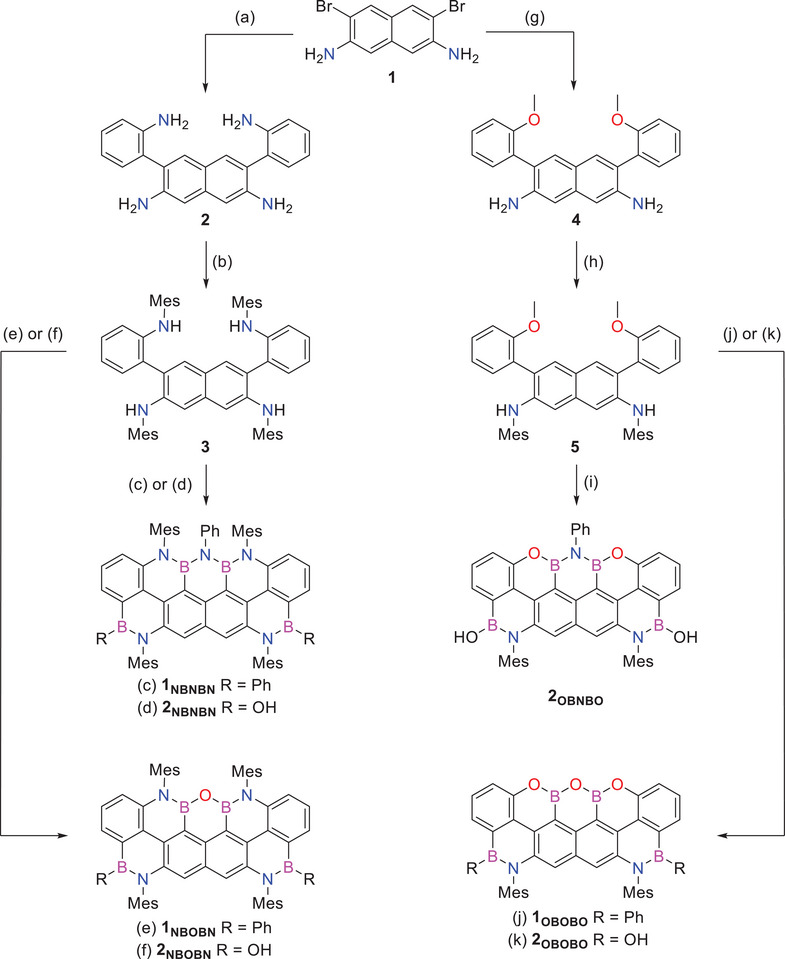
Synthesis of BO/BN‐doped (2,5,4)‐trapeziumenes. Reagents and conditions: (a) 2‐aminophenylboronic acid pinacol ester, [Pd(PPh_3_)_4_], Na_2_CO_3_, toluene/EtOH/H_2_O 3:1:1, 95 °C, 20 h, 80%; (b) 2‐bromomesitylene, [Pd_2_(dba)_3_], *rac*‐BINAP, *^t^* BuONa, toluene, 110 °C, 72 h, 60%; (c) I. BBr_3_, TCB, 240 °C, 18 h; II. PhMgBr, THF, rt, 18 h; III. PhNH_2_, TCB, 230 °C, 48 h, 13%; (d) I. BBr_3_, TCB, 240 °C, 18 h; II. PhNH_2_, toluene, 110 °C, 72 h; III. H_2_O, rt, 1 h, 70%; (e) I. BBr_3_, TCB, 240 °C, 18 h; II. PhMgBr, THF, rt, 18 h; III. H_2_O, 70 °C, 1 h, 72%; (f) I. BBr_3_, TCB, 240 °C, 18 h; II. H_2_O, rt, 1 h, 90%; (g) 2‐methoxyphenylboronic acid pinacol ester, [Pd(PPh_3_)_4_], Na_2_CO_3_, toluene/EtOH/H_2_O 3:1:1, 95 °C, 20 h, 31%; (h) 2‐bromomesitylene, [Pd_2_(dba)_3_], *rac*‐BINAP, *^t^* BuONa, toluene, 110 °C, 24 h, 89%; (i) I. BBr_3_, DCB, 200 °C, 18 h; II. PhNH_2_, toluene, 120 °C, 18 h, 16%; (j) I. BBr_3_, TCB, 240 °C, 18 h; II. PhMgBr, THF, rt, 18 h; III. H_2_O, 70 °C, 1 h, 56%; (k) I. BBr_3_, TCB, 240 °C, 18 h; II. H_2_O, THF, 70 °C, 2 h, 36%.

The synthetic efforts started with the preparation of the NBNBN‐ and NBOBN‐doped (2,5,4)‐trapeziumenes, **1_NBOBN_** and **1_NBNBN_** (Scheme 1). Suzuki‐Miyaura cross‐coupling reaction between dibromonaphthalene‐2,7‐diamine **1** and 2‐aminophenylboronic ester in the presence of [Pd(PPh_3_)_4_] and Na_2_CO_3_ gave naphthalene derivative **2** in 80% yield. Successive Buchwald–Hartwig N‐arylation reaction of tetraamino‐naphthalene **2** with MesBr in the presence of [Pd_2_(dba)_3_], *rac*‐BINAP, *^t^* BuONa gave tetramesitylated derivative **3** in 60% yield. The boron atoms were inserted through N‐directed borylation of **3** with excess BBr_3_ under anhydrous and O_2_‐free conditions at 240 °C. To our surprise, the addition of aniline followed by that of PhMgBr did not yield any product. Instead, reversing the addition sequence, i.e. first PhMgBr and then aniline, and heating under refluxing conditions (230 °C for 48 h) gave the NBNBN‐doped trapeziumene **1_NBNBN_** in 13% over three steps (Scheme 1). When H_2_O is added instead of aniline and the mixture is heated to 70 °C, the NBOBN‐doped trapeziumene **1_NBOBN_** is obtained in good yield (72%) over three steps. From these results, we hypothesize that the initial borylation step generates a tetrabromoborane intermediate. When aniline is added at this stage, we believe that aminoborane derivatives are formed. Upon subsequent treatment with a Grignard reagent, deprotonation is likely to occur, rendering the boron center less electrophilic and thus limiting further transformations. On the other hand, when PhMgBr is introduced first, we postulate the formation of a tetraphenylborane intermediate. In this scenario, the formation of strong B─O bonds during hydrolysis likely drives the selective cleavage of B─C(Ph) bonds in *peri*‐positions, promoting the assembly of the BOB motif. In contrast, aminolysis appears less favorable under these conditions, possibly due to the intrinsically weaker B─N bond formation. In the synthesis of OBOBO‐ and OBNBO‐doped trapeziumenes **1_OBOBO_** and **1_OBNBO_**, dibromonaphthalene‐2,7‐diamine **1** was instead reacted with *o*‐Bpin‐2‐methoxyphenyl under the same Suzuki cross‐coupling conditions used in the previous cases.

This reaction afforded the naphthalene derivative **4** in 31% yield. Successive Buchwald–Hartwig cross‐coupling reaction with MesBr, gave tetramesitylated product **5** in excellent yield (81% yield, Scheme 1). Borylation with BBr_3_ under moisture‐ and air‐free conditions at 240 °C, followed by addition of PhMgBr for 18 h and subsequent quenching with water, afforded trapeziumene **1_OBOBO_** in good yield (55%). Surprisingly, when aniline was added instead of water, the expected compound **1_OBNBO_** could not be isolated, with only **1_OBOBO_** being recovered. Variations in reaction temperature and inversion of the addition sequence did not lead to the formation of **1_OBNBO_**. However, we noticed that if only aniline is added, without the addition of PhMgBr, in toluene at 120 °C for 18 h, borinic derivative **2_OBNBO_** could be obtained after workup in 16% yield over three steps. Therefore, we decided to prepare the whole series of borinic‐derived trapeziumenes, namely the OBOBO‐, NBNBN‐, and NBOBN‐doped derivatives.

**Figure 2:**
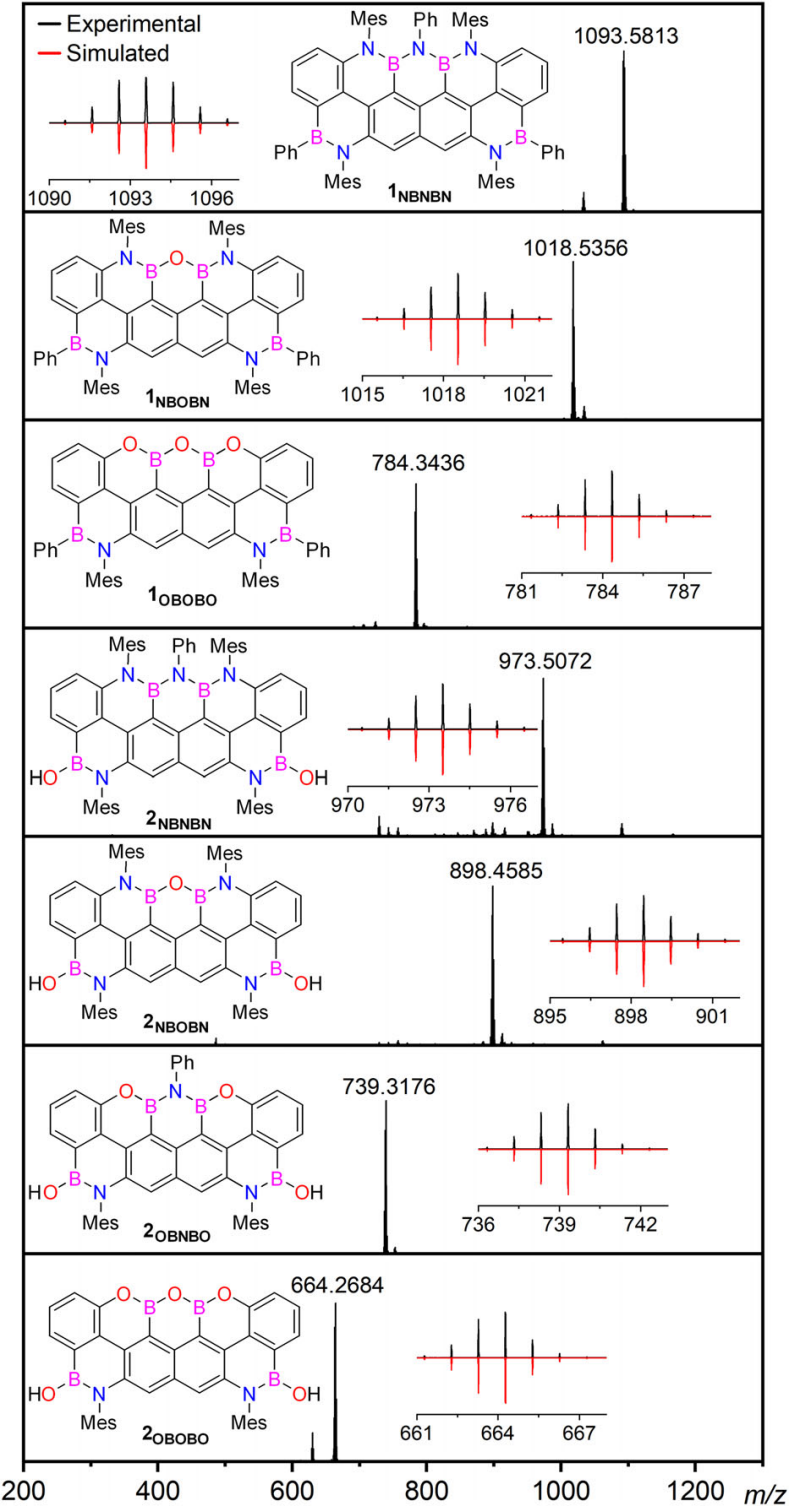
HR MALDI MS (matrix: DCTB) spectra for the BN‐doped nanoribbons **1_NBNBN_**, **2_NBNBN_**, **2_NBOBN_**, and HR LDI MS spectra for **1_NBOBN_**, **1_OBOBO_**, **2_OBNBO_**, and **2_OBOBO_**. Inset: experimental (black) and simulated (red) isotopic pattern of the M^+^ peak.

Thus, molecule **2_OBOBO_** could be easily prepared (36% yield) after the borylation step of **7** upon simple addition of water. As far as **2_NBOBN_** is concerned, after the borylation step of derivative **5**, the simple addition of water led to the targeted product in 90% yield (Scheme 1). Finally, the borinic derivative **2_NBNBN_** could be obtained in 70% yield by treating the borylated intermediate derived from **5** with aniline at 110 °C for 72 h and then with water at rt for one hour. The newly synthesized BN‐doped polycyclic aromatic derivatives were unequivocally identified by HRMS (Figure 2) through the detection of peaks corresponding to the molecular ions at *m*/*z* 1093.5813 (M^+^, C_76_H_67_B_4_N_5_, calc.: 1093.5802), 1018.5356 (M^+^, C_70_H_62_B_4_N_4_O, calc.: 1018.5327), and 784.3436 (M^+^, C_52_H_40_B_4_N_2_O_3_, calc.: 784.3435) for **1_NBNBN_**, **1_NBOBN_**, **1_OBOBO_**, respectively. Molecular ions at *m*/*z* 973.5072 (M^+^, C_64_H_59_B_4_N_5_O_2_, calc.: 973.5070), 898.4585 (M^+^, C_58_H_54_B_4_N_4_O_3_, calc.: 898.4595), 739.3176 (M^+^, C_46_H_37_B_4_N_3_O_4_, calc.: 739.3178), and 664.2684 (M^+^, C_40_H_32_B_4_N_2_O_5_, calc.: 664.2702) were instead detected for borinic derivatives **2_NBNBN,_ 2_NBOBN_**, **2_OBNBO,_** and **2_OBOBO,_** respectively. All products exhibited adequate thermal and chemical stability, which allowed them to be isolated and fully characterized using ^1^H, ^13^C, and ^11^B NMR spectroscopies (see Supporting Information).

### X‐Ray Characterization

**Figure 3:**
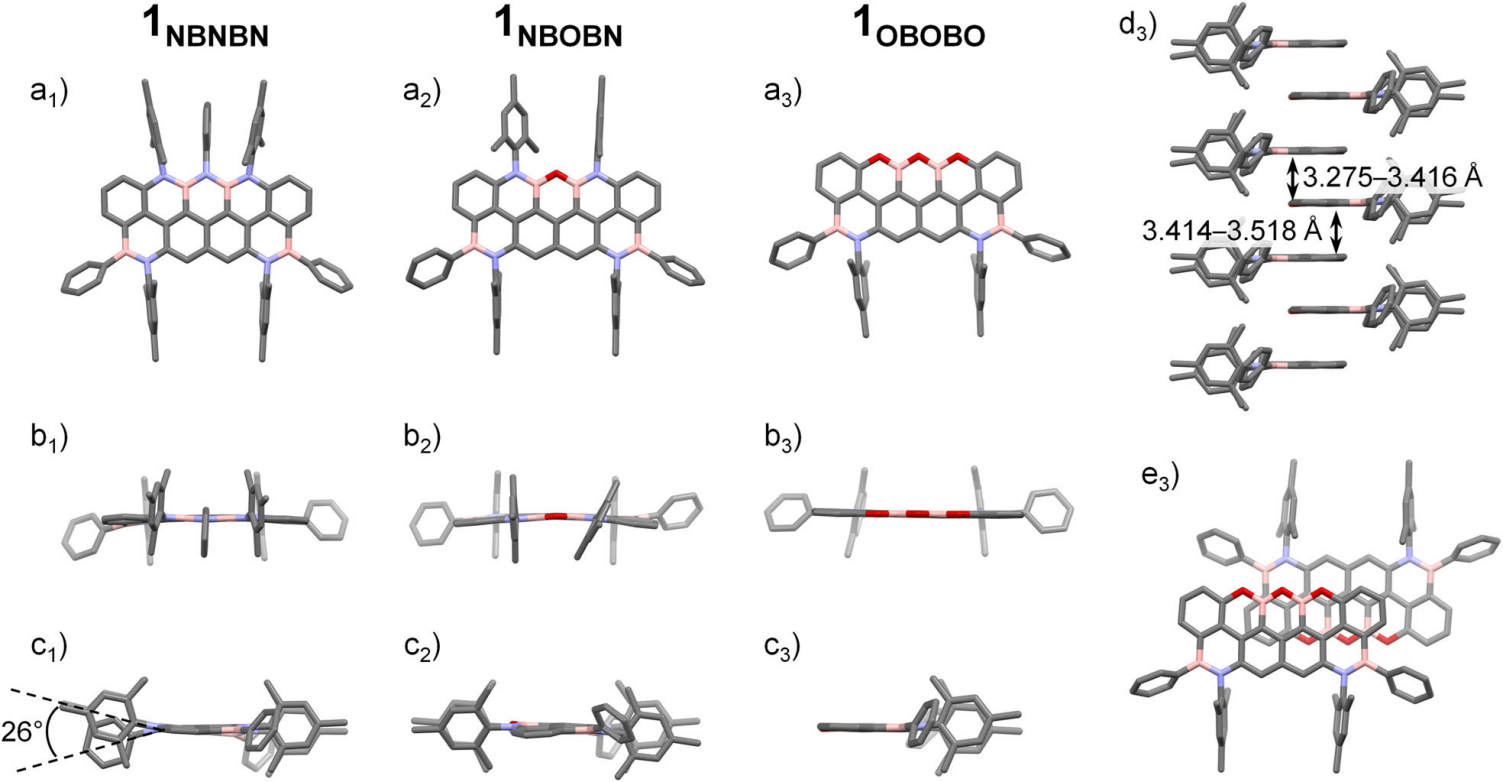
Single crystal X‐ray structures of **1_NBNBN_**, **1_NBOBN,_** and **1_OBOBO_** (from left to right). a) Top view; b) long‐side view; c) short‐side view. For **1_OBOBO_**: (d_3_) solid‐state columnar π–π stacks and (e_3_) *anti* offset π–π stacking arrangement (4.191 Å). Space group: *P*‐1. Crystallization conditions: toluene/CH_3_CN vapor diffusion for **1_NBNBN_** and **1_NBOBN_**, and toluene slow evaporation for **1_OBOBO_**. For **1_NBOBN_**, a single, crystallographically independent molecule is shown. Solid‐state organization of a) **1_NBNBN_** and b) **1_NBOBN_** developing through C─H⋯π interactions. H atoms and solvent molecules are omitted for clarity. Atom colors: gray, C; pink, B; blue, N; red, O.

To further confirm the molecular structures of the phenylborane derivatives, single crystals of **1_NBNBN_**, **1_NBOBN_**, and **1_OBOBO_** were grown and analyzed by X‐ray diffraction. The resulting crystal structures are shown in Figure 3, while detailed crystallographic data are summarized in Tables S2–S4.^[^ ^58^ ^]^ The diffraction analyses corroborate the molecular structures obtained from NMR and HRMS data, further validating the formation of the heteroatomic penta‐doped peripheries. The crystal structures reveal a nearly planar backbone for the three derivatives, with peripheral Mes and Ph groups oriented approximately perpendicularly to the core (Figure 3). Notably, the N‐Mes moieties induce a slight curvature in the heteroaromatic scaffold (Figure 3b), showing maximum deviations from planarity of 8° and 11° for **1_NBNBN_** and **1_NBOBN_**, respectively (no deviations were observed for **1_OBOBO_**).

Furthermore, in **1_NBNBN_**, the central N─Ph unit introduces additional twisting (offset angle with N‐Mes groups: 26°, Figure 3c1), likely due to the repulsion between the *peri*‐N‐aryl groups. The peripheral azaborine‐like B─N bonds exhibit lengths within the 1.400–1.453 Å range, in agreement with analogous BN‐containing structures.^[^ ^51^, ^59^, ^60^, ^61^, ^62^ ^]^ In **1_NBNBN_**, the four alternating B‐N bonds (1.444, 1.480, 1.457, and 1.460 Å) fall between typical single (1.483 Å) and double (1.416 Å) B─N bond lengths,^[^ ^63^ ^]^ indicating partial double bond character. Furthermore, they are slightly elongated compared to the N─B─N motif (1.445 Å),^[^ ^51^ ^]^ suggesting reduced π‐conjugation upon extension of the BN doping pattern. In **1_NBOBN_**, B─N bond lengths range within 1.401–1.426 Å, and B─O within 1.376–1.400 Å. Similarly, in **1_OBOBO_**, the alternating B─O bond lengths (1.392, 1.373, 1.394, and 1.369 Å) align with those observed in B─O and O─B─O doping motifs (1.358–1.393 Å).^[^ ^40^, ^41^, ^64^, ^65^, ^66^ ^]^ Furthermore, a progressive shortening of the B─C bonds is observed across **1_NBNBN_**, **1_NBOBN,_** and **1_OBOBO_**, with average bond lengths of 1.577, 1.548, and 1.521 Å, respectively. Due to the presence of bulky mesityl peripheral groups, no π–π stacking interactions are observed in the crystal packing of **1_NBNBN_** and **1_NBOBN_**. In these crystals, the arrangements predominantly develop through intermolecular C(Methyl)‐H⋯π interactions (3.553–3.832 Å, Figure S92). In contrast, the derivative **1_OBOBO_** assembles into π–π stacks with average interplanar spacings of 3.346 and 3.466 Å (Figures 3d_3_ and S93). The molecules are organized in an antiparallel face‐to‐face manner, exhibiting lateral offsets of approximately 4.191 and 6.739 Å (Figures 3e_3_ and S93).

### Optoelectronic Properties

**Figure 4:**
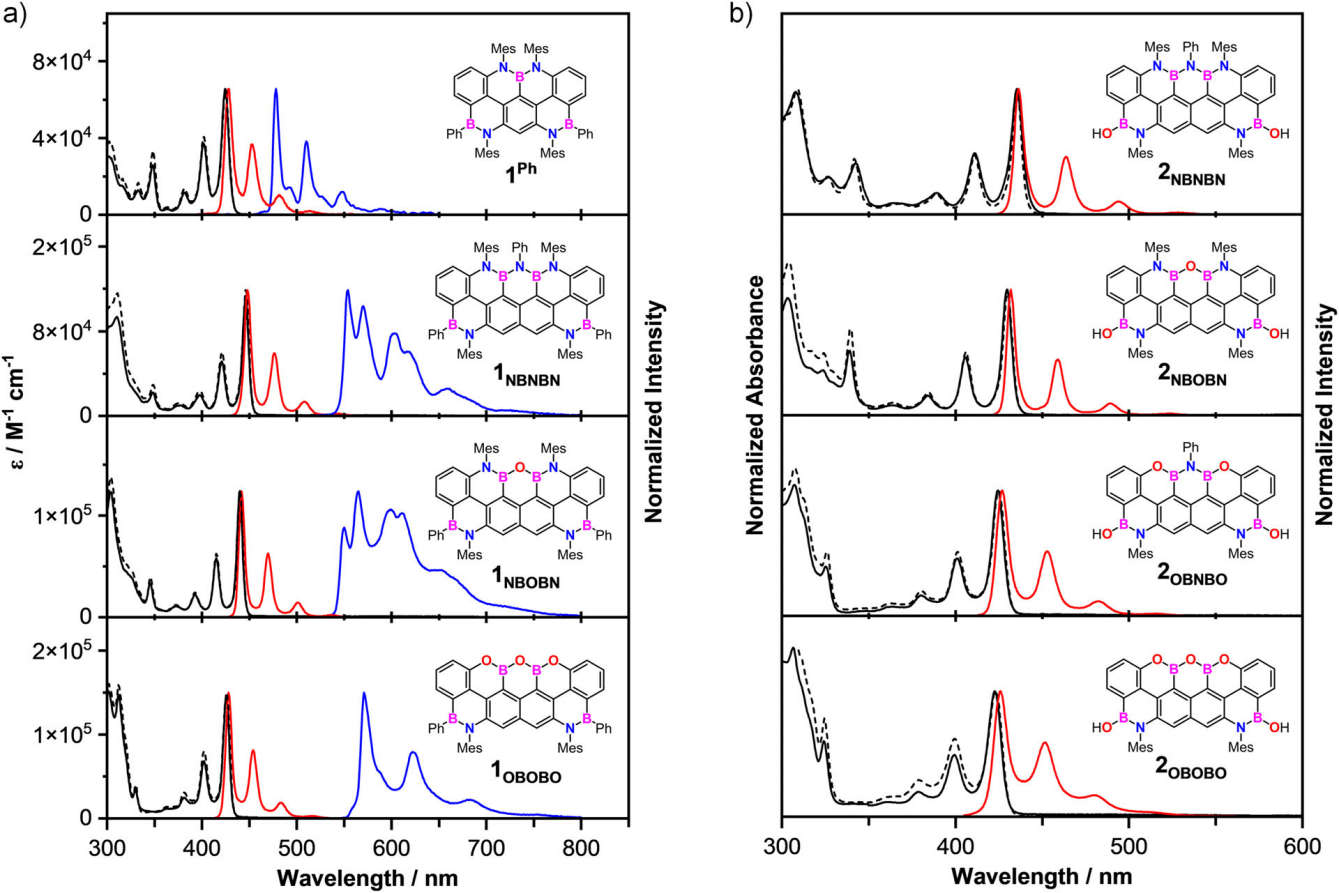
a) Molar absorptivity (solid black), normalized fluorescence emission (solid red), excitation (dashed black), and phosphorescence emission spectra (blue) at 77 K for **1^Ph^** and the **1_y_** series at rt in 2‐MeTHF. b) Normalized absorbance (solid black), normalized fluorescence emission (solid red), and excitation (dashed black) spectra for the **2_y_** series at rt in 2‐MeTHF.

The characterization of the doped trapeziumenes began with the measurement of UV–vis absorption and fluorescence emission spectra for both the phenylborane‐ (**1_NBNBN_**, **1_NBOBN_**, **1_OBOBO_**) and borinic‐derived (**2_NBNBN_**, **2_NBOBN_**, **2_OBNBO_**, **2_OBOBO_**) compounds (Figure 4; Tables 1 and S1). Given the tendency of the borinic moieties to undergo dehydration and form anhydrides in solution, we limited optoelectronic investigations to steady‐state absorption and emission studies at room temperature for these PAHs. The UV–vis absorption spectra of all compounds exhibit structured absorption bands in the 300–450 nm range, characteristic of π → π^*^ electronic transitions. The fluorescence emission spectra closely mirror the absorption spectra, with all compounds exhibiting well‐defined emission bands and minimal Stokes shifts (<3 nm), suggesting limited structural relaxation between the ground and singlet excited states. The previously reported^[^ ^51^ ^]^ derivative **1^Ph^** (Figure 4a) was chosen as a molecular benchmark for evaluating the effect of the π‐extension and heteroatom substitution patterns on the optoelectronic properties of the trapeziumene congeners.

**Table 1:** Experimentally determined photophysical properties.

	Absorbance		Fluorescence						Phosphorescence^a)^			Energy	
	ε (M ^−1^ cm ^−1^ )	λ_max_ (nm)	λ_FL_ (nm)	τ_FL_ (ns) ^a)^	τ_FL_ (ns) ^b)^	Φ_FL_ ^c)^	*k*_fl_ (µs ^−1^ )	*k*_nr_ (µs ^−1^ )	λ_PH_ (nm)	τ_PH_ (s)	Φ_Δ_ ^d)^	*E*_00_ ^e)^ (eV)	Δ *E*_ST_ (eV)
**1_NBNBN_**	111,164	446	448	3.4	3.0	0.99	291	3	556	2.52	0.06	2.77	0.53
**1_NBOBN_**	124,005	440	442	3.4	3.1	0.90	265	29	550	2.90	0.05	2.81	0.54
**1_OBOBO_**	146,022	426	428	3.2	2.6	0.78	244	69	568	1.01	0.06	2.90	0.75

^a)^ Measured at 77 K

^b)^ Measured at rt

^c)^ Absolute quantum yield

^d)^ Relative to C_60_ (Φ_Δ _ =  1)

^e)^ Estimated from the crossing point between the measured absorbance and emission spectra.

Generally, all derivatives **1_Y_** exhibit slightly redshifted absorption profiles compared to reference **1_Ph_** (*λ*_max_ = 425 nm, *E*_00_ = 2.90 eV), with the electronic transitions progressively blueshifted upon substituting N atoms with the more electronegative O counterparts (*λ*_max_ = 446, 440, 426 nm for **1_NBNBN_, 1_NBOBN,_** and **1_OBOBO_**, respectively). This change reflects a widening of the optical band gap (*E*_00_ = 2.77, 2.81, and 2.90 eV, respectively). Each N→O substitution accounts for approximately an energy increase of 40 meV. Similarly, in the case of the borinic derivatives **2_Y_**, a progressive hypsochromic shift was also observed (although less pronounced) upon N→O substitution (*λ*_max_ = 435, 430, 425, and 423 nm for **2_NBNBN_**, **2_NBOBN_**, **2_OBNBO_**, and **2_OBOBO_**, respectively), which accounts for an energetic increase of 0.04‐0.02 eV for each N→O substitution. All phenylborane compounds exhibit strong fluorescence quantum yields (*Φ*_FL_). Among them, **1_OBOBO_** exhibited the lowest quantum yield (0.78), while trapeziumenes **1_NBOBN_** and **1_NBNBN_** show significantly higher *Φ*_FL_ values. Notably, **1_NBNBN_** displays a near‐quantitative quantum yield of 0.99, making it an outstanding fluorophore.

**Figure 5:**
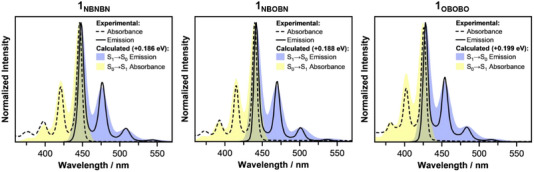
Comparison of the experimental absorption (black dashed) and emission spectra (black solid) of compounds **1_NBNBN_**, **1_NBOBN_**, and **1_OBOBO_**, with the computed vibrationally resolved spectra for the dominant S_0_ → S_1_ absorption (yellow) and the S_1_ → S_0_ emission (blue), computed using the Franck–Condon Herzberg–Teller approach.

To shed light on the low‐energy electronic transitions, absorption and emission spectra of derivatives **1_NBNBN_**, **1_NBOBN_**, and **1_OBOBO_** were computed using time‐dependent density functional theory (TD‐DFT), in combination with the Franck–Condon Herzberg–Teller (FCHT) approach;^[^ ^67^, ^68^ ^]^ the latter allows to incorporate both vibrational effects and vibronic coupling, providing a more accurate simulation of spectral shapes and intensities. The vibrationally resolved absorption spectra were modeled for the S_0_ → S_1_ transition (yellow, Figure 5), which dominates the low‐energy absorption region.

For the emission spectra, only the S_1_ → S_0_ transition (blue, Figure 5) was considered, in accordance with Kasha's rule,^[^ ^69^ ^]^ as fluorescence primarily occurs from the lowest singlet excited state. The computed vibronic profiles accurately reproduced the experimental spectral shapes, including the structured emission bands, and provided a clear explanation for the observed vibronic features. Moreover, the theoretical results closely tracked the experimentally observed blueshifts associated with N→O substitution, confirming that each substitution step results in a systematic widening of the optical gap.

When cooled to 77 K, two independent emissions with different lifetimes were observed and assigned to fluorescence and phosphorescence. As expected, no significant changes in the fluorescence emission properties were detected at 77 K, with only a slight narrowing of the peaks due to further inhibition of thermal deactivation pathways, and a moderate decrease in fluorescence lifetime (*τ*_FL_) likely due to enhanced intersystem crossing (ISC, Table 1). As the temperature‐induced shortening of the *τ*_FL_ values (3.2 ns at rt vs 2.6 ns at 77 K) is very pronounced for the OBOBO derivative, one can anticipate that the presence of the O atoms facilitates ISC more efficiently than the N atoms. The phosphorescence emission (Figure 4) displays a vibronic emission profile for all molecules, with the spectral envelopes red shifting when passing from molecule **1^Ph^** (*λ*_em_ = 478 nm) to **1_y_** (*λ*_em_ = 556, 550, and 568 nm for **1_NBNBN_**, **1_NBOBN_**, and **1_OBOBO_**, respectively), suggesting significant T_1_ stabilization. Interestingly, the most pronounced redshift occurs for the fully O‐doped trapeziumene, which is particularly notable given the fact that this molecule exhibits the most blue‐shifted fluorescence in the series. This behavior suggests that the N → O substitution asymmetrically affects the singlet and triplet states (for an interpretation, see the theoretical account below). Consequently, trapeziumene **1_NBNBN_** has a significantly lower singlet‐triplet energy gap (Δ*E*_ST_ = 0.53 eV) than its O‐containing **1_NBOBN_** (0.54 eV) and **1_OBOBO_** (0.75 eV) analogues. The three compounds exhibit a Δ*E*_ST_ higher than the 0.27 eV observed for the reference compound **1^Ph^**, which remains too large to enable efficient TADF at rt, as effective reverse intersystem crossing (RISC) typically requires Δ*E*_ST_ ≤ 0.4 eV.^[^ ^70^ ^]^ Finally, time‐resolved measurements revealed that the phosphorescence lifetimes (τ_PH_) of trapeziumenes **1_NBNBN_** (2.5 s) and **1_NBOBN_** (2.9 s) are comparable to that of **1_Ph_** (2.2 s). In contrast, **1_OBOBO_** showed a significantly shorter phosphorescence lifetime value of approximately 1.0 s. This suggests that N→O substitution hinders non‐radiative relaxation, likely by reducing vibrational coupling and limiting energy dissipation pathways. Indeed, examination of the singlet state at rt yielded a non‐radiative rate constant of 3 µs^−1^ for **1_NBNBN_**, whereas the O‐containing analogues **1_NBOBN_** and **1_NBNBN_** displayed values of 29 and 69 µs^−1^, respectively, considerably higher than that of **1_NBNBN_**.

Given the presence of emissive triplet states, their ability to sensitize ^1^O_2_ was investigated by determining the sensitization quantum yield (*Φ*_Δ_) at room temperature. To evaluate this, we measured the characteristic phosphorescence emission of ^1^O_2_ centered at 1273 nm and employed the relative quantum yield method using C_60_ as reference (*Φ*_Δ_ = 1).^[^ ^71^ ^]^ The three phenyl borane derivatives exhibited comparable ^1^O_2_ sensitization efficiencies, with *Φ*_Δ_ values of ca. 0.06 for **1_NBNBN_** and **1_OBOBO,_** and 0.05 for **1_NBOBN_**. The low efficiency of ^1^O_2_ generation suggests that ISC to the triplet state is not favored in these systems at rt; thus, singlet emission is the predominant pathway, as supported by the high *Φ*_FL_ values observed.

**Figure 6:**
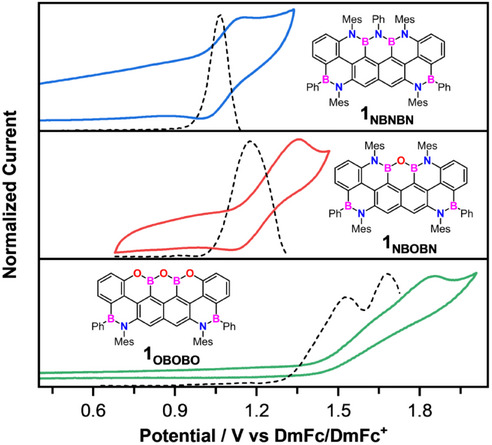
Cyclic voltammetry (full) and differential pulse voltammetry (dashed) traces of **1_NBNBN_** (blue), **1_NBOBN_** (red), and **1_OBOBO_** (green). CV scan rate: 200 mV s^−1^. Solvent: tetrachloroethane. Supporting electrolyte: TBAPF_6_. Working electrode: 7 mm^2^ Pt disk. Counter electrode: Pt wire. DmFc is used as an internal reference standard. The Half‐wave potentials (*E*_1/2_) were calculated as *E*_1/2_ = (*E*_pa_ + *E*_pc_)/2 by considering anodic (*E*_pa_) and cathodic (*E*_pc_) peak potentials.

The electrochemical properties of the phenylborane trapeziumenes were investigated by cyclic voltammetry (CV) and differential pulse voltammetry (DPV) using the redox couple Decamethylferrocene/Decamethylferrocenium (DmFc/DmFc^+^) as reference (Figure 6). All measurements indicated that the trapeziumenes **1y** exhibit p‐type behavior, as only oxidative processes were observed. Initially, CV measurements were performed in *o*‐DCB/CH_3_CN (4:1), with only irreversible oxidation peaks being observed in the range 1.2–1.5 V vs DmFc/DmFc^+^. Given previous success in stabilizing radical cation species in tetrachloroethane,^[^ ^51^ ^]^ additional CV experiments were conducted in this solvent.

**Figure 7:**
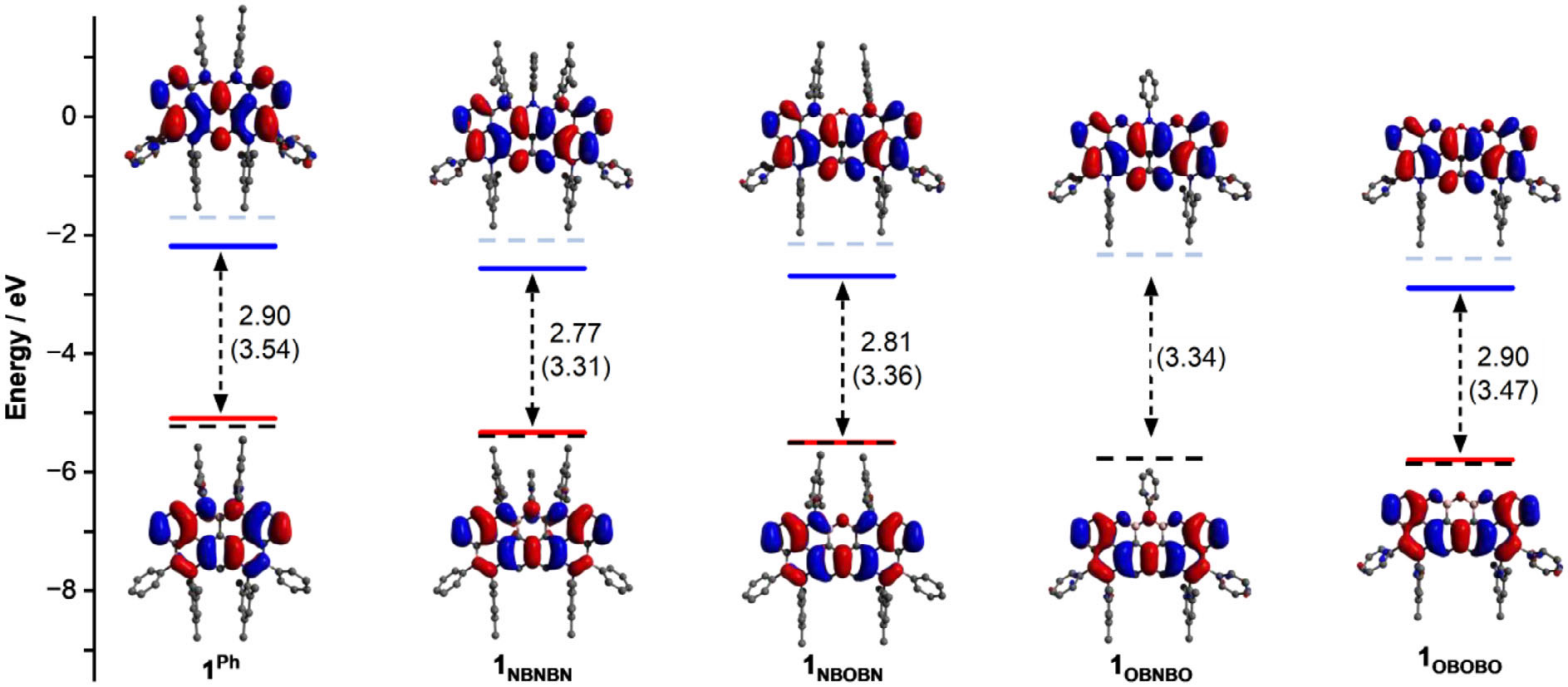
Experimentally estimated (solid lines) HOMO (red) and LUMO (blue) using the formulas *E*_HOMO_ = −(*E*_1/2_^ox^ − 0.54) − 4.8 and *E*_LUMO_ = *E*_HOMO_ + *E*_00_, and computed values (isovalue 0.02) using DFT (dashed lines). Values between parenthesis correspond to the calculated HOMO‐LUMO gaps. The non‐synthesized compound **1**_**OBNBO**_ is included for comparison.

Notably, quasi‐reversible oxidation processes centered at 1.07 and 1.24 V were observed for derivatives **1_NBNBN_** and **1_NBOBN_**, respectively. In contrast, despite numerous attempts, only two irreversible oxidation events were detected for **1_OBOBO_** at 1.53 and 1.63 V, as determined by DPV. As expected, the progressive O‐substitution increases the oxidation potential by ca. 170 meV per N→O replacement, making the PAH progressively a worse p‐type semiconductor. Notably, reference **1^Ph^**, featuring a reduced π‐conjugated surface, exhibits a lower oxidation potential (*E*_1/2_^ox^ = 0.83 V) than its NBNBN‐doped analogue **1_NBNBN_**. From this experimental data, one can experimentally estimate the HOMO energy levels (Figure 7). As expected, N→O   substitution progressively lowers the HOMO energy level (−5.33, −5.50, and −5.79 eV for **1_NBNBN_**, **1_NBOBN_**, and **1_OBOBO_**, respectively), with the fully O‐doped PAH exhibiting the lowest value. The LUMO energy levels, estimated using the *E*_00_ values, follow a similar trend, with **1_NBNBN_** exhibiting the highest LUMO energy at −2.56 eV, followed by **1_NBOBN_** at −2.69 eV and **1_OBOBO_** at −2.89 eV. Notably, the theoretically calculated HOMO‐LUMO gaps (Figure 7, dashed lines and Figure S94) are consistent with the experimental trends, supporting the conclusion that N→O   substitution progressively increases the HOMO–LUMO gap and rigidly shifts the electronic frontier molecular orbital energy levels.

### Theoretical Modelling of the Excited States

**Figure 8:**
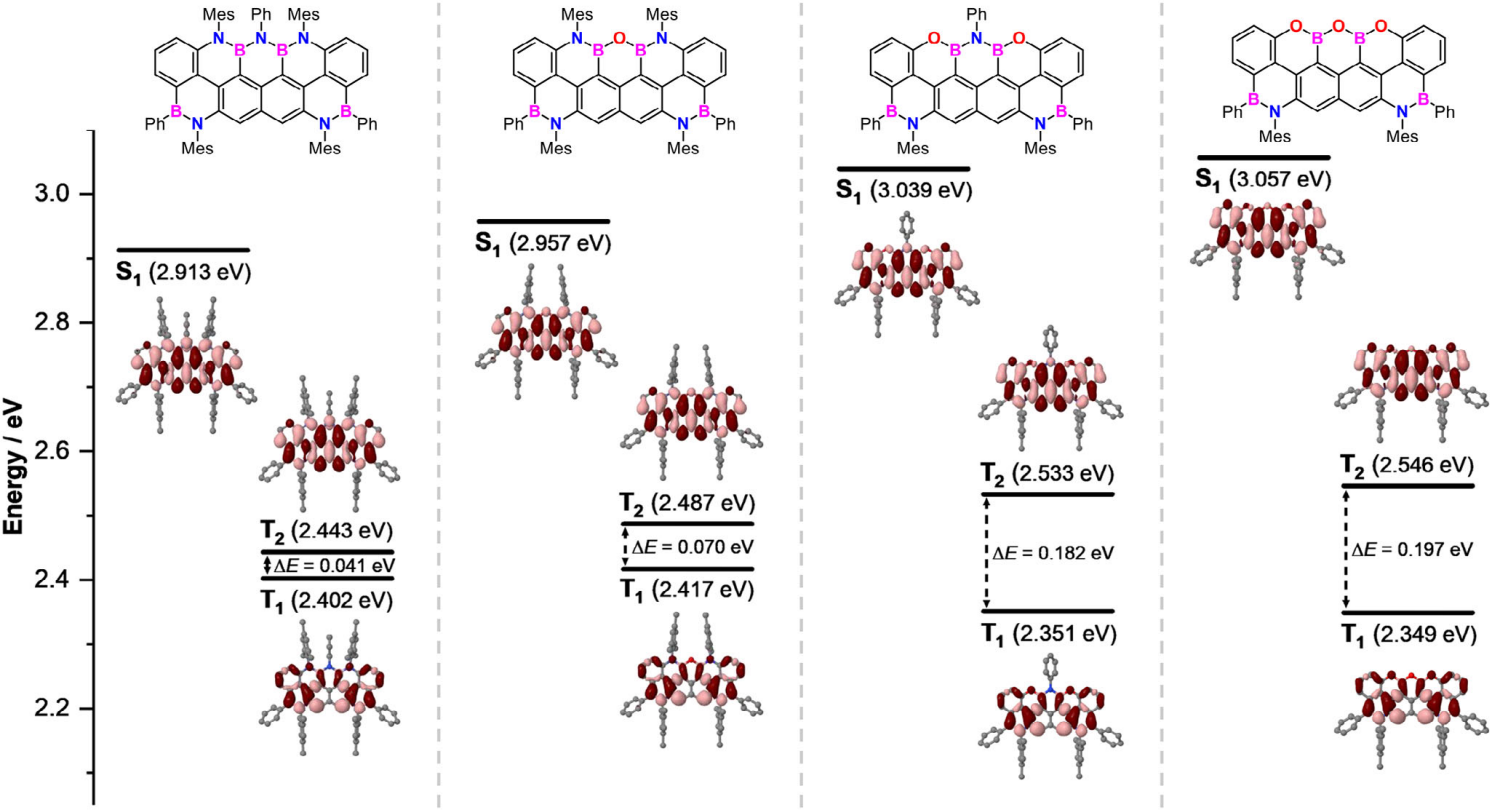
Excited state charge density difference for molecules **1_Y_**, computed as the difference between the excited‐state and ground‐state electron densities, for the S_1_, T_1_, and T_2_ excited states (isovalue 0.0004). Regions shown in dark red indicate charge accumulation upon photoexcitation, whereas regions in light red correspond to charge depletion after photoexcitation.

To gain deeper insight into the influence of peripheral O and N atoms on the excited‐state properties and establish a chemical descriptor that could direct future molecular design, charge density difference maps (Figure 8) were computed (i.e., the difference between the excited‐ and ground‐state electron densities) upon photoexcitation of the PAH framework.^[^ ^72^ ^]^

This approach enabled visualization of regions of electron accumulation and depletion, providing a basis to assess how specific heteroatom substitution patterns modulate intramolecular charge redistribution upon photoexcitation. Furthermore, this analysis offers predictive insight into whether such substitutions are likely to facilitate or hinder charge separation, and thereby contribute to shifts in the energy of electronic excitations.^[^ ^72^ ^]^ The computed charge density difference maps are shown in Figure 8 for the first singlet (S_1_) and triplet (T_1_) excited states, as well as for the second triplet (T_2_) excited state of molecules **1_NBNBN_**, **1_NBOBN_**, and **1_OBOBO_**. For comparative purposes, the phenylborane derivative **1_OBNBO_** was also included in the calculations. The T_2_ state was examined because it was found to lie below S_1_ in energy, suggesting a possible role as an intermediate in the ISC pathway prior to population of T_1_. In these maps, light red indicates regions of charge depletion, whereas dark red denotes areas of charge accumulation following excitation.

The peripheral atoms in the XBXBX (X═N or O) motif exhibit a pronounced and asymmetric electron redistribution upon population of the S_1_ and T_2_ excited states. In contrast, only limited charge redistribution is observed at these positions in the T_1_ state, suggesting that T_1_ is less sensitive to peripheral doping. According to the calculated charge depletion regions (light red, Figure 8), the X atoms appear to function as electron donors in the S_1_ and T_2_ states, while the B atoms act as acceptors. Conversely, in the T_1_ state, an opposite charge distribution is observed, with electron accumulation mainly taking place on the lateral X and B atoms, with the central X atom sandwiched between the two B atoms experiencing no significant charge redistribution. In this configuration, the lateral X and B atoms function as electron acceptors, and the central X heteroatom appears to contribute only through bonding interactions with the adjacent boron atoms.

Based on this analysis, it is proposed that N→O substitution destabilizes the S_1_ and T_2_ excited states by increasing their excitation energies. This effect is reflected in the hypsochromic shifts observed in the UV‐vis absorption and fluorescence emission spectra. In contrast, N→O substitution is expected to induce a slight bathochromic shift in the T_1_→S_0_ phosphorescent emission, as electron accumulation takes place at the lateral X positions in this specific excited state.

In addition to the charge redistribution maps, Figure 8 also presents the calculated vertical excitation energies for the S_1_, T_2_, and T_1_ excited states (see also Figure S95). This data reveals a progressive widening of the T_2_‐T_1_ energy gap upon N→O substitution, with Δ*E*(T_2_‐T_1_) increasing from 41 meV in **1_NBNBN_** to 70 meV in **1_NBOBN_**, 182 meV in **1_OBNBO_**, and reaching a maximum of 197 meV in **1_OBOBO_**. This trend reflects the increasing differentiation in spatial distribution and electronic character of the two triplet states as O atoms are introduced. The increased energetic separation between T_2_ and T_1_ is consistent with the enhanced charge redistribution observed in the T_1_ state of O‐rich derivatives, suggesting that incorporation of O atoms leads to more localized and electronically distinct triplet excited states, thereby widening the T_2_–T_1_ gap.

## Conclusions

In summary, we have reported the synthesis of a new family of penta‐heteroatom‐doped *peri*‐acenoacenes, the (2,5,4)‐trapeziumenes, featuring diverse NBNBN, NBOBN, OBNBO, and OBOBO doping sequences. The combination of Suzuki–Miyaura and Buchwald–Hartwig couplings with heteroatom‐directed borylation enabled the selective preparation of both phenylborane and borinic derivatives. This synthetic platform provided precise control over the doping pattern, allowing systematic modulation of the optoelectronic properties. Peripheral N→O substitution was found to progressively widen the optical gap, increase singlet–triplet energy separation, and modulate both the fluorescence quantum yield and the redox potentials.

Complementary theoretical investigations provided a detailed conceptual framework to rationalize these trends. The computed charge density difference maps reveal that N→O substitution asymmetrically affects the singlet and triplet excited states: while incorporation of O atoms raises the S_1_ excited‐state energy level by ca. 40–43 meV, it stabilizes the T_1_ manifold by ca. 33–36 meV through enhanced stabilization of charge accumulation at the heteroatom positions. This opposing effect results in a simultaneous blue shift in fluorescence and a red shift in phosphorescence, as experimentally observed. Furthermore, the calculations unveiled a progressive widening of the T_2_–T_1_ energy gap upon O‐doping, providing insight into the electronic decoupling of triplet states. Taken together, the results show that BN/BO‐doped trapeziumenes combine oxidative stability with tunable electronic levels, supporting their realistic potential as robust p‐type organic semiconductors. Their exceptional emissive efficiencies, which are tunable, make them promising for emissive devices and optoelectronic applications, and will constitute the focus of future investigations.

We believe that this work represents a significant step toward developing synthetic protocols for rationally accessing the full peripheral ceramization of PAHs, thereby providing a pathway to integrate the chemical robustness of inorganic ceramics with the functional diversity of PAHs. The ability to precisely tailor both structure and function at the molecular level opens up new avenues for designing stable, photochemically customized molecular scaffolds for applications in organic electronics, photonics, and photocatalysis.

## Supporting Information

The authors have cited additional references within the Supporting Information.^[^ ^73^, ^74^, ^75^, ^76^, ^77^, ^78^, ^79^, ^80^, ^81^, ^82^, ^83^, ^84^, ^85^, ^86^, ^87^, ^88^, ^89^, ^90^, ^91^ ^]^

## Author Contributions

DP: Synthesis, investigation, structural characterisation, final review, supporting information. MM: Synthesis, investigation, supporting information, final review. DHC: Theoretical calculations, validation, methodology, formal analysis, writing original manuscript, visualisation, and funding acquisition. RRF: Spectroscopic characterisation, electrochemistry, data curation, writing original manuscript. MC: X‐ray acquisition, relative data analysis, and writing of the section. PKM: X‐ray acquisition and relative data analysis. LG: Supervision, review & editing, funding acquisition. DB: Conceptualisation and project design, project administration, supervision, writing original manuscript, review and editing, and funding acquisition.

## Conflict of Interests

The authors declare no conflict of interest.

## Supporting information



Supporting Information

Supporting Information

Supporting Information

## Data Availability

The data that support the findings of this study are available in the Supporting Information of this article.
