# Supplementary material for: BN/BO Doping of peri‐Acenoacenes: Modulating Excited States in Trapeziumene Congeners
Source: Angew Chem Int Ed Engl. 2025 Sep 16;64(44):e202517114. doi: 10.1002/anie.202517114 (PMC12559455; doi:10.1002/anie.202517114)
Supplement: Supplementary file 1 — Supporting Information [file ANIE-64-e202517114-s003.pdf]

Supporting Information

B-N/B-O Doping of peri-Acenoacenes: Modulating Excited States in Trapeziumene Congeners

Daniele Poletto,[a,b] Mauro Marongiu,[a] David Hernández-Castillo,[b,c] Rúben R. Ferreira,[a,b] Martina Crosta,[a,b] Pradip Kumar Mondal,[d] Leticia González,[c] and Davide Bonifazi*[a]

[a] D. Poletto, Dr. M. Marongiu, R. R. Ferreira, Dr. M. Crosta, Prof. Dr. D. Bonifazi
Institute of Organic Chemistry, University of Vienna, 1090 Vienna, Austria. E-mail: davide.bonifazi@univie.ac.at

[b] D. Poletto, Dr. M. Marongiu, R. R. Ferreira, Dr. M. Crosta, Dr. D. Hernández-Castillo
Doctoral School in Chemistry (DoSChem), University of Vienna, Währinger Straße 42, 1090 Vienna, Austria.

[c] Dr. D. Hernández-Castillo, Prof. Dr. Dr. L. González
Institute of Theoretical Chemistry, Faculty of Chemistry, University of Vienna, Währinger Straße 17, 1090 Vienna, Austria.

[d] Dr. P. Kumar Mondal
Elettra – Sincrotrone, S.S. 14 Km 163.5 in Area Science Park, Basovizza 34149, Trieste, Italy.

Table of Contents

1. General remarks 3

2. Synthetic procedures 10

3. NMR spectra 21

4. Mass spectra 39

5. Photophysical characterization 50

6. Electrochemical characterization 71

7. Crystallographic data 72

8. Computational studies 76

9. References 77

1. General remarks

Instrumentation

Thin layer chromatography (TLC) was conducted on pre-coated aluminum sheets with 0.20 mm Merck Millipore Silica gel 60 with fluorescent indicator F254. TLC plates were visualized by exposure to ultraviolet light (254 or 366 nm).

Column chromatography was carried out using Merck Gerduran silica gel 60 (particle size 40-63 µm) or Merck aluminum oxide 90 active neutral (particle size 63-200 µm, deactivated with 5% of H_2O).

Recycling preparative HPLC was performed using a LaboACE LC-7080 equipped with two GPC columns JAIGEL-2HR Plus.

Melting points (mp) were measured on a Leica Galen III microscope equipped with a heating block and a Hg thermometer (T_{max} = 250 °C) on a microscope slide under air and are uncorrected. According to the apparatus's limitations, the melting points of compounds that did not melt or decompose (dec) up to 250 °C are presented as "> 250 °C."

Nuclear magnetic resonance (NMR) characterizations were performed at the NMR centre of the University of Vienna. NMR spectra were recorded on Bruker spectrometer AV III HD 700, AV III 600 or AV NEO 400. ^{1}H NMR spectra were obtained at 700, 600, or 400 MHz, ^{13}C NMR spectra at 176, 151, or 101 MHz, and ^{11}B NMR spectra at 193 MHz in a quartz NMR tube. All spectra were obtained at room temperature. Carbon spectra were acquired with a complete decoupling for the proton. Proton and carbon chemical shifts are reported in parts per million (ppm, δ scale) according to tetramethylsilane ($\delta_H = \delta_C = 0$ ppm) using the solvent residual signal as an internal reference ($CDCl_3$: δ_H = 7.26 ppm, δ_C = 77.16 ppm; DMSO-*d*$_6$: δ_H = 2.50 ppm, δ_C = 39.52 ppm; CD_2Cl_2: δ_H = 5.32 ppm, δ_C = 54.00 ppm). Boron chemical shifts are reported in ppm, referenced to the external standard boron signal of $BF_3{\cdot}Et_2O$ (δ_B = 0 ppm). Coupling constants (*J*) are given in Hz. Resonance multiplicity is described as s (singlet), d (doublet), dd (doublet of doublets), ddd (doublet of doublets of doublets), t (triplet), td (triplet of doublets), m (multiplet) and bs (broad signal).

Infrared spectra (IR) were recorded on a Bruker Alpha FT-IR spectrometer in ATR mode. Selected absorption bands are reported in wavenumbers (cm^{-1}).

High-resolution mass spectrometry (HRMS) analyses were performed at the Mass Spectrometry Centre of the University of Vienna. ESI mass spectra were obtained on a Bruker maXis UHR ESI-Qq-TOF mass spectrometer or on a Thermo Scientific Orbitrap Exploris 120 mass spectrometer in the positive ion mode, GC mass spectra on an Agilent 7200B GC/Q-TOF mass spectrometer, LD and MALDI mass spectra on a Bruker Autoflex Speed LD-timsTOF or MALDI-timsTOF (matrix: 2-[(2E)-3-(4-tert-butylphenyl)-2-methylprop-2-enylidene]malononitrile (DCTB)) mass spectrometer.

Ultraviolet-visible absorption spectroscopy (UV-vis) was recorded on a Cary 5000 UV-vis-NIR Spectrophotometer (Agilent Technologies, US) running in double beam mode with a matched pair of quartz absorbance cuvettes (1 x 1 cm). All absorption measurements were performed at 21 °C unless specified otherwise. The absorbance of oxygen-free solutions was measured using a custom-built quartz cuvette. The molar attenuation coefficient (ε) was determined by dissolving a known amount of compound and diluting the resulting stock solution to achieve 5 solutions with an appropriate concentration for measurements (absorbance < 1). The plot of absorbance versus concentration was fitted with a linear function and the molar attenuation coefficient was obtained from the slope.

UV-vis emission spectroscopy. The photoluminescence (PL) excitation and emission spectra, absolute quantum yield, and decay curves were recorded on a FLS1000 photoluminescence spectrometer (Edinburgh Instruments, UK). The spectrometer was equipped with excitation and emission double grating Czerny-Turner monochromators, a photomultiplier detector with extended near-infrared sensitivity (PMT-980), fitted with a gating circuit, thermoelectrically cooled to -20 °C with a fan-assisted Peltier element, and a High Speed PMT detector with a response width < 180 ps operating at 0 °C. All samples were prepared in air-equilibrated extra dry 99+% 2-methyltetrahydrofuran (Thermo Fisher, stabilizer free). The maximum absorbance of all solutions was adjusted to < 0.1 to avoid inner filter effect. For oxygen-free measurements, the solutions were degassed by performing multiple cycles of freeze-pump-thaw (typically 5), and the headspace was filled with nitrogen (≥ 99.999%). Low temperature (77 K) measurements were carried out using an Optistat DN liquid nitrogen cryostat (Oxford Instruments, UK) fitted in the FLS1000 spectrometer sample chamber. The samples were placed in a custom-built cryogenic 1x1 cm quartz cuvette and held in a static nitrogen atmosphere. Glassy matrix was achieved by slowly lowering the temperature (20 °C/min). For

steady-state measurements, the samples were excited using a 450 W ozone-free continuous Xenon arc lamp. Time-resolved measurements in the ns range were performed by irradiating the samples with a nano-pulsed laser (EPL-405) and acquired using the High Speed PMT detector in Time-Correlated Single Photon Counting (TCSPC) mode. The tail portion of the decay curves were fitted using the FAST software (Edinburgh Instruments, UK), following a single exponential model with y-offset (background-offset):

$$\mathrm{I(t)} = \mathrm{A} + \mathrm{B} \cdot \mathrm{e}^{\frac{-\mathrm{t}}{\tau}} \tag{1}$$

where A is the y-offset, B the pre-exponential factor, and τ the lifetime. For decays close to the pulse width of the light source, the instrument response function (IRF) was measured using a Ludox® solution (at room temperature) or using the sample itself (at low temperature). In both cases the count rate was adjusted using a computer-controlled neutral density filter wheel in order to match the count rate of the sample emission. In these cases, the decay lifetime was obtained by performing a reconvolution fit using the FAST software. Absolute quantum yields were measured using an integrating sphere (internal diameter 120 mm) fitted on the FLS1000 sample chamber. The samples and blank reference (solvent) were placed in a 1 x 1 cm fluorescence quartz cuvette and the calculations were done using the "direct excitation" method following the equation:

$$\Phi = \frac{\mathrm{E_B} - \mathrm{E_A}}{\mathrm{S_A} - \mathrm{S_B}} \tag{2}$$

where E_B and E_A correspond to the integrated fluorescence emission of the sample and blank reference (solvent), respectively. S_A and S_B refer to the integrated excitation scatter region of the reference and the sample, respectively. For measuring the scatter region, and avoid detector saturation, a neutral density filter (OD = 2) was placed between the integrating sphere exit and the detector to attenuate the signal. A fixed excitation bandwidth of 3 nm was used to ensure the determination of the sample absorption with high accuracy (step = 0.1 nm), while the emission bandwidth was chosen to obtain a strong sample emission signal (peak emission > 10^4 cps). The optical bandgap E_{g}^{00}, was calculated using the intercept of the excitation and emission spectra (λ_{00}) following equation:

$$E_{\mathrm{g}}^{00}(\mathrm{eV}) = 1240/\lambda_{00} \tag{3}$$

Electrochemical analysis Cyclic and differential pulse voltammetry experiments were performed at room temperature in CH_2Cl_2 purified by a SPS system (MBraun, DE), using an Autolab PGSTAT204 potentiostat (Metrohm, DE). A conventional three-electrode electrochemical cell connected to an argon source and an oil bubbler was used. Dry argon gas was bubbled through the sample solution for at least 15 min prior to each measurement and the headspace was continuously flushed throughout the experiment. A pre-bubbler filled with solvent was used in order to prevent evaporation. Platinum disk (3 mm diameter) was used as a working electrode, Pt wire as auxiliary electrode, and an Ag/AgCl electrode as reference.

The platinum working electrode was polished on a pad using alumina slurry and washed with deionized water before each experiment; the Pt wire was flame-cleaned. Tetrabutylammonium hexafluorophosphate (Alfa Aesar, $TBAPF_6$) was twice recrystallized from absolute ethanol prior to use and it was added to the solution as a supporting electrolyte at a concentration of 0.1 M. Ferrocene (Sigma Aldrich) was used as an internal reference. The formal redox potentials (half-wave potentials) were calculated using the formula:

$$E_{1/2} = \frac{E_{\mathrm{pa}} + E_{\mathrm{pc}}}{2} \quad (4)$$

where E_{pa} is the peak anodic potential and E_{pc} is the peak cathodic potential. The energy of the HOMO was estimated using the following equation:

$$E_{\mathrm{HOMO}}(\mathrm{eV}) = -\left(E_{1/2}^{\mathrm{ox}}\right) - 4.8 \quad (5)$$

where E_{ox} is the half-wave oxidation potential (vs Fc/Fc^+) and 4.8 is the HOMO energy of ferrocene in vacuum.

X-ray measurements of $\mathbf{1_{NBOBN}}$ were performed at the XRD1 beamline of the Elettra Synchrotron, Trieste (Italy).[1] The crystals were dipped in NHV oil (Jena Bioscience, Jena, Germany) and mounted on the goniometer head with nylon loops (MiTeGen, Ithaca, USA). Complete datasets were collected at 100 K (nitrogen stream supplied through an Oxford Cryostream 700). Data were acquired using a monochromatic wavelength of 0.70 Å through the rotating crystal method on a Pilatus 2M hybrid-pixel area detector (DECTRIS Ltd., Baden-Daettwil, Switzerland). The diffraction data were indexed and integrated using XDS.[2] The structure was solved with Olex2[2] by using ShelXT[3-4] structure solution program by Intrinsic Phasing and refined as twin with the ShelXL[5] refinement package using least-squares

minimization. In the last cycles of refinement, non-hydrogen atoms were refined anisotropically. Hydrogen atoms were included in calculated positions, and a riding model was used for their refinement. Data collections for $\mathbf{1}_{\mathbf{NBNBN}}$ and $\mathbf{1}_{\mathbf{OBOBO}}$ were performed at the Centre for X-ray Structure Analysis of the University of Vienna. X-ray intensity data were measured at 100 K on a STOE Stadivari diffractometer equipped with dual radiation source Mo and Cu Kα, and a Dectris EIGER2 R 500K detector. The structures were solved *ab initio* and refined by full-matrix least-squares techniques. Hydrogen atoms were inserted at calculated positions using AFIX instructions, while all other atoms were refined with anisotropic displacement parameters. In the case of $\mathbf{1}_{\mathbf{OBOBO}}$, the crystal was mounted from toluene, as degradation in the mounting oil was observed. Indeed, multiple solvent molecules surround $\mathbf{1}_{\mathbf{OBOBO}}$ in the crystal, for which one heavily disordered toluene had to be refined with isotropic displacement parameters. For $\mathbf{1}_{\mathbf{NBNBN}}$, toluene has been modelled as disordered, while the SQUEEZE command had to be applied for disordered acetonitrile molecules present in the lattice. The following software was used: STOE software package for collecting crystal data and image processing, STOE LANA for scaling and absorption correction,[6] SHELXT-2018/2 for structure solution,[3-4] SHELXL-2018/3 for structure refinement,[5] SHELXLE version 1378 and OLEX2-1.5 as graphical user interfaces.[2, 7] Crystal data, data collection parameters, and structure refinement details are given in Tables S2–S4. Structures have been deposited in the Cambridge Structural Database (CSD) with the following deposition numbers: 2268752 ($\mathbf{1}_{\mathbf{NBOBN}}$), 2279843 ($\mathbf{1}_{\mathbf{NBNBN}}$), and 2331294 ($\mathbf{1}_{\mathbf{OBOBO}}$). These data are provided free of charge by the joint Cambridge Crystallographic Data Centre and Fachinformationszentrum Karlsruhe Access Structures service via www.ccdc.cam.ac.uk/structures.

Quantum chemical calculations

A restricted Kohn-Sham formalism was employed to optimize the singlet ground state geometries. The optimizations were performed with the B3LYP hybrid density functional[8-9] together with 6-311G** basis set[10] and Grimme's D3BJ dispersion correction.[11] Solvent effects (dicloromethane) were included implicitly with the conductor-like polarizable continuum model.[12-13] All calculations were performed in Gaussian 16 rev.c01 software.[14]

Excited-state calculations were carried out using the time-dependent framework of DFT (TD-DFT), applying the Tamm–Dancoff approximation (TDA-DFT),[15-16] whenever specified, to improve the numerical stability and reliability of the excited-state

description. Under the vertical (Franck–Condon) picture, all quantities tied to absorption (vertical transition energies, oscillator strengths, and transition-dipole moments) are evaluated at the fully optimized ground-state (S_0) geometry. In this case, a total of 30 singlet excited states were calculated. The reported triplet excited state energies were also computed within the Franck-Condon picture. Emission properties, by contrast, are estimated at the relaxed first-singlet excited-state (S_1) structure, reflecting the assumption that higher electronic states undergo ultrafast internal conversion to S_1 before radiative decay. The convergence of geometry optimizations towards a minimum was validated by the absence of imaginary frequencies within the harmonic approximation, for all ground and excited state potential energy surfaces.

To resolve vibrational fine structure, we compute Franck–Condon–Herzberg–Teller (FCHT) spectra using the Barone protocol as implemented in *Gaussian 16* rev.c01.[17-18] This approach extends beyond conventional harmonic analysis, incorporating the first-order derivative of the transition dipole moment with respect to normal coordinates. It captures both bright (symmetry-allowed) and dark (symmetry-forbidden but vibronically enabled) transitions. Normal-mode analyses are therefore conducted at both S_0 and S_1 equilibrium geometries. Finally, the discrete spectra obtained from TD-DFT and FCHT are convoluted with Gaussian functions using a full width at half-maximum (fwhm) of 0.09 eV, to yield a smooth comparison with the experimental line shapes.

To analyze and visualize hole-electron distribution for the electronic excitations S_1, T_1 and T_2, the Multiwfn v3.8-dev software package was used.[19] The charge density difference (CDD) between the excited state and the ground state can be evaluated as:

$$\Delta\rho(\mathbf{r}) = \rho^{ele}(\mathbf{r}) - \rho^{hole}(\mathbf{r})$$

These calculations were done using unrelaxed excited state density (which is directly constructed by MOs and excited state configuration coefficients). Values of $\Delta\rho(\mathbf{r}) > 0$ were represented with dark-red (charge accumulation upon excitation), while regions with $\Delta\rho(\mathbf{r}) < 0$ were represented in light-red (charge depletion upon excitation).

Materials and methods

Chemicals were purchased from Sigma Aldrich, Acros Organics, TCI, Alfa Aesar, Fluorochem, Thermo Fisher Scientific, and BLDpharm and used without further purification. Mesityl bromide and aniline were distilled over CaH_2 and stored in an argon-filled glove box. Anhydrous 1,2,4-trichlorobenzene (TCB) was purchased from Sigma Aldrich and used as received. Anhydrous toluene and tetrahydrofuran (THF) were dried on an MBraun SPS-800 solvent purification system, degassed, and stored over activated 4 Å molecular sieves. Deuterated solvents were purchased from Eurisotop. Anhydrous conditions were achieved by drying glassware in an oven at 120 °C for at least 8 h and flaming the reaction vessels with a heat gun under vacuum and purging with argon. The inert atmosphere was maintained using argon-filled balloons equipped with a syringe and needle to penetrate the silicon septa used to close the flask's necks. The addition of liquid reagents was performed using argon-purged plastic or glass syringes. Alternative to the use of Schlenk line techniques, inert conditions were achieved by using an argon-filled MBraun LabStar glove box. Solutions were degassed by bubbling argon or freeze-pump-thaw procedure: solutions were frozen in liquid nitrogen and kept under vacuum for 10–15 min before thawing. High-temperature (> 180 °C) reactions were carried out in metal baths. When heating in a closed flask, the reactions were performed in Schlenk tubes filled with argon and opportunely closed with glass stoppers and polyfluorinated grease for guaranteeing gas-tight joints, or in Schlenk tubes with PTFE screwcap or in pressure tubes with PTFE screwcap. For high-temperature borylations with BBr_3 (> 180 °C), a Schlenk pressure tube with PTFE screwcaps sealed with an FFKM O-ring was employed, and the reaction mixture was stirred using a glass-coated magnetic stir bar.

2. Synthetic procedures

Molecule 1

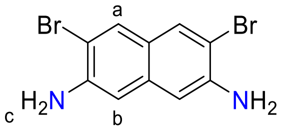

The molecule was synthesized following the procedure reported by Staab et al.[20] Yield: 51%.

^{1}H NMR (400 MHz, DMSO-d_6) δ (ppm): 7.79 (s, 2H, H_a), 6.75 (s, 2H, H_b), 5.35 (bs, 4H, H_c). **^{13}C NMR** (101 MHz, DMSO-d_6) δ (ppm): 143.8, 134.7, 129.9, 121.8, 107.9, 105.2. **HRMS** (ESI): *m/z* calcd for $[C_{10}H_9N_2Br_2]^+$: 316.9107 $[M+H]^+$; found: 316.9104.

Molecule 2

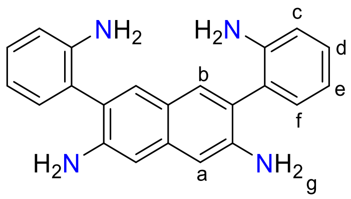

In a 50 mL Schlenk tube were subsequently added 500 mg (1.58 mmol) of **1**, 1.52 g (6.93 mmol) of 2-aminophenylboronic acid pinacol ester, 839 mg (7.92 mmol) of Na_2CO_3, 9 mL of toluene, 3 mL of EtOH and 3 mL of H_2O. The resulting mixture was degassed by bubbling Ar for 10 minutes, and $[Pd(PPh_3)_4]$ (45 mg, 0.039 mmol, 2.5 mol%) was added to the mixture. A second degassing was performed (10 minutes), the Schlenk tube was sealed, and the mixture was stirred at 95 °C for 20 h. The reaction mixture was then filtered through a Celite pad and washed with AcOEt. The solvents were removed *in vacuo*, affording a brown oil. The product was purified by silica gel column chromatography (CH_2Cl_2/AcOEt 7:3), providing an off-white powder. Further purification was performed by dissolving the compound in a minimum amount of CH_2Cl_2, and the product was precipitated by adding *n*-heptane. The precipitate was filtered, washed with n-heptane, and dried *in vacuo* to afford 430 mg (1.26 mmol) of **2** as a white powder (mixture of atropisomers). Yield: 80%.

Mp: 117–123 °C. **^{1}H NMR** (600 MHz, CD_2Cl_2) δ (ppm): 7.44 (s, 2H, H_a), 7.22 – 7.17 (m, 2H, H_e), 7.17 – 7.11 (m, 2H, H_d), 6.90 (s, 2H, H_b), 6.84 – 6.79 (m, 4H, H_c, H_f), 3.83 (bs, 8H, NH_2). **^{13}C NMR** (151 MHz, CD_2Cl_2) δ (ppm): 145.20, 145.19, 143.86, 143.82, 136.41, 136.40, 131.6, 131.5, 130.34, 130.33, 129.26, 125.09, 125.05, 124.9, 124.8, 123.24, 123.19, 118.92, 118.87, 115.8,

106.8, 106.7. **FTIR** (ATR) ν (cm^{-1}): 3423, 3342, 1628, 1609, 1491, 1472, 1440, 1294, 1261, 1245, 1187, 1165, 989, 913, 871, 757, 730, 705, 638, 585, 554, 528, 493, 472, 434. **HRMS** (ESI): *m/z* calcd for $[C_{22}H_{21}N_4]^+$: 341.1761 $[M+H]^+$; found: 341.1757.

Molecule 3

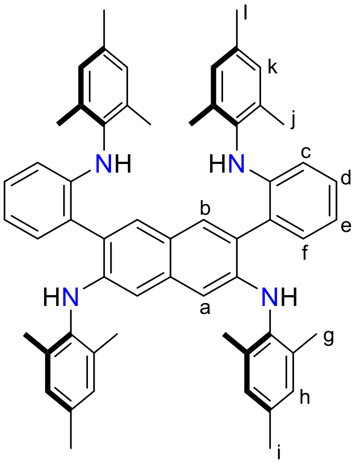

In the glove box, 80 mg (0.24 mmol) of **2**, 2 mg (0.002 mmol, 1 mol%) of $[Pd_2(dba)_3]$, 4 mg (0.006 mmol, 3 mol%) of racemic BINAP and 136 mg (1.42 mmol) of *t*BuONa were loaded in a 10 mL microwave vial. 0.16 mL (1.0 mmol) of mesityl bromide and 2 mL of toluene were added, the vial was sealed, and the mixture was stirred at 110 °C for 72 h. The resulting viscous liquid was diluted with 100 mL of AcOEt and washed with H_2O (2 x 40 mL) and brine (1 x 40 mL). The organic layer was dried over $MgSO_4$, and the solvents were removed in *vacuo* to afford a brown solid. The crude product was purified by silica gel column chromatography (*n*-heptane/CH_2Cl_2 8:2), affording 115 mg (0.141 mmol) of **3** as a white powder (mixture of atropisomers). Yield: 60%.

Mp > 250 °C. **^{1}H NMR** (600 MHz, CD_2Cl_2) δ (ppm): 7.68 – 7.62 (m, 2H, H_b), 7.33 – 7.25 (m, 2H, H_f), 7.16 – 7.09 (m, 2H, H_d), 6.93 – 6.79 (m, 8H, H_h, H_k), 6.85 – 6.79 (m, 2H, H_e), 6.26 – 6.20 (m, 2H, H_c), 6.09 – 6.05 (m, 2H, H_a), 5.34 (bs, 4H, NH, partial overlap with CD_2Cl_2), 2.30 – 2.24 (m, 12H, H_i, H_l), 2.17 – 2.11 (m, 24H, H_g, H_j). **^{13}C NMR** (151 MHz, CD_2Cl_2) δ (ppm): 145.7, 145.6, 144.06, 144.03, 137.05, 137.04, 136.14, 136.06, 135.98, 135.81, 135.76, 135.75, 135.69, 131.4, 131.3, 130.3, 130.2, 129.54, 129.52, 129.46, 123.9, 123.8, 123.7, 123.6, 122.1, 122.0, 117.9, 117.8, 111.8, 103.2, 103.1, 21.06, 21.05, 21.03, 18.42, 18.37, 18.3. **FTIR** (ATR) ν (cm^{-1}): 3401, 3375, 2915, 2855, 1628, 1596, 1573, 1490, 1448, 1379, 1307, 1296, 1274, 1241, 1218, 1160,

1036, 1012, 913, 856, 759, 625, 497, 463, 449, 421, 407. **HRMS** (MALDI): *m/z* calcd for $[C_{58}H_{60}N_4]^+$: 812.4812 $[M]^+$; found: 812.4802.

Molecule $\mathbf{1_{NBNBN}}$

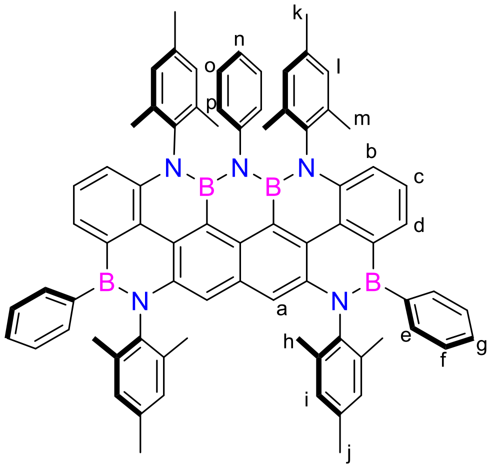

In the glove box, a 10 mL Schlenk tube was filled with 50 mg (0.061 mmol) of **3** and 1 mL of TCB, 0.1 mL (1 mmol) of BBr_3 was added and the mixture was kept stirring at 240 °C for 18 h. The volatiles were removed *in vacuo*, and the vessel was transferred into the glove box, where 1 mL of THF and 0.72 mL of a 1 M solution of PhMgBr (0.72 mmol) in THF were added. The Schlenk tube was sealed, and the mixture was stirred at RT for 18 h. The volatiles were removed *in vacuo,* affording a red solid. The vessel was transferred into the glove box, where 2 mL of TCB and 0.39 mL (4.3 mmol, 70 eq) of $PhNH_2$ were added. The vessel was then sealed, and the mixture was stirred at 230 °C for 48 h. The volatiles were removed *in vacuo*, affording a brown solid. The crude product was purified by silica gel column chromatography (n-heptane/CH_2Cl_2 from 10:0 to 8:2), affording 56 mg of a yellow solid. The solid was then purified by recycling preparative HPLC (CH_2Cl_2/CH_3CN 9:1), affording 8 mg (0.008 mmol) and 32 mg (0.031 mmol) of $\mathbf{1_{NBNBN}}$ and $\mathbf{1_{NBOBN}}$, respectively. Yield: 13% ($\mathbf{1_{NBNBN}}$), 51% ($\mathbf{1_{NBOBN}}$).

Mp > 250 °C (dec). **^{1}H NMR** (400 MHz, CD_2Cl_2) δ 7.52 (d, *J* = 5.7 Hz, 4H, H_d, H_a), 7.40 – 7.28 (m, 6H, H_e, H_c), 7.23 (d, *J* = 5.4 Hz, 6H, H_f, H_g), 6.98 (d, *J* = 5.1 Hz, 4H, H_i), 6.74 (d, *J* = 7.1 Hz, 2H, H_b), 6.68 (m, 6H, H_l, H_n), 6.30 (t, *J* = 6.7 Hz, 2H, H_o), 6.11 (m, 2H, H_p), 2.34 (s, 6H, H_j), 2.33 (s, 6H, H_k), 2.06 (s, 12H, H_h), 1.69 (s, 12H, H_m). **^{13}C NMR** (176 MHz, CD_2Cl_2) δ (ppm): 143.31, 141.16, 140.23, 139.98, 138.45, 137.27, 137.00, 136.46, 133.65, 132.64, 130.73, 130.36, 130.15, 129.90, 129.87, 129.77, 129.30, 127.65, 127.27, 126.39, 126.20, 123.48, 118.39, 114.81, 21.44, 21.24, 18.69, 18.59 (three signals missing due to ^{11}B-induced quadrupolar relaxation, 1 signal missing probably due to superimposition of 2 different peaks). **^{11}B NMR** (193 MHz, CD_2Cl_2) δ (ppm): 43.28, 29.93. **FTIR** (ATR) ν (cm^{-1}): 2924, 2853, 1730, 1604, 1581, 1483, 1434, 1379, 1347, 1288, 1257, 1102, 1032, 889, 853, 822, 800, 765, 745, 704. **HRMS** (MALDI): *m/z* calcd for $[C_{76}H_{67}N_5B_4]^+$: 1093.5802 $[M]^+$; found: 1093.5813. **Crystal** suitable for X-ray diffraction was obtained by vapor diffusion from toluene/CH_3CN (CCDC #2279843 – see Table S2).

Molecule 2_{NBNBN}

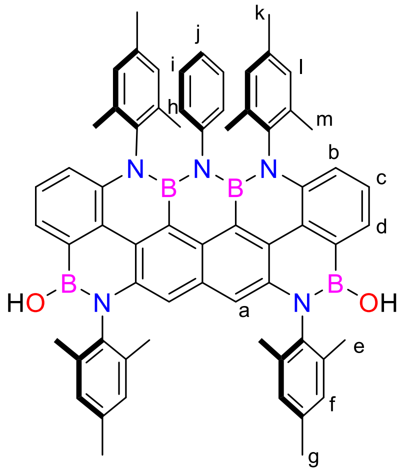

In the glove box, a 10 mL Schlenk tube was filled with 50 mg (0.061 mmol) of **3** and 1 mL of TCB. 0.1 mL (1 mmol) of BBr_3 was added, the Schlenk tube was sealed, and the mixture was stirred at 240 °C for 18 h. The volatiles were removed *in vacuo*, and the vessel was transferred into the glove box, where 1 mL of toluene and 0.04 mL (0.4 mmol) of $PhNH_2$ were added. The vessel was sealed, and the mixture was stirred at 110 °C for 72 h. The brown mixture was allowed to reach rt, and 0.1 mL of H_2O was added. The resulting heterogeneous mixture was stirred at RT for 1 h. After removal of the volatiles *in vacuo*, the resulting brown oil was purified by aluminum oxide column chromatography (n-heptane/CH_2Cl_2 from 6:4 to 4:6). The compound was further purified by recycling preparative HPLC (CH_2Cl_2) affording 42 mg (0.043 mmol) of $\mathbf{2_{NBNBN}}$ as a yellow solid. Yield: 70%.

Mp > 250 °C (dec). **^{1}H NMR** (400 MHz, CD_2Cl_2) δ 7.91 (dd, *J* = 6.9, 1.0, 2H, H_d), 7.36 (dd, *J* = 8.5, 6.9, 2H, H_c), 7.22 (s, 4H, H_f), 7.17 (s, 2H, H_a), 6.68 – 6.63 (m, 7H, H_b, H_l, H_j), 6.29 (t, *J* = 7.7, 2H, H_i), 6.05 (dd, *J* = 8.1, 1.0, 2H, H_h), 4.07 (s, 2H, B-OH), 2.48 (s, 6H, H_g), 2.32 (s, 6H, H_k), 2.09 (s, 12H, H_e), 1.66 (s, 12H, H_m). **^{13}C NMR** (151 MHz, CD_2Cl_2) δ 143.31, 141.24, 139.93, 138.92, 138.38, 137.40, 136.91, 136.47, 135.46, 131.34, 130.83, 130.08, 129.76, 128.42, 127.68, 127.55, 127.28, 126.15, 124.41, 123.41, 118.30, 112.45, 21.55, 21.22, 18.64, 18.19. **^{11}B NMR** (193 MHz, CD_2Cl_2) δ (ppm): 29.42. **FTIR** (ATR) ν (cm^{-1}): 3603, 3550, 3486, 2916, 2855, 1608, 1579, 1480, 1437, 1410, 1387, 1359, 1314, 1303, 1278, 1247, 1235, 1154, 1124, 1010, 882, 862, 845, 821, 802, 760, 737, 703, 681, 671, 647, 604, 578, 554, 527, 508, 491, 461, 412. **HRMS** (MALDI): *m/z* calcd for $[C_{64}H_{59}N_5B_4O_2]^+$: 973.5070 $[M]^+$; found: 973.5072.

Molecule 1_{NBOBN}

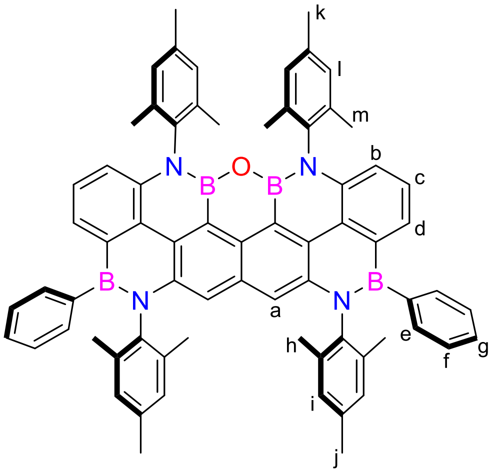

In the glove box, a 10 mL Schlenk tube was filled with 50 mg (0.061 mmol) of **3** and 1 mL of TCB. 0.1 mL (1 mmol) of BBr_3 was added, and the mixture was stirred at 240 °C for 18 h. The volatiles were removed *in vacuo*, and the vessel was transferred into the glove box, where 1 mL of THF and 0.72 mL of a 1M solution of PhMgBr (0.72 mmol) in THF were added. The Schlenk tube was sealed, and the mixture was stirred at RT for 18 h. The resulting red suspension was then treated with 0.1 mL of H_2O and stirred at 70 °C for 1 h. The volatiles were removed *in vacuo,* affording a brown solid. The crude product was purified by silica gel column chromatography (n-heptane/CH_2Cl_2 from 10:0 to 8:2), affording 60 mg of a yellow solid, which was further purified by recycling preparative HPLC (CH_2Cl_2), affording 45 mg (0.044 mmol) of $\mathbf{1}_{\mathbf{NBOBN}}$ as a yellow solid. Yield: 72%.

Mp > 250 °C (dec). **^{1}H NMR** (700 MHz, CD_2Cl_2) δ (ppm): 7.61 (dd, *J* = 7.1, 1.1, 2H, H_d), 7.51 (dd, *J* = 8.1, 7.1, 2H, H_c), 7.44 – 7.36 (m, 6H, H_a, H_e), 7.29 – 7.20 (m, 8H, H_b, H_f, H_g), 6.98 (s, 4H, H_i), 6.94 (s, 4H, H_l), 2.41 (s, 6H, H_k), 2.34 (s, 6H, H_j), 2.04 (s, 12H, H_h), 1.88 (s, 12H, H_m). **^{13}C NMR** (176 MHz, CD_2Cl_2) δ (ppm): 140.59, 139.81, 139.18, 137.17, 136.70, 136.13, 135.64, 132.55, 130.29, 130.11, 129.81, 129.77, 129.75, 129.03, 127.63, 127.43, 127.18, 126.27, 118.25, 113.40, 21.27, 21.22, 18.38, 17.92 (three signals missing due to ^{11}B-induced quadrupolar relaxation, 1 signal missing probably due to superimposition of 2 different peaks). **^{11}B NMR** (193 MHz, CD_2Cl_2) δ (ppm): 404.51, 29.24. **FTIR** (ATR) ν (cm^{-1}): 2919, 2853, 1594, 1569, 1482, 1440, 1385, 1345, 1322, 1291, 1265, 1236, 1209, 1059, 1029, 890, 853, 824, 802, 768, 749, 701, 682, 545. **HRMS** (MALDI): *m/z* calcd for $[C_{70}H_{62}N_4B_4O]^+$: 1018.5327 $[M]^+$; found: 1018.5356. **Crystal** suitable for X-ray diffraction was obtained by vapor diffusion from toluene/CH_3CN (CCDC #2268752 – see Table S3).

Molecule 2_{NBOBN}

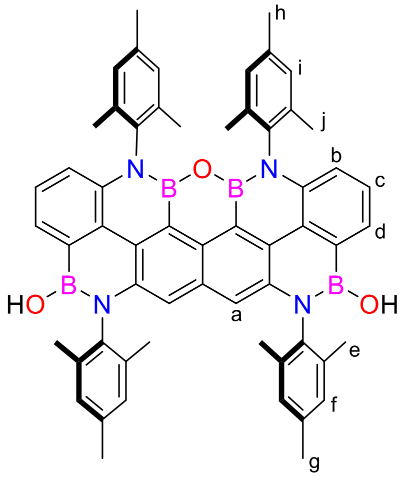

In the glove box, a 10 mL Schlenk tube was filled with 50 mg (0.061 mmol) of **3** and 1 mL of TCB. 0.1 mL (1 mmol) of BBr_3 was added and the mixture was stirred at 240 °C for 18 h. The brown mixture was allowed to reach RT, 0.1 mL of H_2O was added, and the resulting heterogeneous mixture was stirred at 70 °C for 1 h. After removal of the volatiles *in vacuo*, the resulting brown oil was purified by aluminum oxide column chromatography (n-heptane/CH_2Cl_2 from 6:4 to 4:6), affording 49 mg (0.055 mmol) of $\mathbf{2_{NBOBN}}$ as a yellow solid. Yield: 90%.

Mp > 250 °C (dec). **^{1}H NMR** (400 MHz, CD_2Cl_2) δ (ppm): 7.96 (dd, *J* = 6.9, 1.1, 2H, H_d), 7.55 (dd, *J* = 8.2, 6.9, 2H, H_c), 7.20 – 7.16 (m, 6H, H_f, H_b), 7.04 (s, 2H, H_a), 6.91 (s, 4H, H_i), 4.19 (s, 2H, B-OH), 2.46 (s, 6H, H_g), 2.40 (s, 6H, H_h), 2.06 (s, 12H, H_e), 1.84 (s, 12H, H_j). **^{13}C NMR** (151 MHz, CD_2Cl_2) δ (ppm): 140.66, 139.64, 138.26, 137.14, 136.59, 136.09, 135.63, 135.13, 130.87, 130.64, 128.96, 128.28, 128.18, 127.45, 127.41, 124.27, 118.09, 110.94, 21.33, 21.20, 17.97, 17.87 (two signals missing due to ^{11}B-induced quadrupolar relaxation). **^{11}B NMR** (193 MHz, CD_2Cl_2) δ (ppm): 29.17. **FTIR** (ATR) ν (cm^{-1}): 3589, 2917, 1603, 1587, 1483, 1441, 1394, 1364, 1328, 1312, 1293, 1269, 1252, 1216, 1183, 1124, 1011, 880, 839, 822, 797, 764, 738, 703, 683, 671, 648, 604, 556, 543, 532, 511. **HRMS** (LD): *m/z* calcd for $[C_{58}H_{54}N_4B_4O_3]^+$: 898.4595 $[M]^+$; found: 898.4585.

Molecule 4

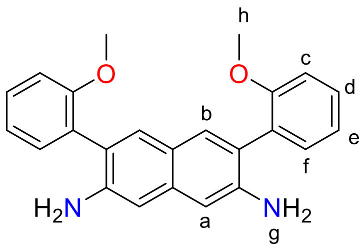

In a 250 mL round bottom flask were subsequently added 1.50 g (4.75 mmol) of **1**, 2.67 g (11.4 mmol) of 2-methoxyphenylboronic acid pinacol ester, 2.52 g (23.75 mmol) of Na_2CO_3, 30 mL of toluene, 10 mL of EtOH and 10 mL of H_2O. The resulting mixture was degassed by bubbling Ar for 10 minutes, and 274 mg of [Pd(PPh_3)$_4$] (0.238 mmol, 5 mol%) were added. A second degassing was performed (10 minutes), the round bottom flask was equipped with a condenser, and the mixture stirred at 95 °C for 20 h under an Ar atmosphere. The reaction mixture was then extracted with 200 mL of AcOEt and washed with H_2O (2 x 50 mL) and brine (1 x 50 mL). The organic layer was dried over $MgSO_4$, and the solvent was removed *in vacuo*. The product was purified by silica gel column chromatography (CH_2Cl_2/AcOEt 95:5), providing a light-brown powder. The powder was dissolved in a minimum amount of CH_2Cl_2, and the product was precipitated by adding *n*-heptane. The precipitate was filtered, washed with n-heptane, and dried *in vacuo* to afford 550 mg (1.48 mmol) of **4** as a yellow powder. Yield: 31%.

Mp: 93–97 °C (dec). **^{1}H NMR** (400 MHz, $CDCl_3$) δ (ppm): 7.41-7.37 (m, 4H, H_b, H_d), 7.29 – 7.27 (dd, *J* = 7.4, 1.7 Hz, 2H, H_f), 7.08 – 7.03 (m, 4H, H_e, H_c), 6.84 (s, 2H, H_a), 3.81 (s, 10 H, H_h, H_g). **^{13}C NMR** (151 MHz, CD_2Cl_2) δ (ppm): 157.20, 143.46, 135.68, 132.26, 130.09, 129.17, 128.62, 125.29, 123.03, 121.33, 111.35, 107.23, 55.94. **FTIR** (ATR) ν (cm^{-1}): 3444, 3356, 3002, 2932, 2832, 1630, 1597, 1578, 1488, 1455, 1429, 1292, 1254, 1236, 1177, 1115, 1049, 1021, 988, 907, 862, 787, 751, 697, 616, 568, 532, 514, 471, 431. **HRMS** (ESI): *m/z* calcd for $[C_{24}H_{23}N_2O_2]^+$: 371.1754 $[M+H]^+$; found: 371.1739.

Molecule 5

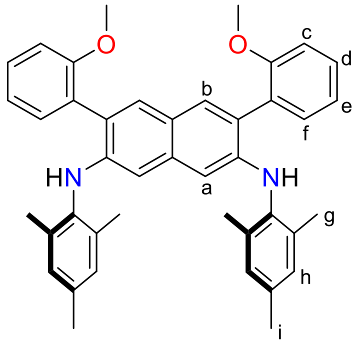

In the glove box, 220 mg (0.594 mmol) of **4**, 14 mg (0.015 mmol, 2.5 mol%) of [$Pd_2(dba)_3$], 28 mg (0.045 mmol) of racemic BINAP and 228 mg (2.37 mmol) of *ᵗ*BuONa were loaded in a 20 mL microwave vial. 0.36 mL (2.38 mmol) of mesityl bromide and 6 mL of toluene were added, the vial was sealed, and the mixture was stirred at 110 °C for 24 h. The resulting viscous liquid was diluted with 150 mL of AcOEt and washed with H_2O (2 x 50 mL) and brine (1 x 50 mL). The organic layer was dried over $MgSO_4$, and the solvents were removed *in vacuo* to afford a brown solid. The crude product was purified by silica gel column chromatography (n-heptane/CH_2Cl_2 7:3) followed by recrystallization from n-heptane/CH_2Cl_2, affording 319 mg (0.526 mmol) of **5** as white crystals (mixture of atropisomers). Yield = 89%.

Mp: 192-195 °C. **^{1}H NMR** (400 MHz, CD_2Cl_2) δ (ppm) 7.46 – 7.31 (m, 6H, H_b, H_d, H_f), 7.13 – 7.01 (m, 4H, H_e H_c), 6.89 (s, 4H, H_h), 5.97 (s, 2H, H_a), 5.25 (s, 2H, H_k), 3.88 – 3.79 (m, 6H, H_j), 2.26 (s, 6H, H_i), 2.10 (s, 12H, H_g). **^{13}C NMR** (101 MHz, CD_2Cl_2) δ (ppm): 157.76, 143.52, 136.31, 136.03, 135.89, 135.31, 132.42, 129.87, 129.54, 129.45, 129.03, 125.59, 122.04, 121.52, 111.40, 111.22, 103.71, 55.91, 21.02, 18.12. **FTIR** (ATR) ν (cm^{-1}): 3400, 3000, 2940, 2914, 2855, 2834, 1629, 1597, 1579, 1485, 1452, 1431, 1375, 1306, 1290, 1259, 1238, 1224, 1164, 1113, 1050, 1025, 991, 936, 909, 850, 797, 786, 752, 735, 699, 604, 586, 561, 491, 473, 436, 411. **HRMS** (LD): *m/z* calcd for $[C_{42}H_{42}N_2O_2]^+$: 606.3241 $[M]^+$; found: 606.3238.

Molecule 1_{OBOBO}

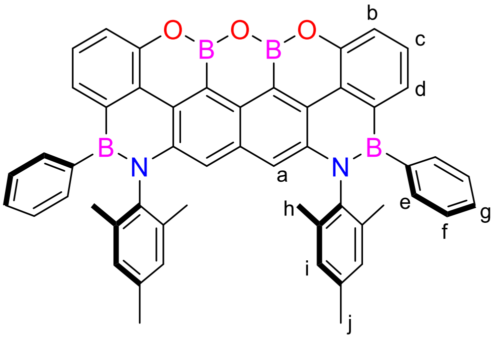

In the glove box, a 10 mL Schlenk tube was filled with 50 mg (0.082 mmol) of **5** and 1.4 mL of TCB. 0.14 mL (1.4 mmol) of BBr_3 was added, and the mixture was stirred at 240 °C for 18 h. The volatiles were removed *in vacuo*, and the vessel was transferred into the glove box, where 1.4 mL of THF and 0.97 mL of a 1 M solution of PhMgBr (0.97 mmol) in THF were added. The Schlenk tube was sealed, and the mixture was stirred at RT for 18 h. The resulting red suspension was then treated with 0.1 mL of H_2O and stirred at 70 °C for 1 h. The volatiles were removed *in vacuo,* affording a brown solid. The crude product was purified by silica gel column chromatography (n-heptane/CH_2Cl_2 from 8:2 to 1:1), affording 43 mg of a yellow solid, which was further purified by recycling preparative HPLC (CH_2Cl_2), affording 36 mg (0.046 mmol) of **1_{OBOBO}** as a yellow solid. Yield: 56%.

Mp > 250 °C (dec). **^{1}H NMR** (400 MHz, CD_2Cl_2) δ (ppm): 7.96 (dd, *J* = 7.2, 2.0 Hz, 2H, H_c), 7.77 – 7.70 (m, 4H, H_b, H_d), 7.40 – 7.34 (m, 2H, H_a, H_e), 7.30 – 7.25 (m, 6H, H_f, H_g), 6.96 (s, 4H, H_i), 2.33 (s, 6H, H_j), 1.99 (s, 12H, H_h). **^{13}C NMR** ^{13}C NMR (151 MHz, CD_2Cl_2) δ 151.53, 139.34, 138.94, 137.58, 135.64, 132.83, 132.50, 131.39, 130.69, 130.47, 130.00, 128.74, 128.14, 127.46, 126.79, 122.28, 115.44, 21.39, 18.44 (three signals missing due to ^{11}B-induced quadrupolar relaxation). **^{11}B NMR** (193 MHz, CD_2Cl_2) δ (ppm): 40.64, 29.29. **FTIR** (ATR) ν (cm^{-1}): 2920, 2851, 1671, 1592, 1461, 1434, 1378, 1336, 1262, 1204, 1028, 887, 819, 758, 702, 674, 489. **HRMS** (LD): *m/z* calcd for $[C_{52}H_{40}N_2B_4O_3]^+$: 784.3435 $[M]^+$; found: 784.3436. **Crystal** suitable for X-ray diffraction was obtained by slow evaporation from toluene (CCDC #2331294 – see Table S4).

Molecule 2_{OBOBO}

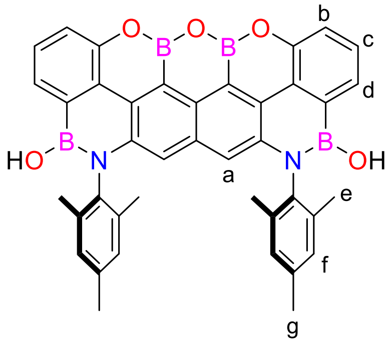

In the glove box, a 10 mL Schlenk tube was filled with 20 mg (0.061 mmol) of **5** and 1 mL of TCB. 0.05 mL (0.5 mmol) of BBr_3 were added, the Schlenk tube was sealed, and the mixture was stirred at 240 °C for 18 h. The volatiles were removed in *vacuo*. The resulting red suspension was then treated with 0.1 mL of H_2O and stirred at 70 °C for 2 h. The volatiles were removed *in vacuo,* and the resulting brownish powder was further purified by column chromatography (n-heptane/CH_2Cl_2, 1:1), affording 8 mg of $\mathbf{2_{OBOBO}}$ as a pale-yellow solid. Yield: 36%

Mp > 250 °C (dec). **^{1}H NMR** (600 MHz, CD_2Cl_2) δ 8.06 (dd, *J* = 7.1, 1.2 Hz, 2H, H_b), 7.90 (dd, *J* = 8.0, 1.2 Hz, 2H, H_d), 7.76 (t, *J* = 7.6 Hz, 2H, H_c), 7.17 (s, 4H, H_a), 7.02 (s, 2H, H_f), 4.27 (s, 2H, B-OH), 2.43 (s, 6H, H_g), 2.02 (s, 12H, H_e). **^{13}C NMR** (151 MHz, CD_2Cl_2) δ 151.42, 145.89, 139.40, 138.49, 136.99, 134.51, 131.88, 130.70, 128.89, 128.81, 128.59, 127.86, 127.00, 122.03, 112.66, 21.29, 17.87 (three signals missing due to ^{11}B-induced quadrupolar relaxation). **^{11}B NMR** (193 MHz, CD_2Cl_2) δ (ppm): 29.74. **FTIR** (ATR) ν (cm^{-1}): 3600, 2919, 2850, 1671, 1616, 1592, 1478, 1434, 1392, 1339, 1293, 1259, 1205, 1125, 1034, 879, 853, 818, 797, 756, 693, 674, 646, 603, 555, 528, 504, 488. (LD): *m/z* calcd for $[C_{40}H_{32}B_4N_2O_5]^+$: 664.2702 $[M]^+$; found: 664.2684.

Molecule 2_{OBNBO}

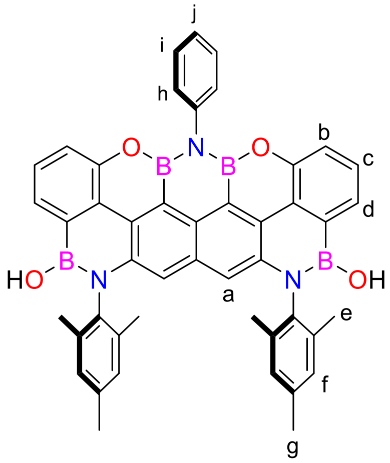

In the glove box, a 10 mL Schlenk tube was filled with 30 mg (0.049 mmol) of **5** and 0.3 mL of DCB. 0.08 mL (0.08 mmol) of BBr_3 were added, the Schlenk tube was sealed, and the mixture was stirred at 200 °C for 18 h. The volatiles were removed *in vacuo*. The vessel was transferred into the glove box, where 0.5 mL of toluene and 0.1 mL (1.1 mmol) of aniline were subsequently added. The Schlenk tube was sealed, and the mixture was stirred at 120 °C for 18 h. The resulting brownish powder was purified by column chromatography in heptane / dichloromethane (1:1), affording 6 mg (0.008 mmol) of $\mathbf{2_{OBNBO}}$ as a yellow solid. Yield: 16%.

Mp > 250 °C (dec). **^{1}H NMR** (400 MHz, CD_2Cl_2) δ 8.02 (t, *J* = 4.1 Hz, 2H, H_c), 7.68 – 7.61 (m, 8H, H_b, H_d, H_h, H_i), 7.47 (t, *J* = 6.9 Hz, 1H, H_j), 7.18 (s, 4H, H_f), 7.06 (s, 2H, H_a), 4.20 (s, 2H, B-OH), 2.45 (s, 6H, H_g), 2.03 (s, 12H, H_e). **^{13}C NMR** (151 MHz, CD_2Cl_2) δ 151.42, 141.69, 139.07, 138.41, 137.05, 134.72, 132.07, 130.69, 129.19, 128.40, 128.28, 127.89, 127.52, 127.30, 126.40, 125.81, 121.56, 112.70, 21.32, 17.90. **^{11}B NMR** (193 MHz, CD_2Cl_2) δ (ppm): 28.11. **FTIR** (ATR) ν (cm^{-1}): 3564, 2919, 2850, 2361, 1616, 1481, 1437, 1397, 1340, 1294, 1259, 1239, 1185, 1123, 1038, 981, 882, 854, 817, 797, 758, 733, 699, 673, 608, 547, 526, 488.

3. NMR spectra

Molecule 1

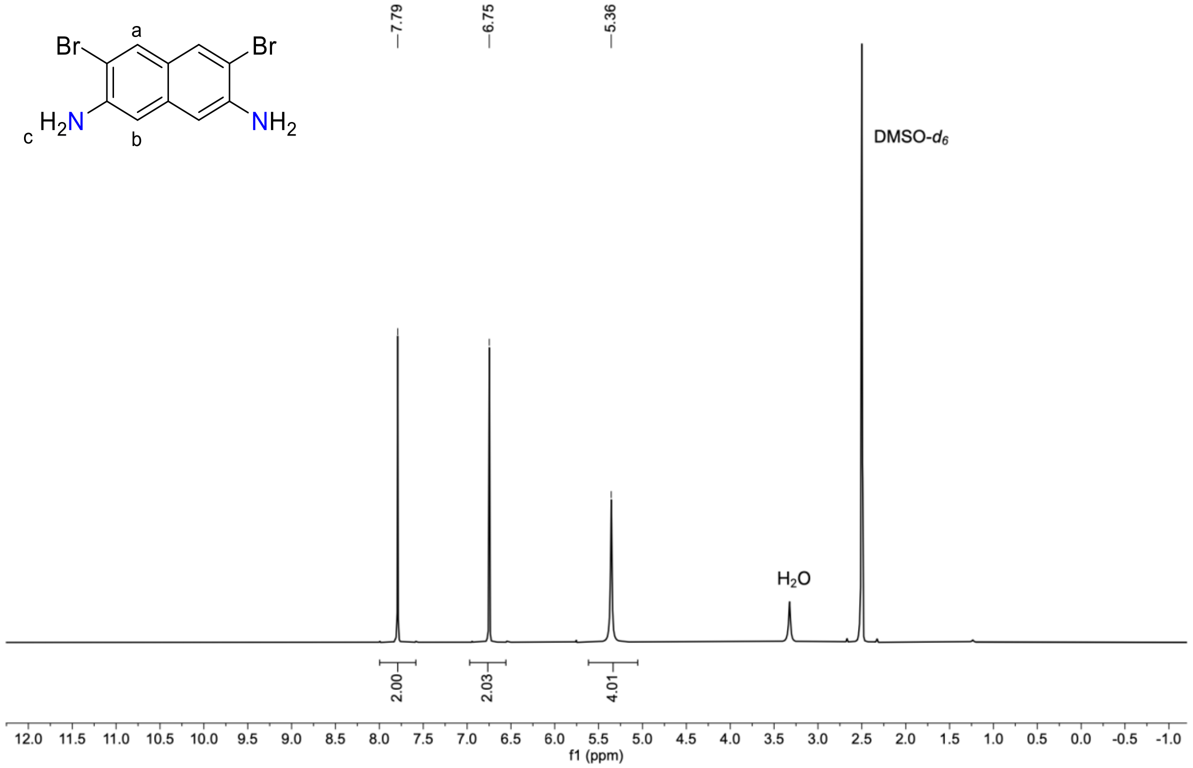

Figure S1. ^{1}H NMR (400 MHz, DMSO-d_6) of molecule **1**.

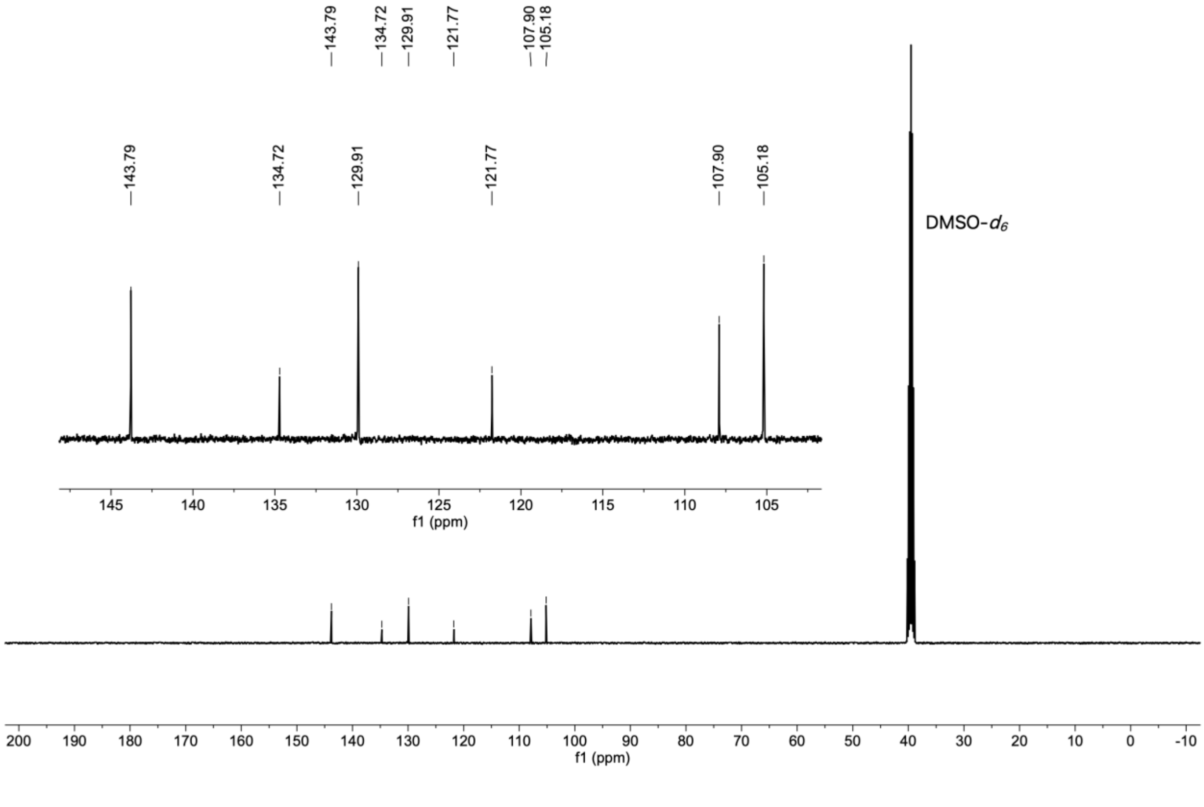

Figure S2. ^{13}C NMR (101 MHz, DMSO-d_6) of molecule **1**.

Molecule 2

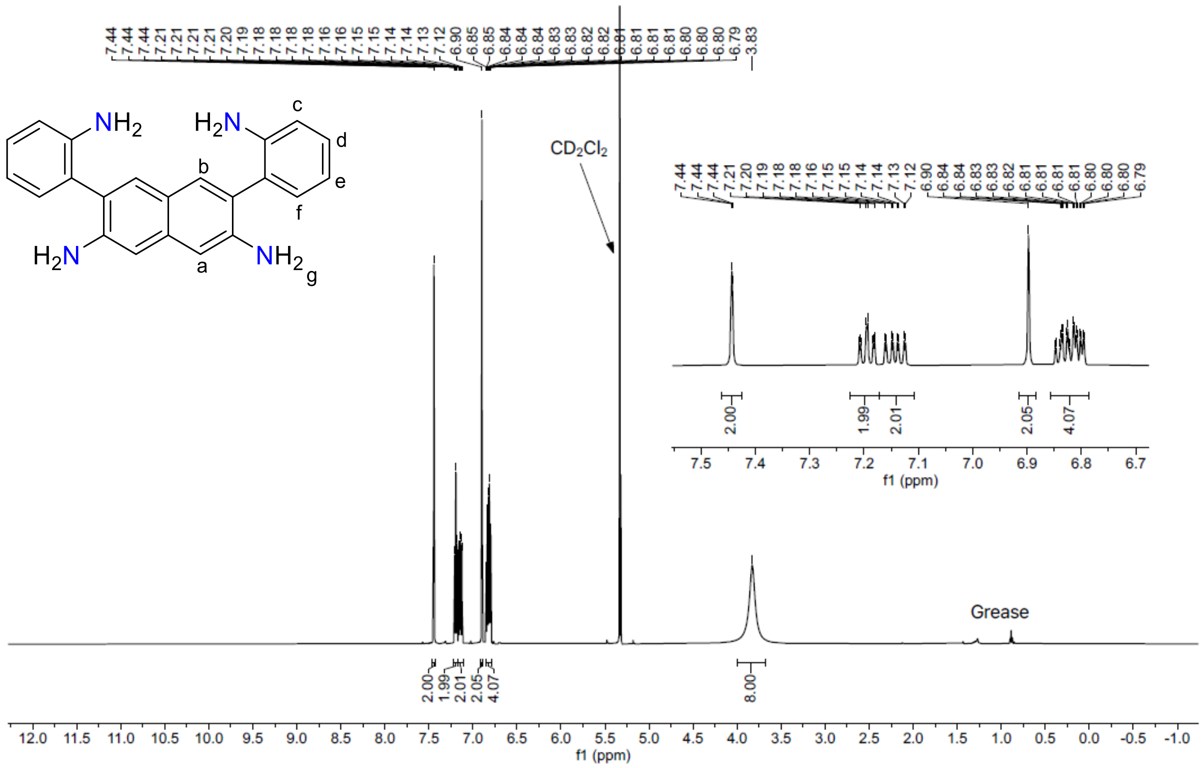

Figure S3. ^{1}H NMR (600 MHz, CD_2Cl_2) of molecule **2**.

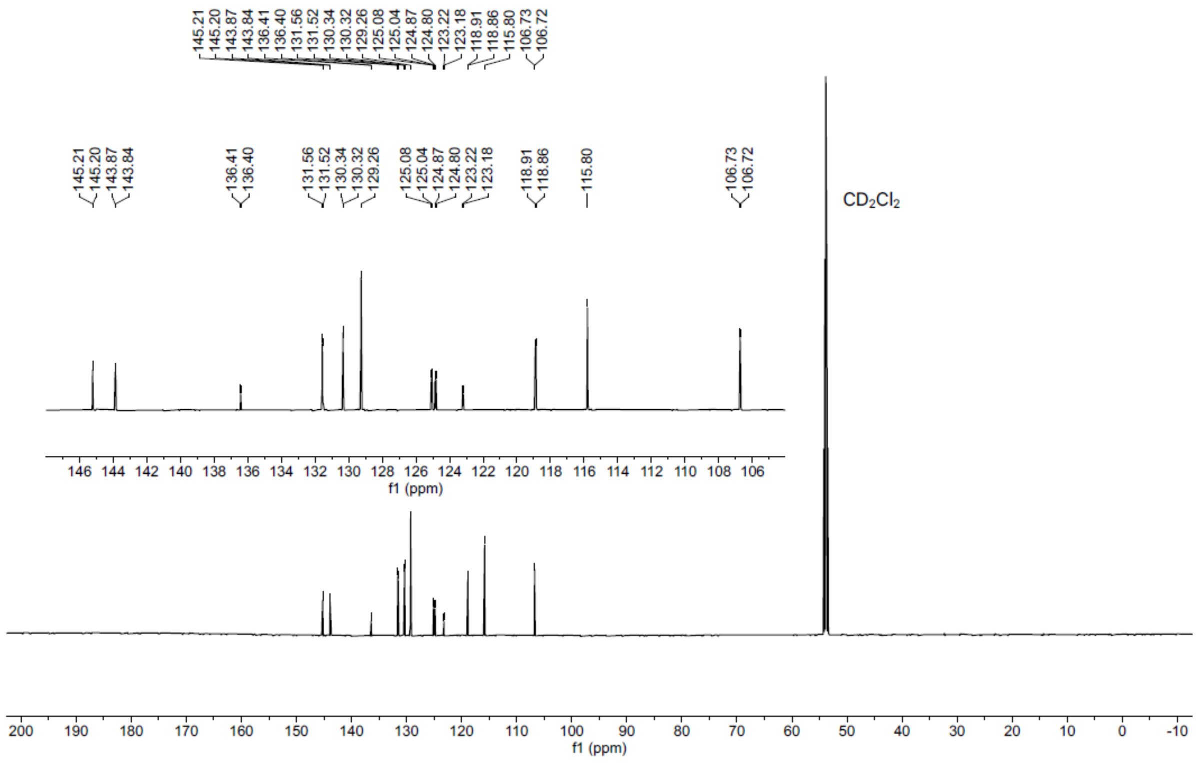

Figure S4. ^{13}C NMR (151 MHz, CD_2Cl_2) of molecule **2**.

Molecule 3

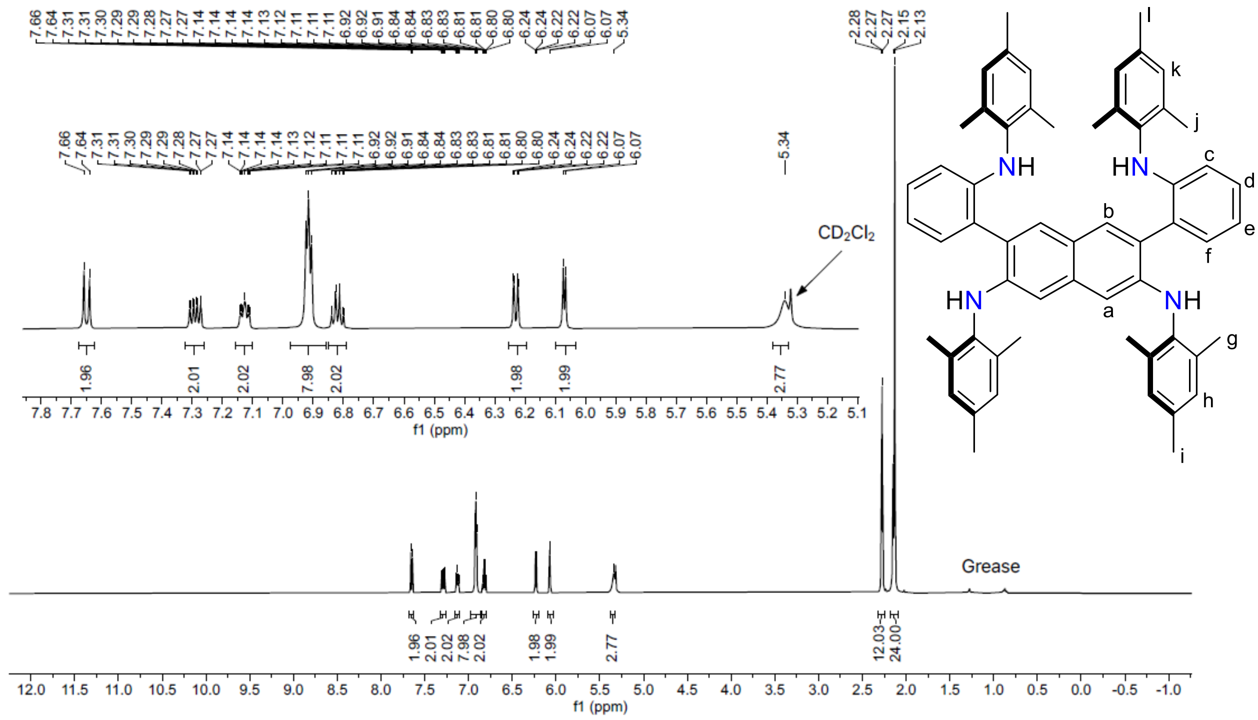

Figure S5. ^{1}H NMR (600 MHz, CD_2Cl_2) of molecule **3**.

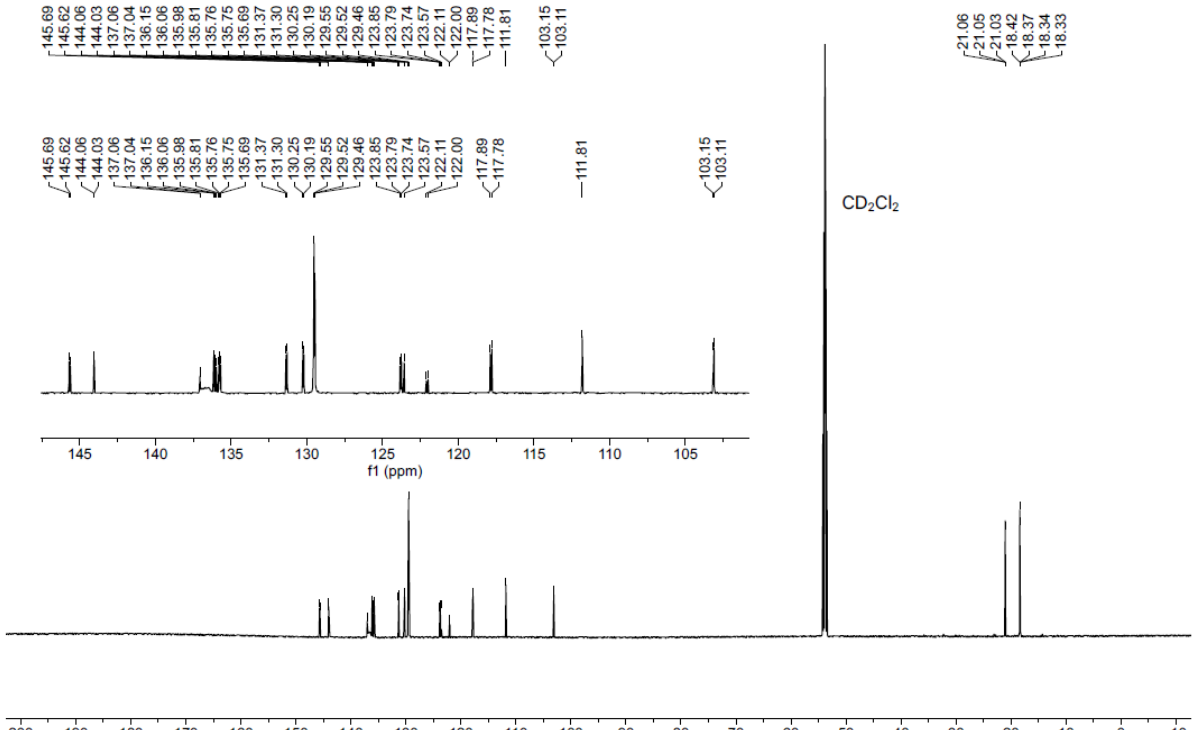

Figure S6. ^{13}C NMR (151 MHz, CD_2Cl_2) of molecule **3**.

Molecule 1_{NBNBN}

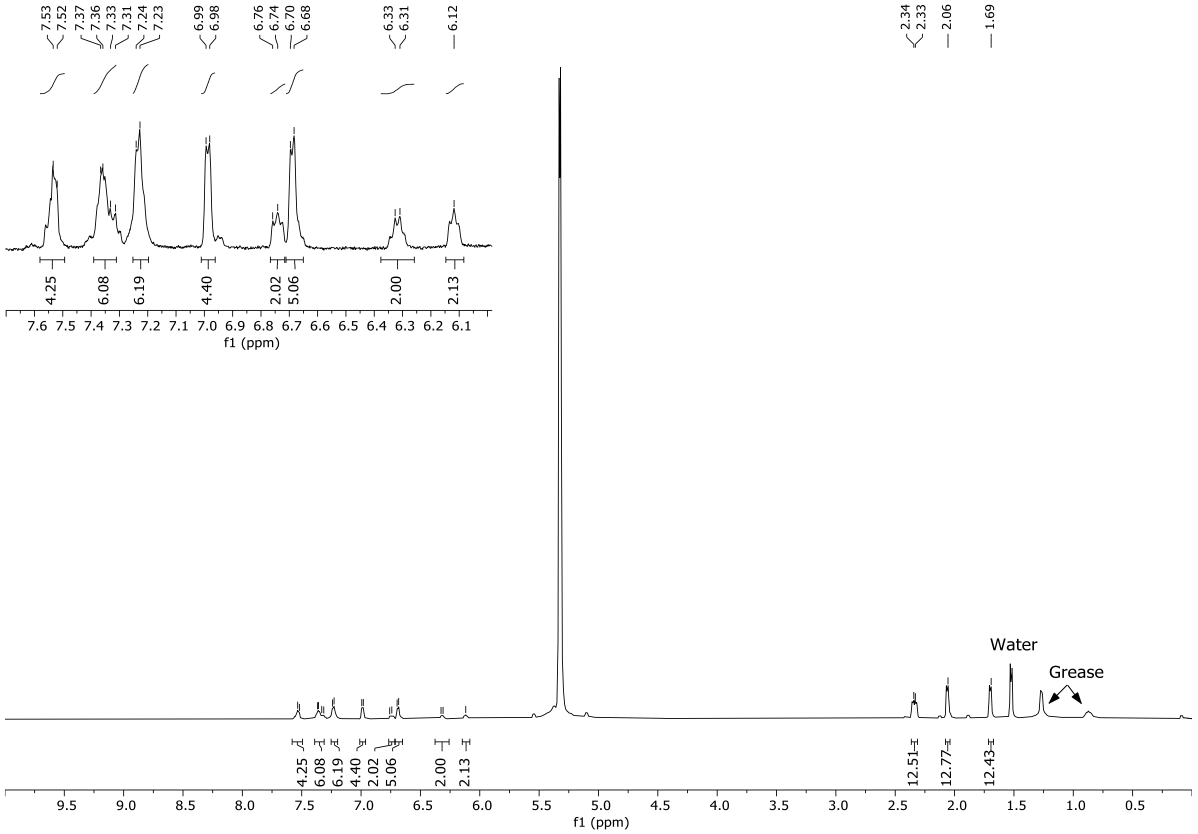

Figure S7. ^{1}H NMR (700 MHz, CD_2Cl_2) of molecule $\mathbf{1_{NBNBN}}$.

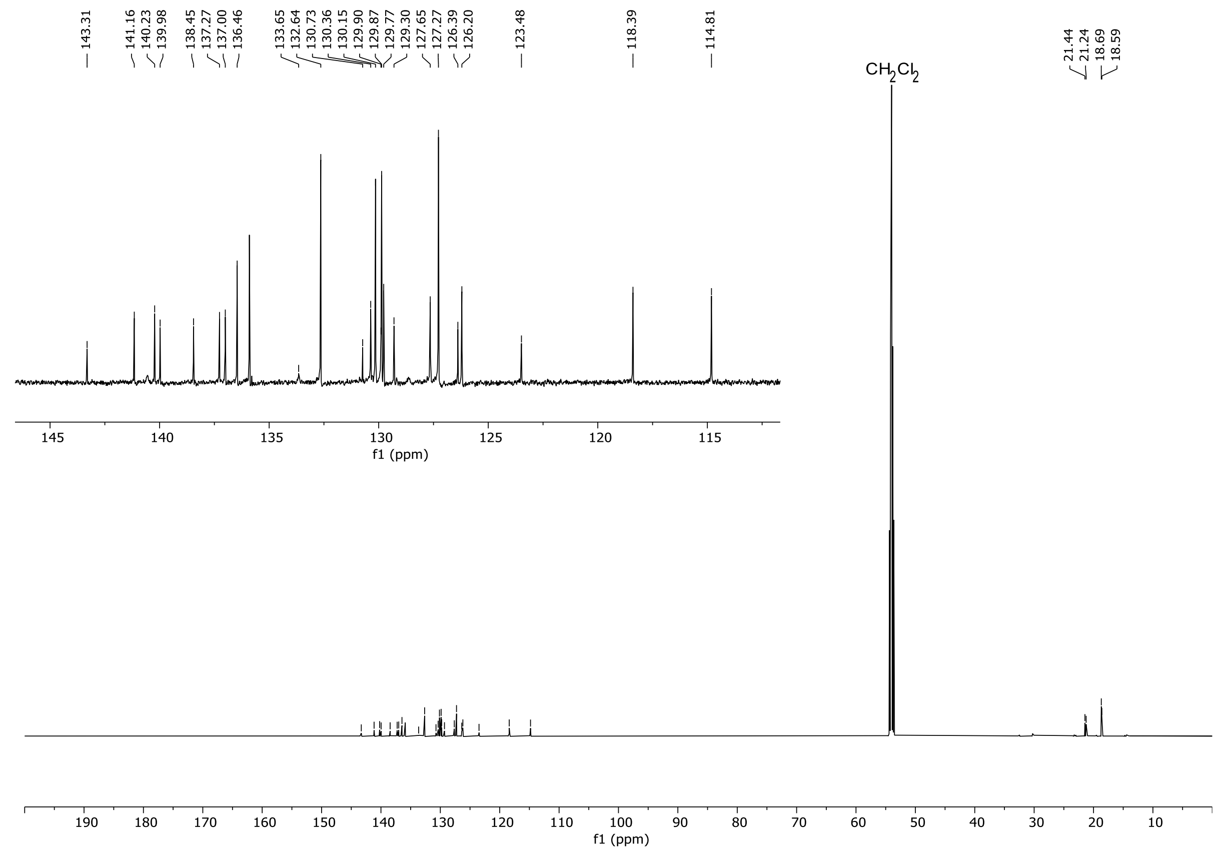

Figure S8. ^{13}C NMR (151 MHz, CD_2Cl_2) of molecule $\mathbf{1}_{\mathbf{NBNBN}}$.

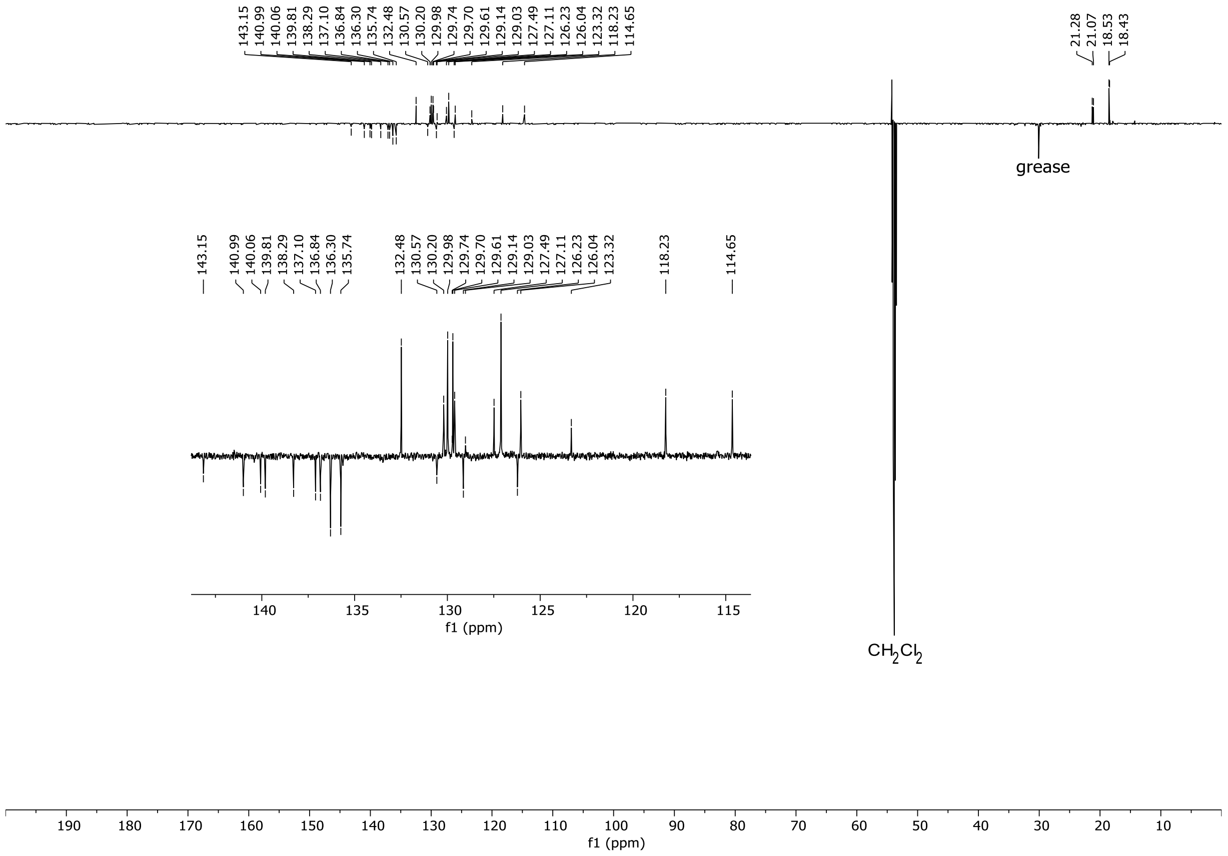

Figure S9. ^{13}C NMR (151 MHz, CD_2Cl_2) DEPT-135 of molecule $\mathbf{1}_{\mathbf{NBNBN}}$.

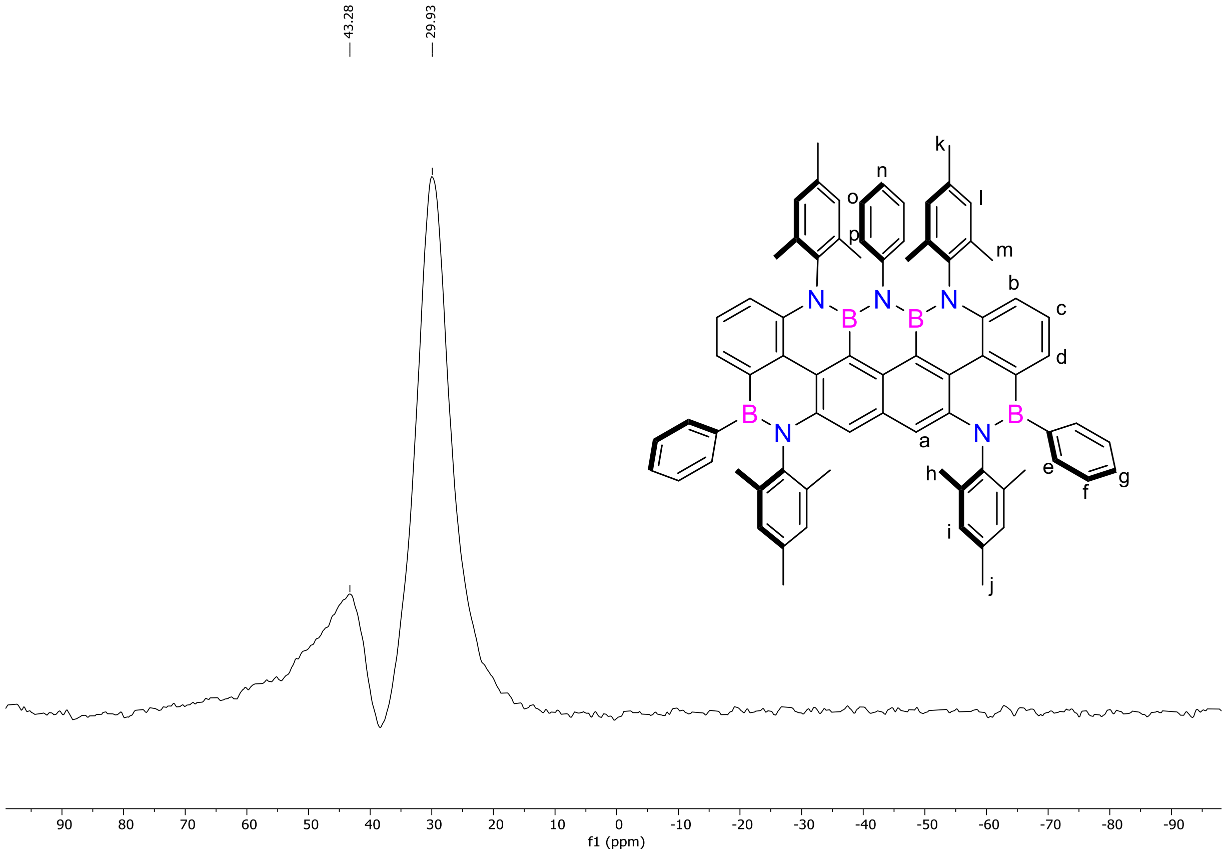

Figure S10. ^{11}B NMR (193 MHz, CD_2Cl_2) of molecule **1_{NBNBN}**.

Molecule 2_{NBNBN}

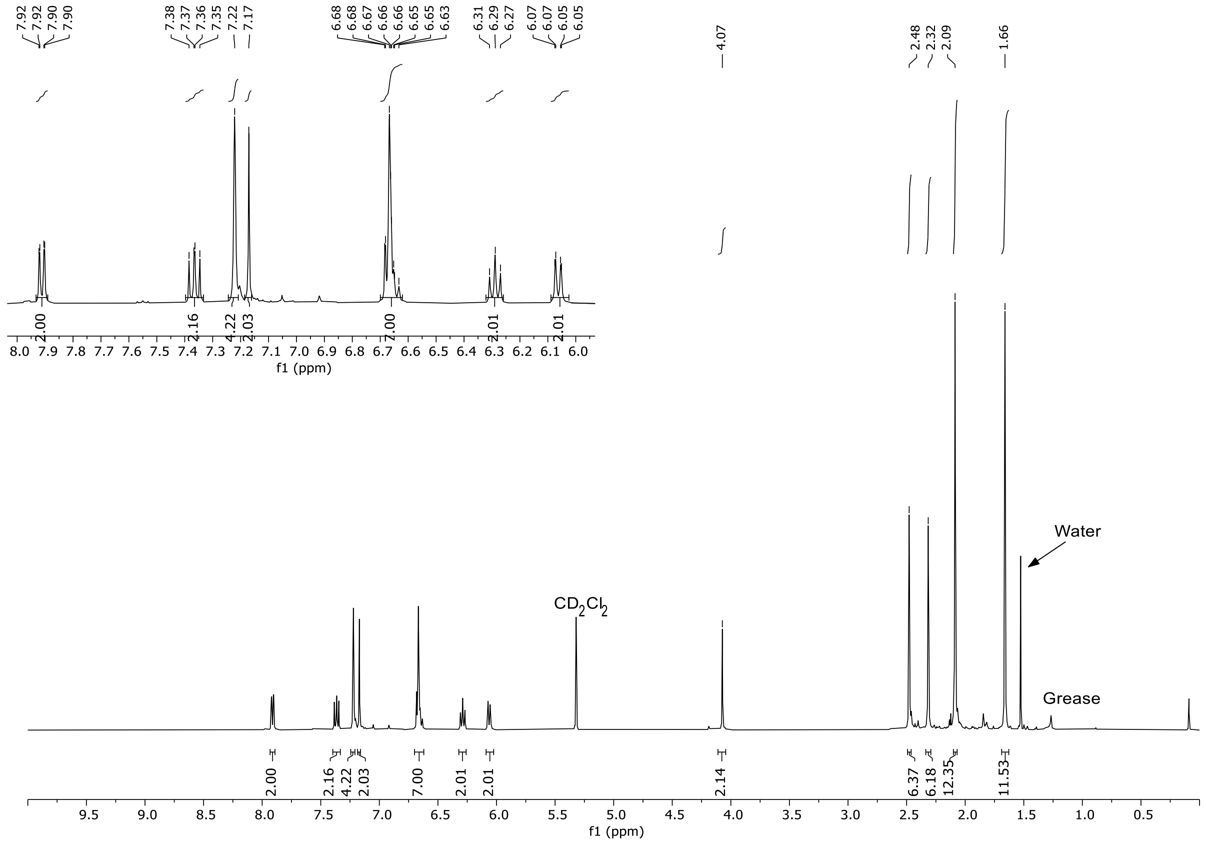

Figure S11. ^{1}H NMR (400 MHz, CD_2Cl_2) of molecule **2_{NBNBN}**.

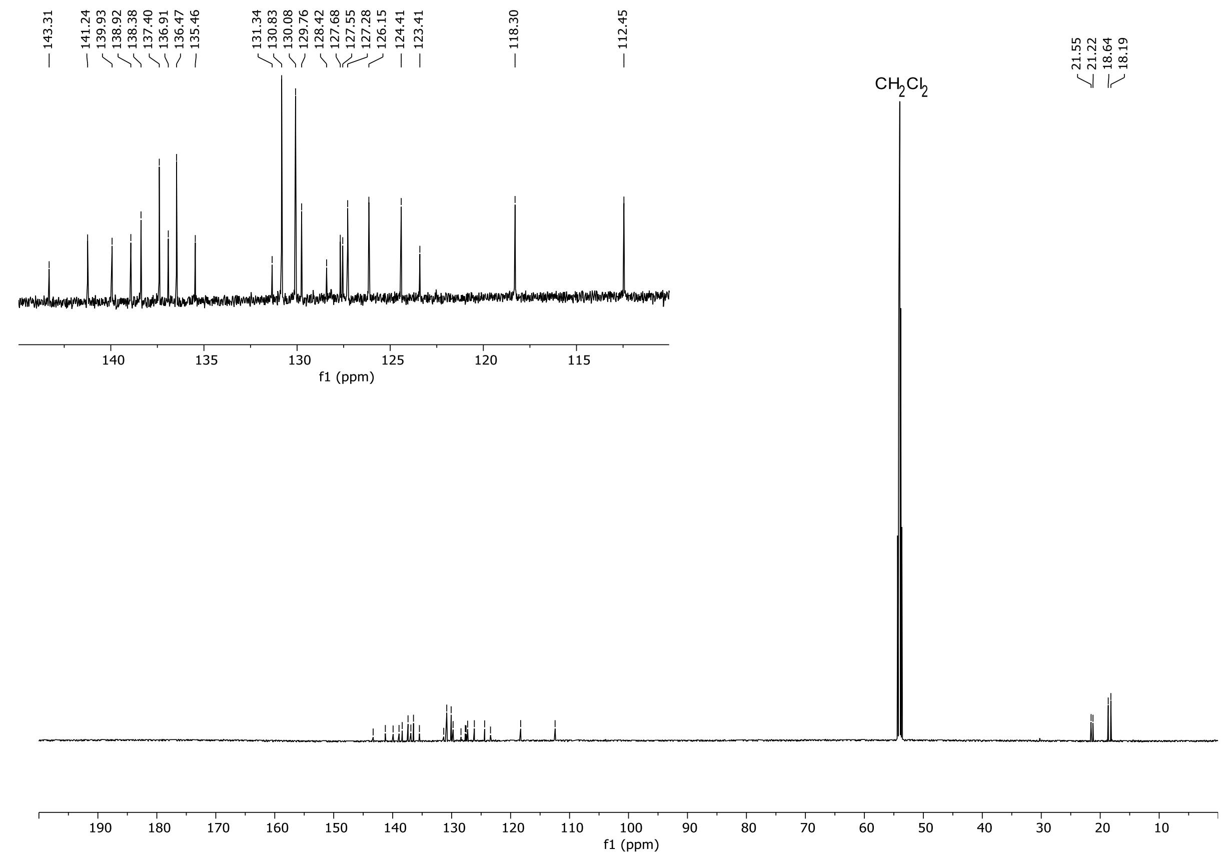

Figure S12. ^{13}C NMR (151 MHz, CD_2Cl_2) of molecule $\mathbf{2}_{\mathbf{NBNBN}}$.

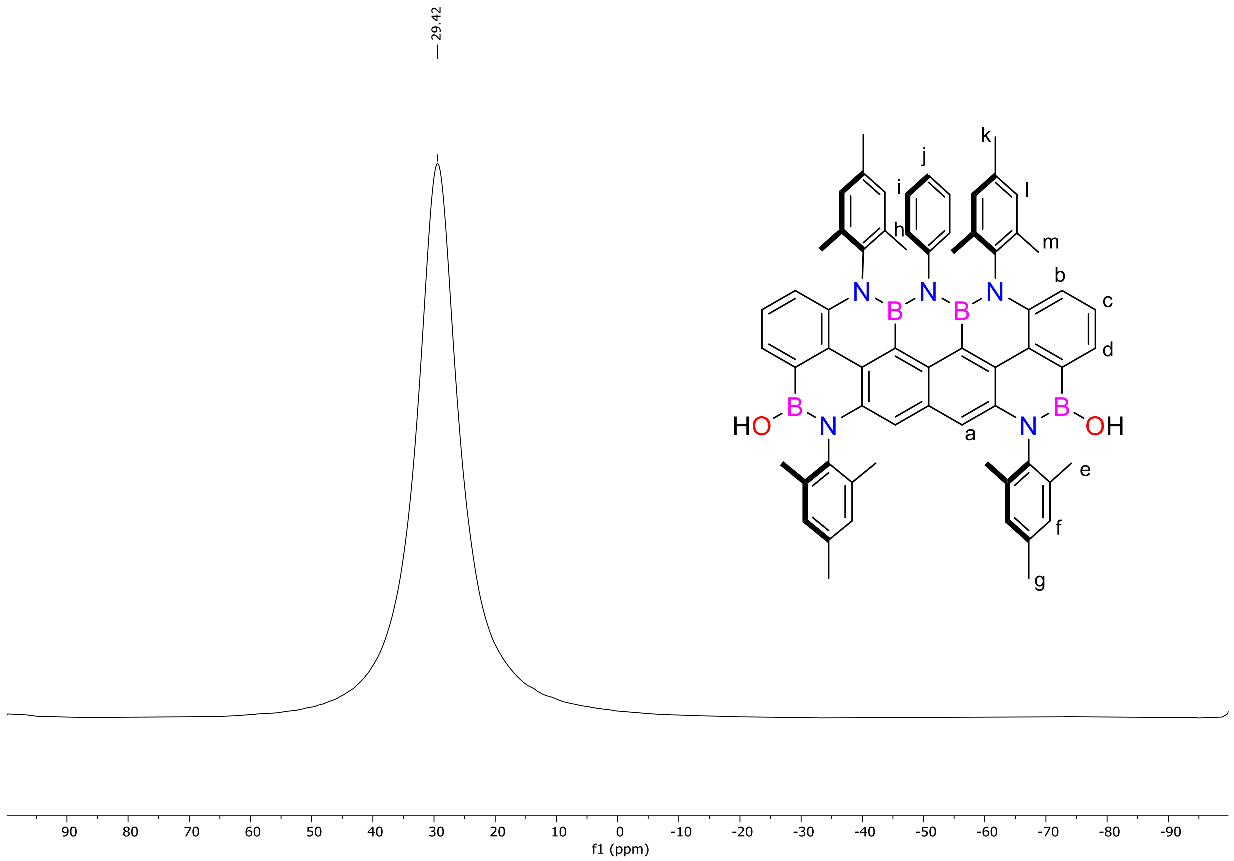

Figure S13. ^{11}B NMR (193 MHz, CD_2Cl_2) of molecule $\mathbf{2}_{\mathbf{NBNBN}}$.

Molecule $\mathbf{1_{NBOBN}}$

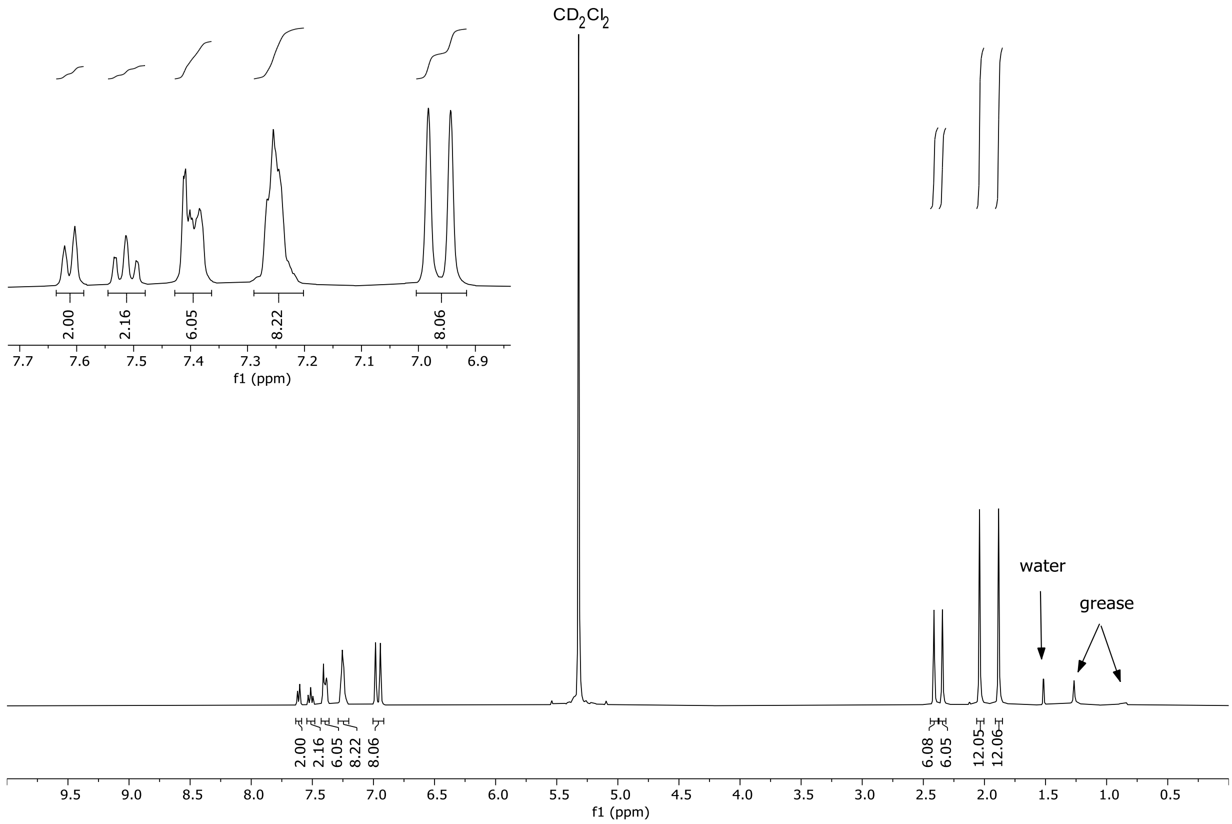

Figure S14. ^{1}H NMR (700 MHz, CD_2Cl_2) of molecule $\mathbf{1_{NBOBN}}$.

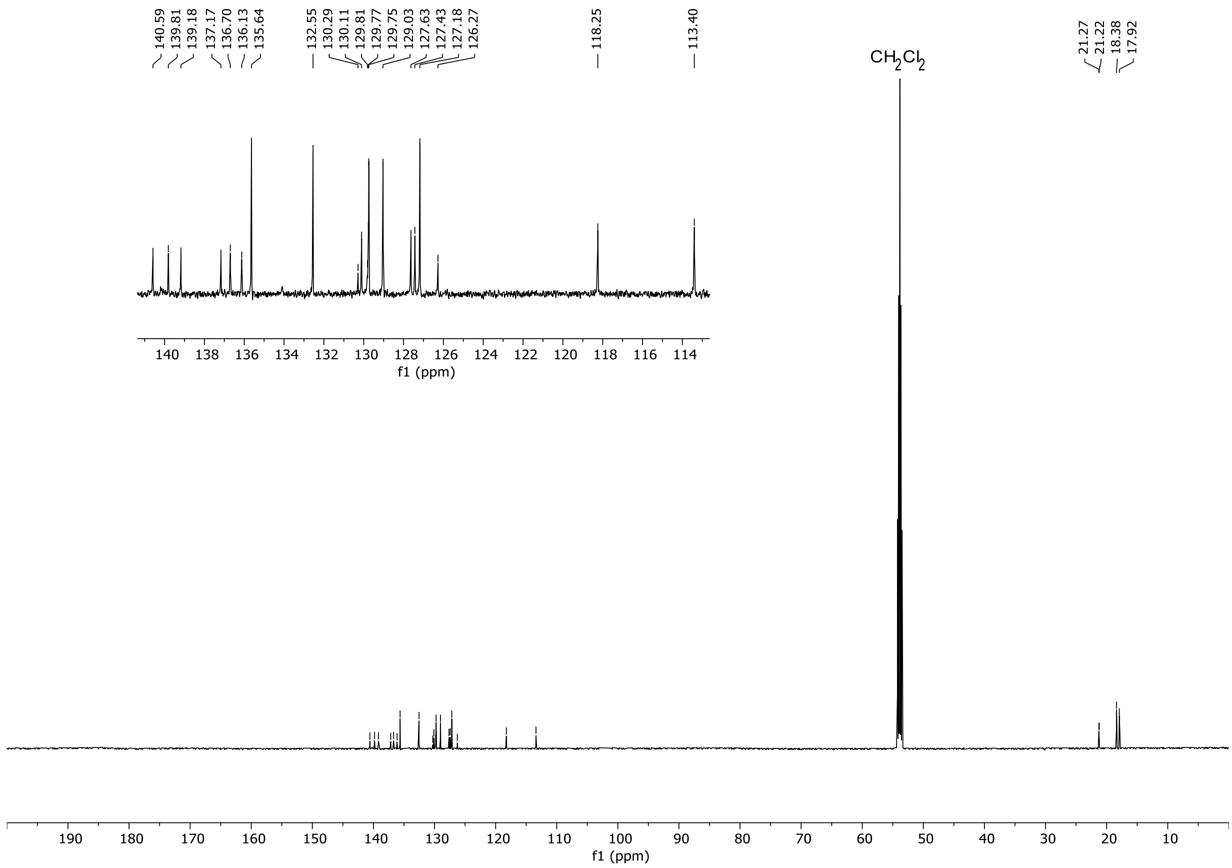

Figure S15. ^{13}C NMR (176 MHz, CD_2Cl_2) of molecule $\mathbf{1_{NBOBN}}$.

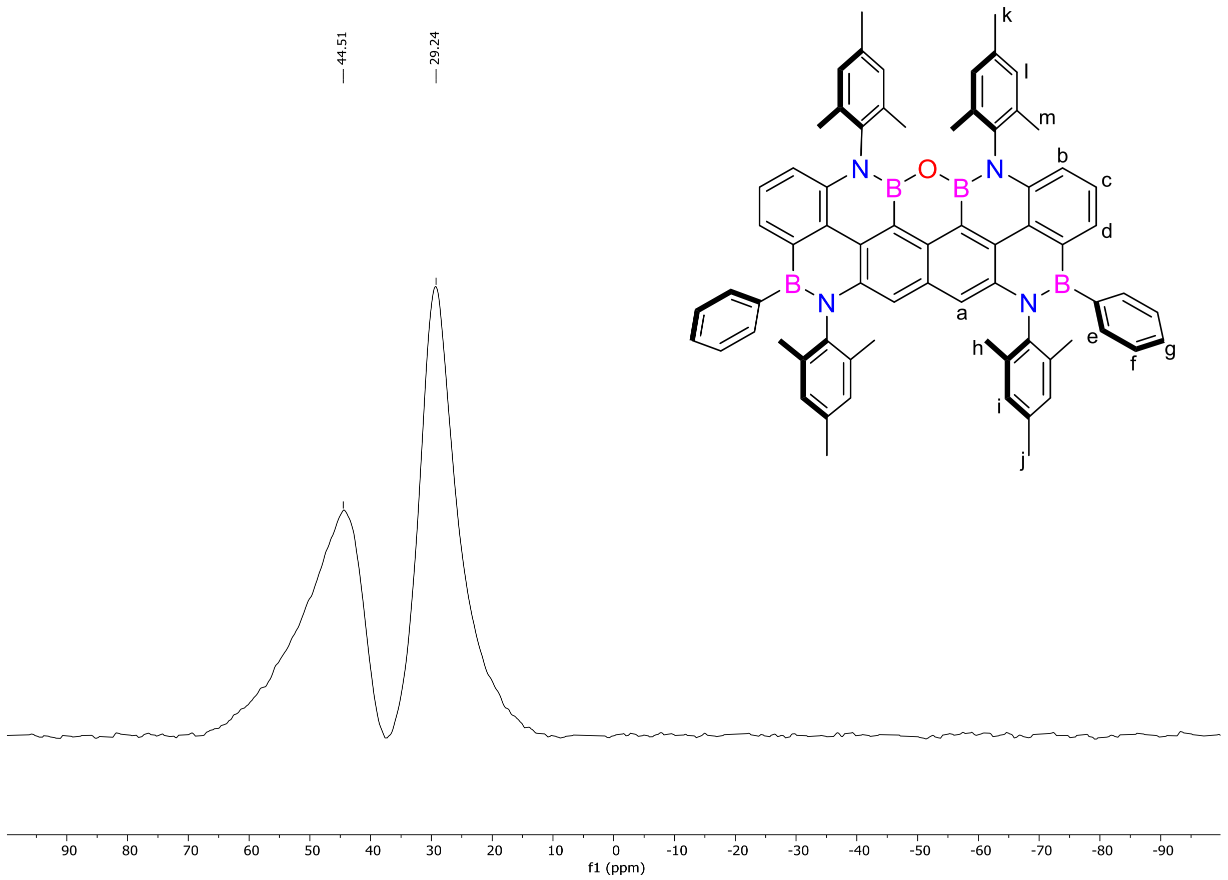

Figure S16. ^{11}B NMR (193 MHz, CD_2Cl_2) of molecule $\mathbf{1_{NBOBN}}$.

Molecule 2_{NBOBN}

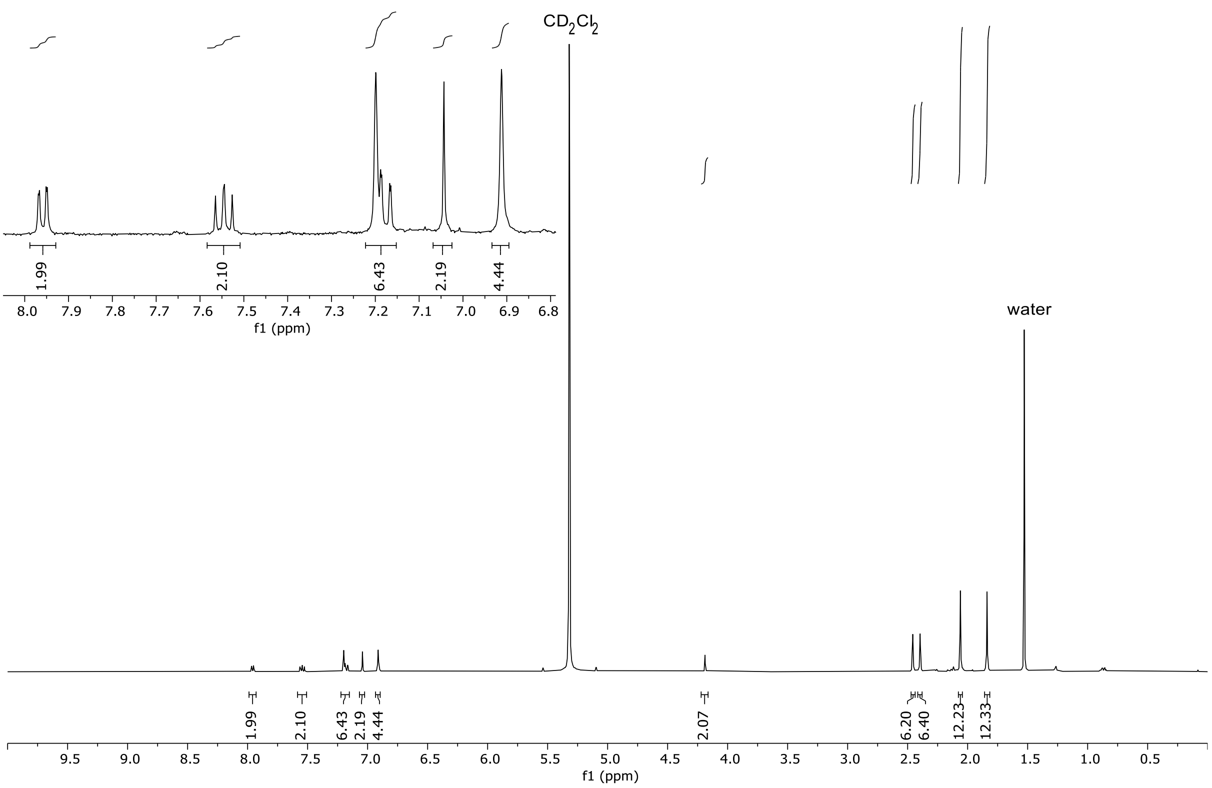

Figure S17. ^{1}H NMR (400 MHz, CD_2Cl_2) of molecule $\mathbf{2_{NBOBN}}$.

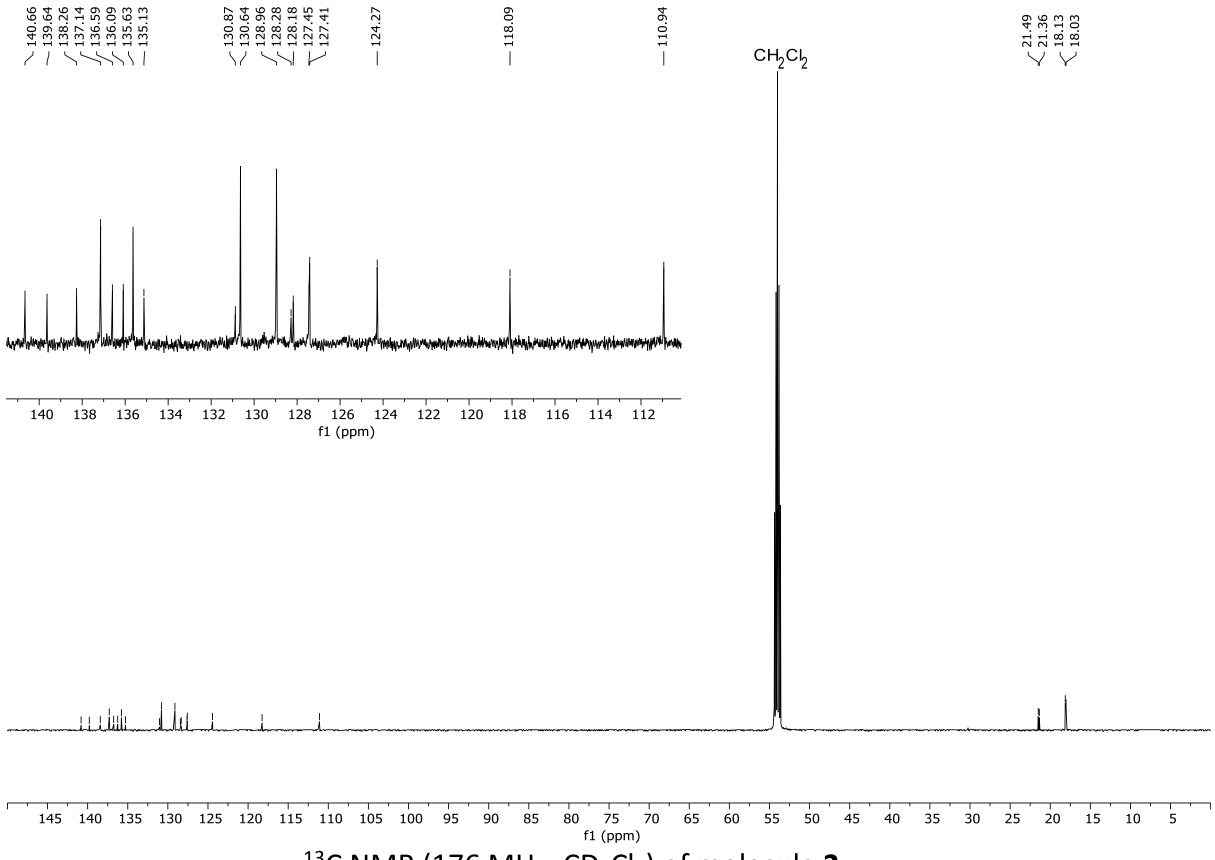

^{13}C NMR (176 MHz, CD_2Cl_2) of molecule $\mathbf{2_{NBOBN}}$.

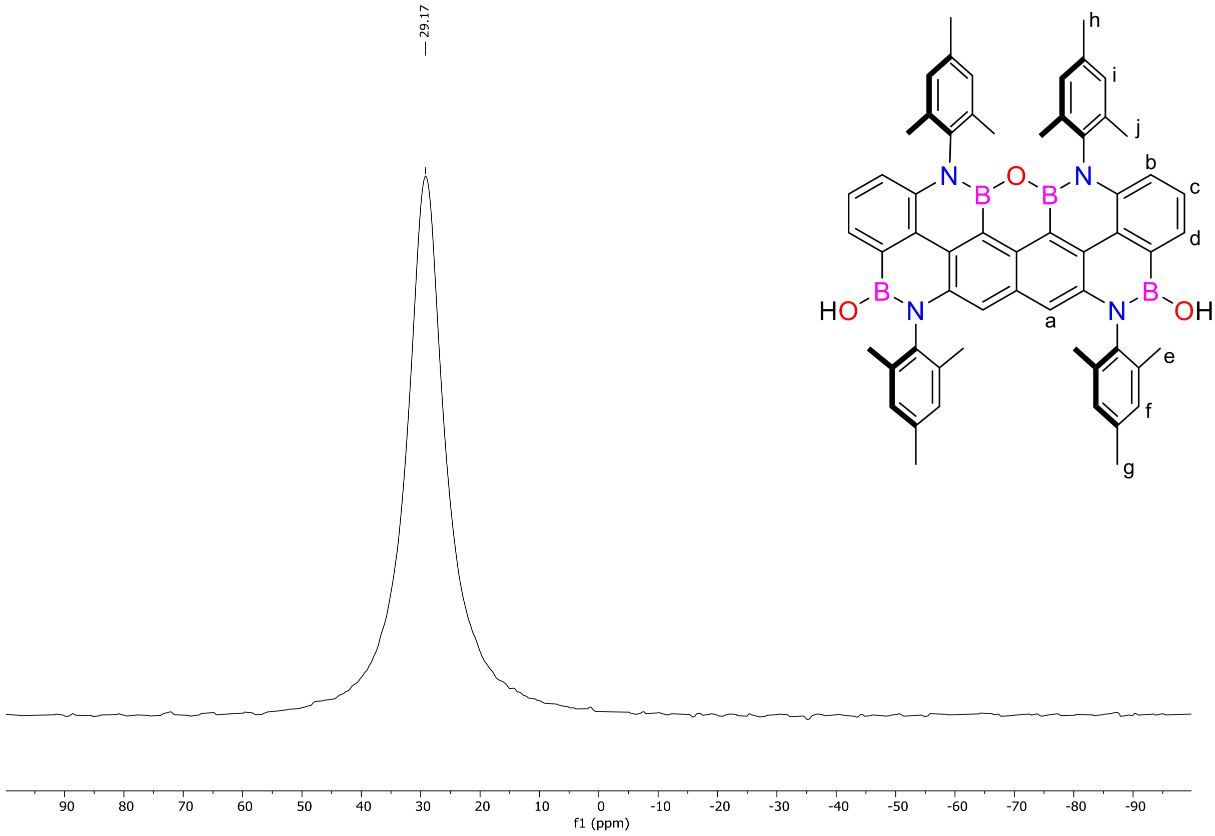

Figure S18. ^{11}B NMR (193 MHz, CD_2Cl_2) of molecule $\mathbf{2_{NBOBN}}$.

Molecule 4

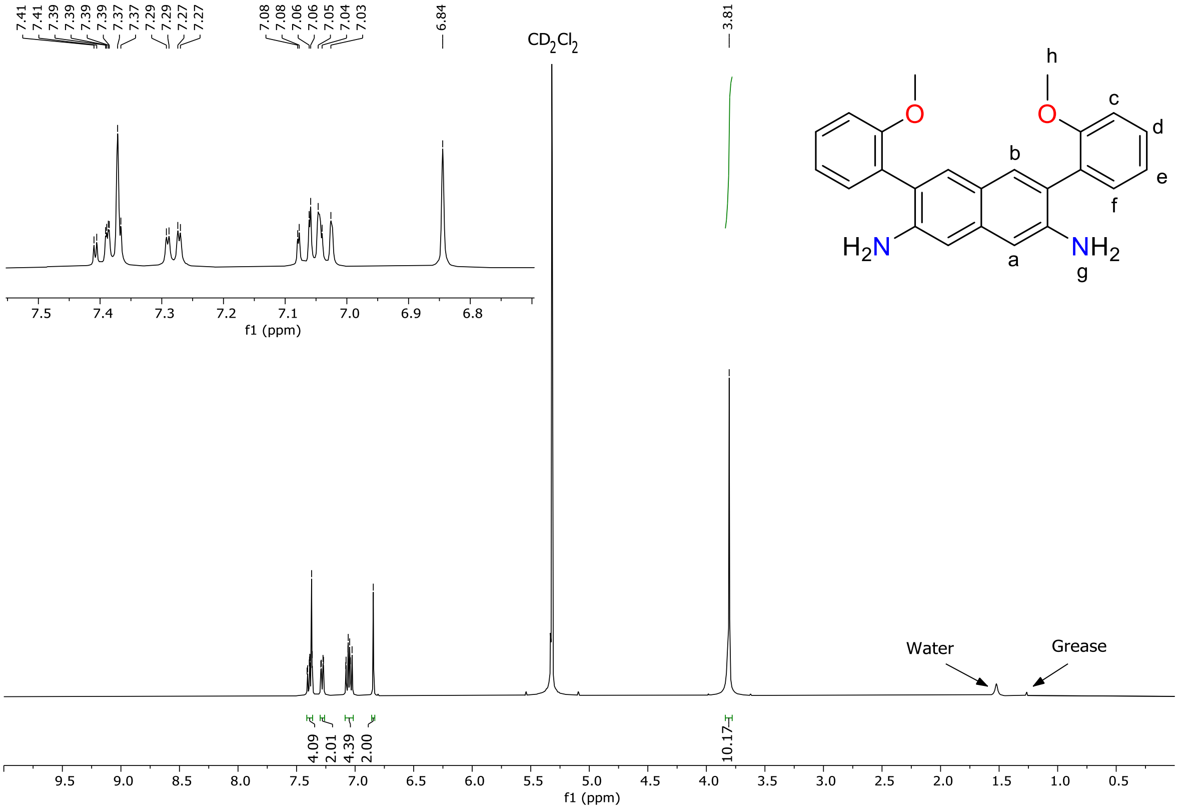

Figure S19. ^{1}H NMR (400 MHz, CD_2Cl_2) of molecule **4**.

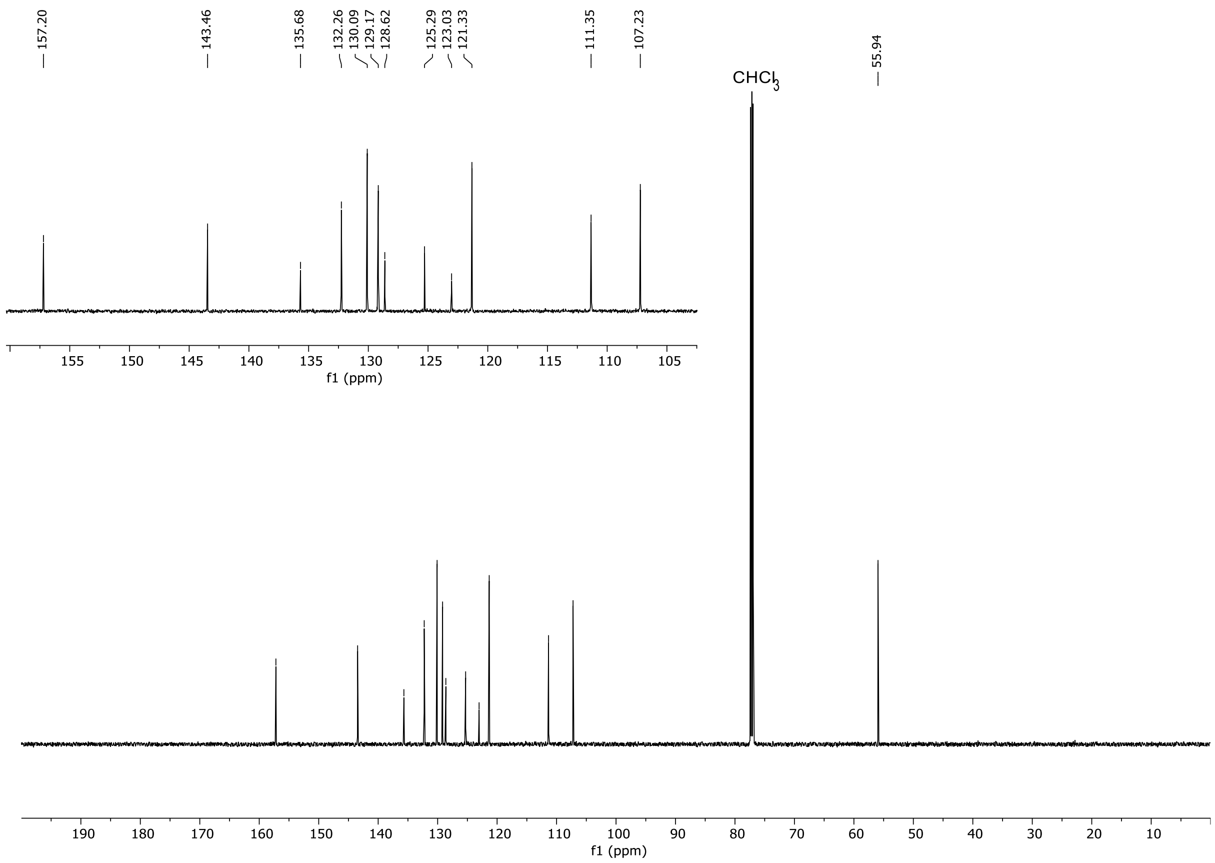

Figure S20. ^{13}C NMR (151 MHz, $CDCl_3$) of molecule **4**.

Molecule 5

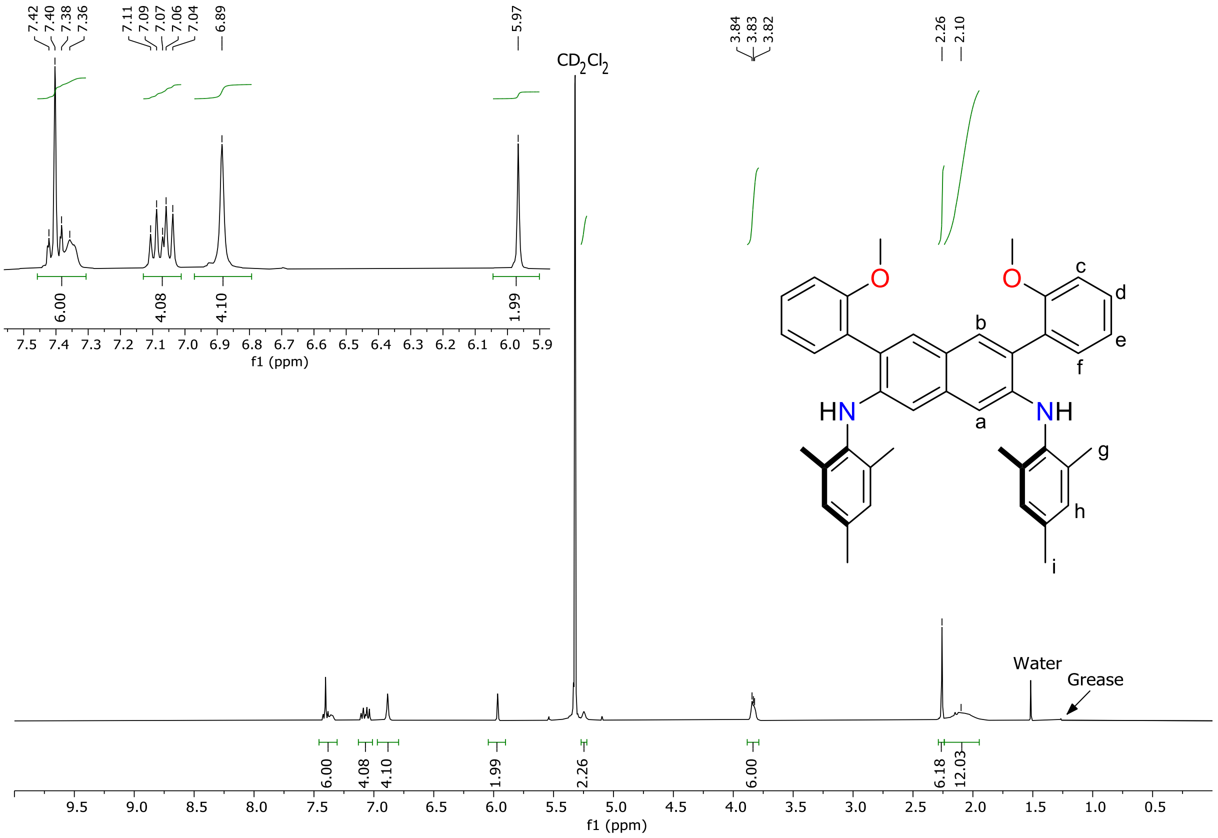

Figure S21. ^{1}H NMR (400 MHz, CD_2Cl_2) of molecule **5**.

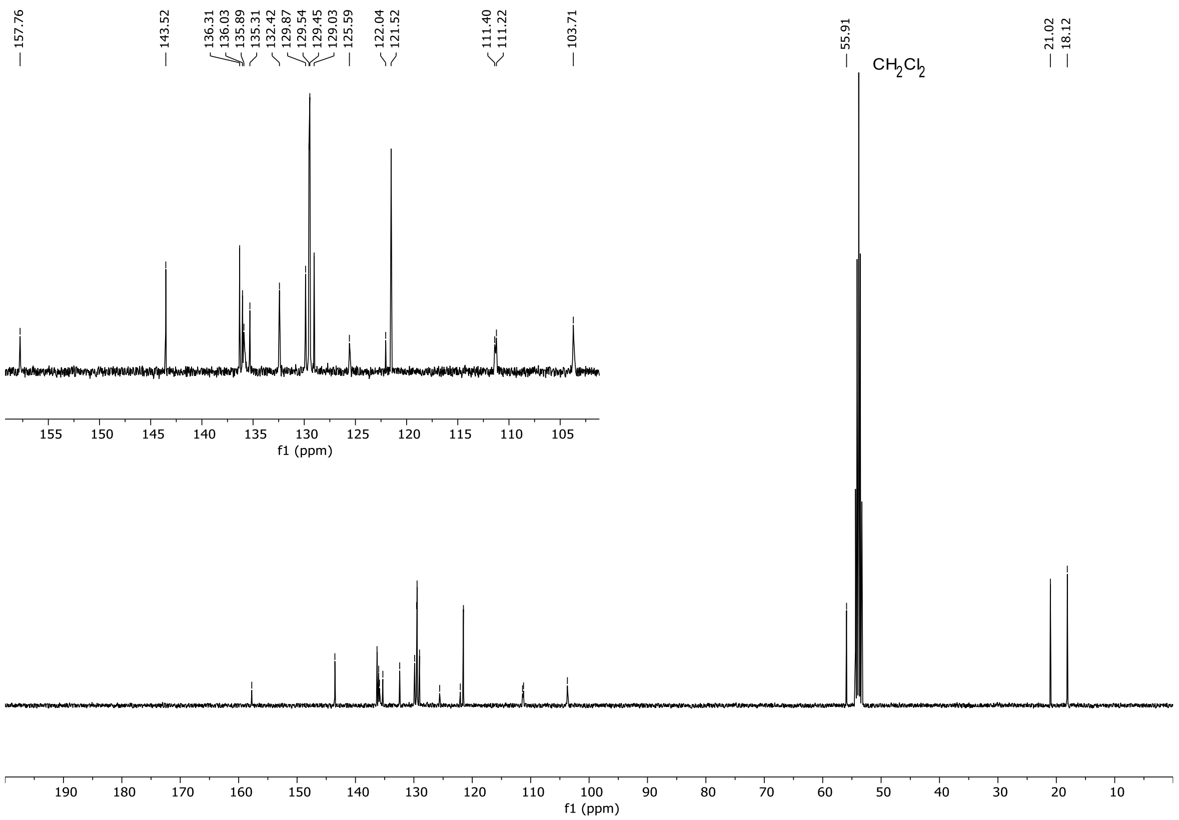

Figure S22. ^{13}C NMR (151 MHz, $CDCl_3$) of molecule **5**.

Molecule $\mathbf{1_{OBOBO}}$

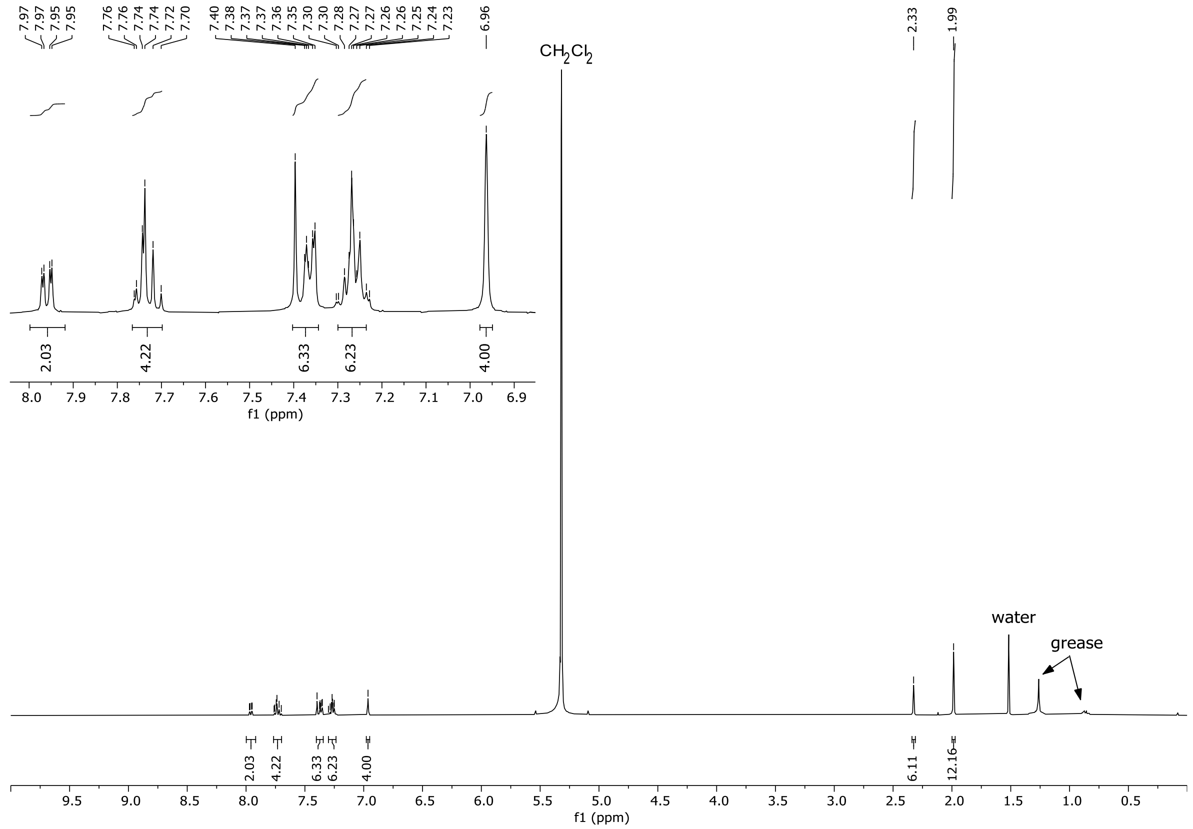

Figure S23. ^{1}H NMR (400 MHz, CD_2Cl_2) of molecule $\mathbf{1_{OBOBO}}$.

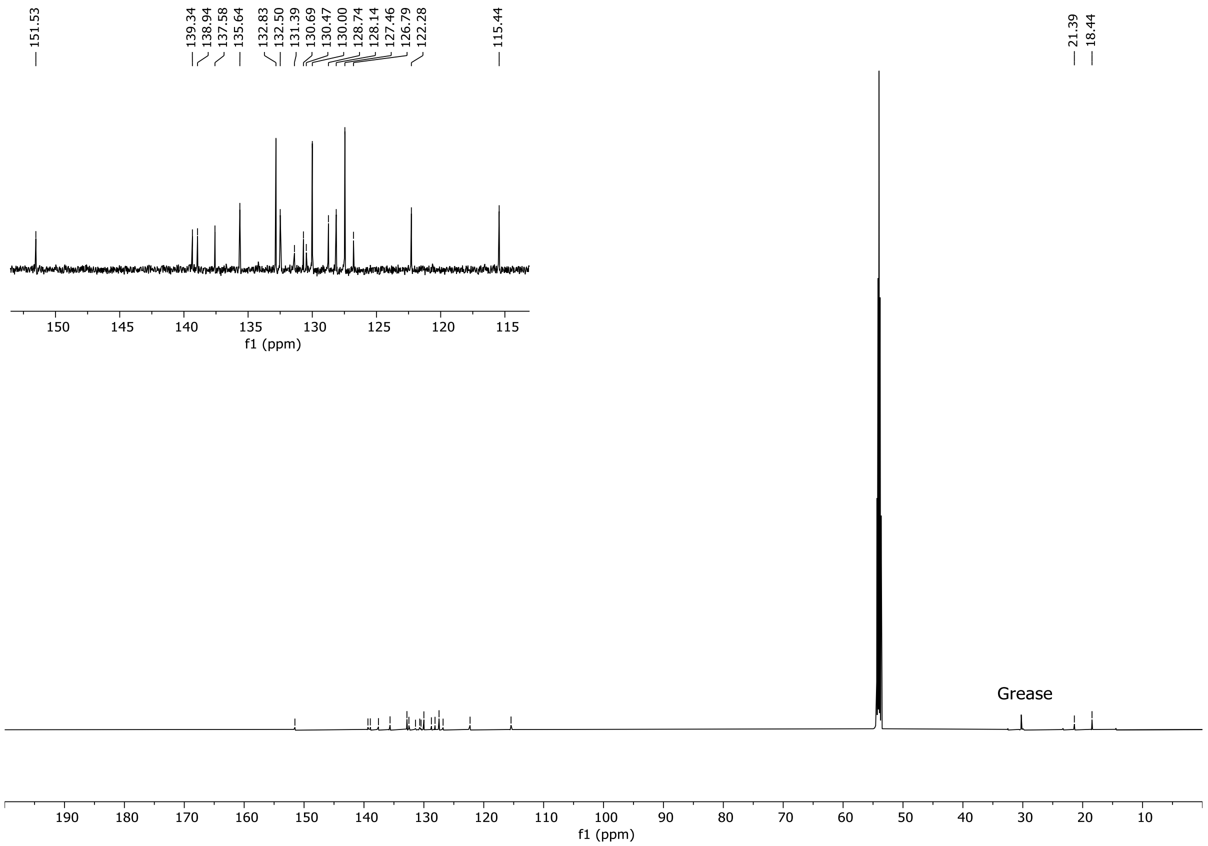

Figure S24. ^{13}C NMR (151 MHz, CD_2Cl_2) of molecule $\mathbf{1}_{\mathbf{OBOBO}}$.

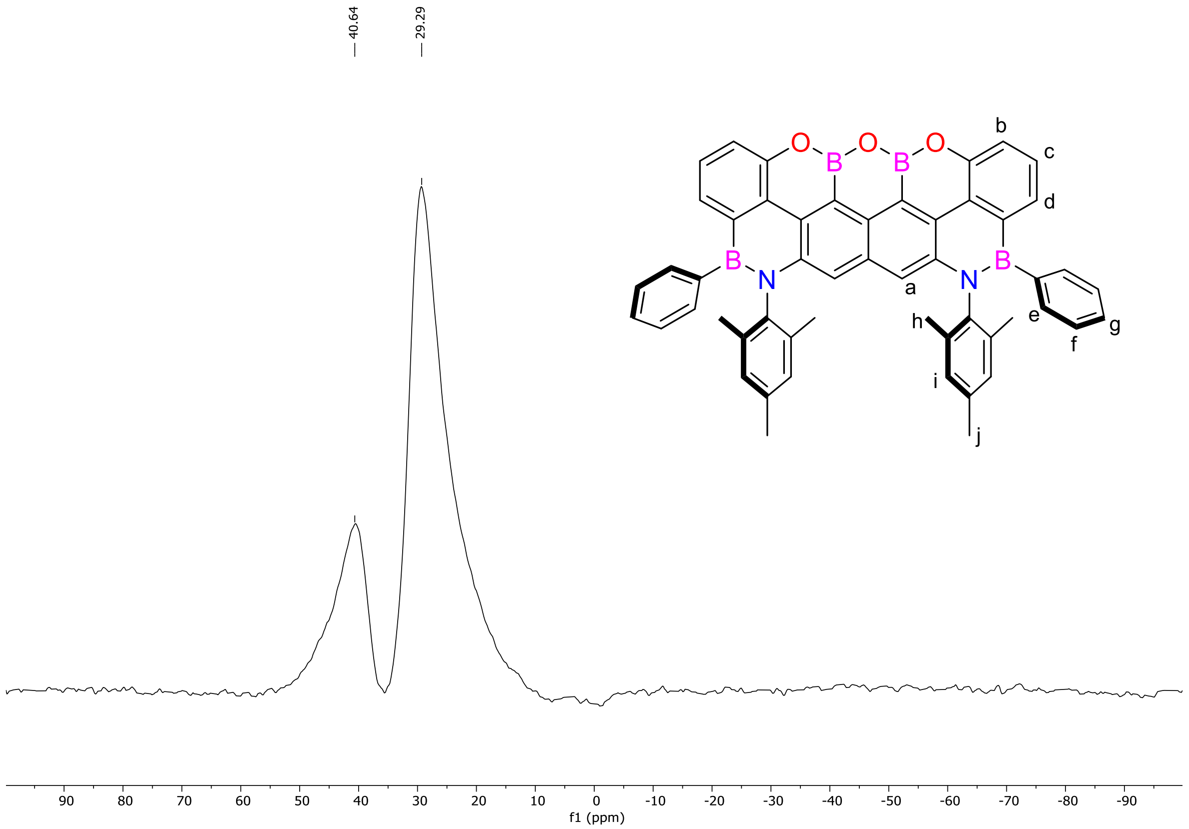

Figure S25. ^{11}B NMR (193 MHz, CD_2Cl_2) of molecule $\mathbf{1}_{\mathbf{OBOBO}}$.

Molecule 2_{OBOBO}

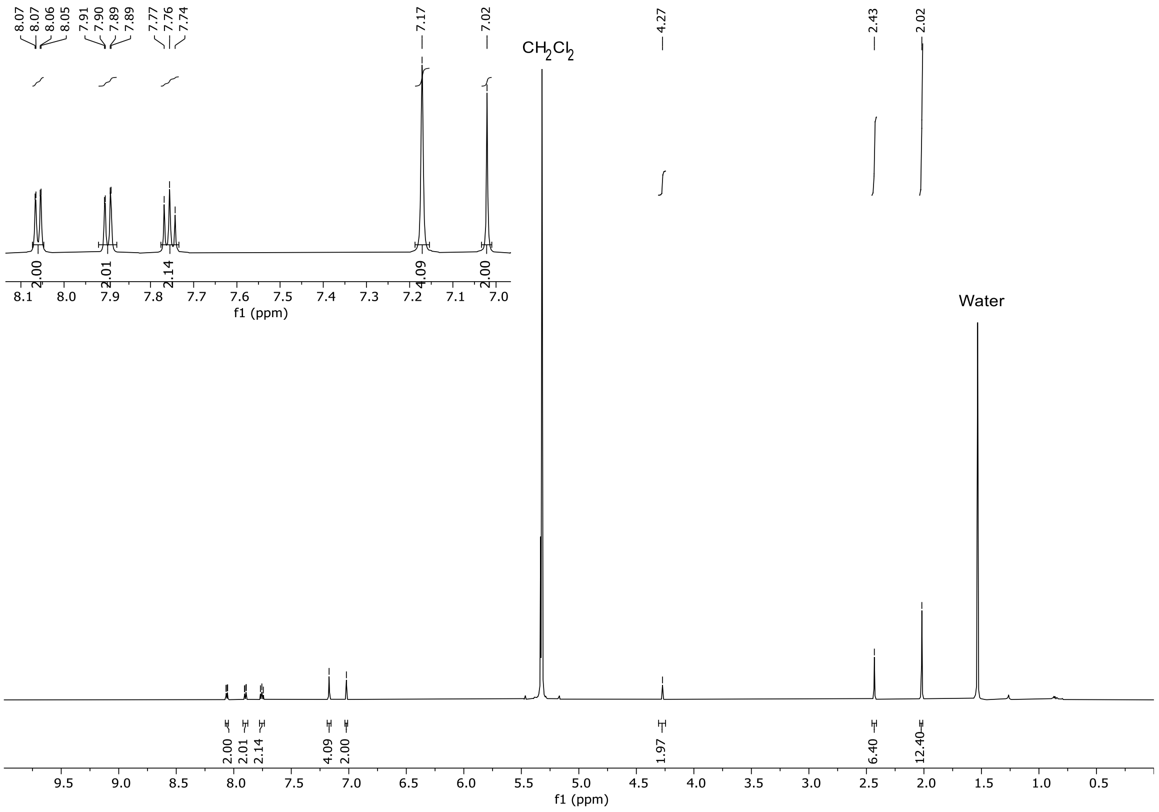

Figure S26. ^{1}H NMR (600 MHz, CD_2Cl_2) of molecule **2_{OBOBO}**.

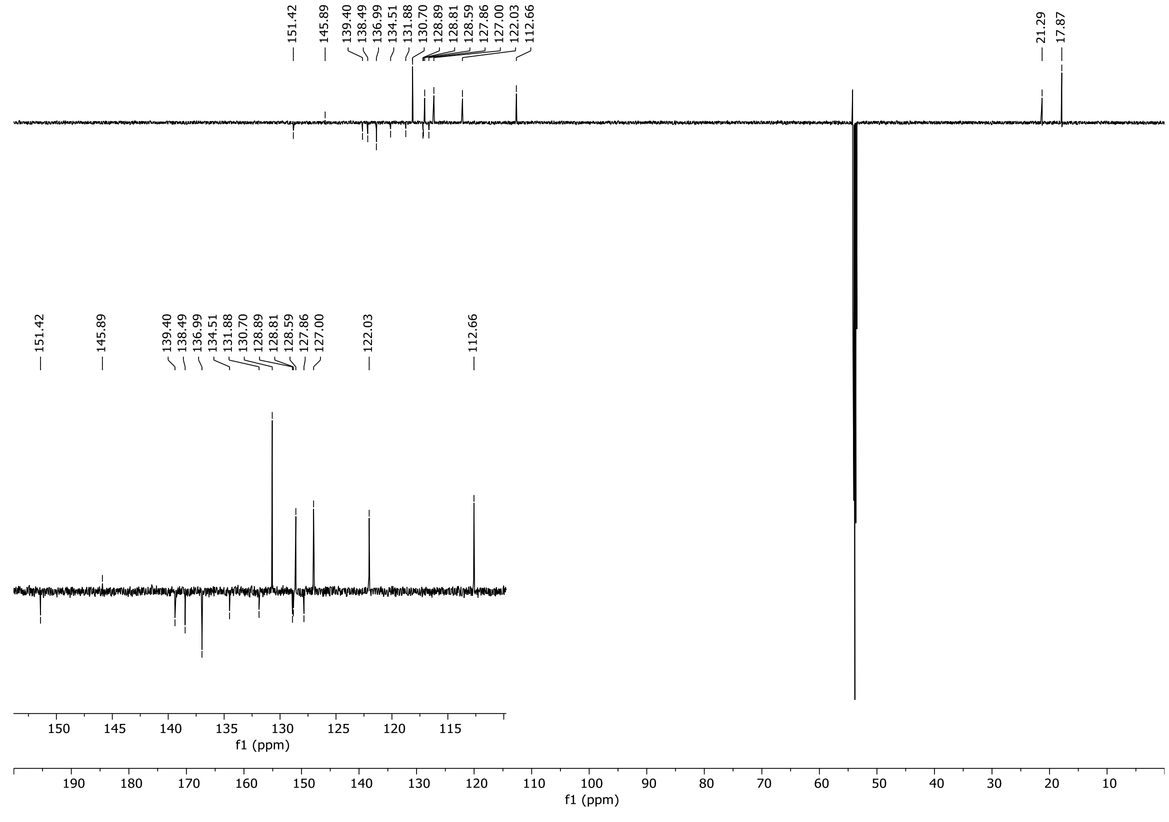

Figure S27. ^{13}C NMR (151 MHz, CD_2Cl_2) DEPT-135 of molecule **2_{OBOBO}**.

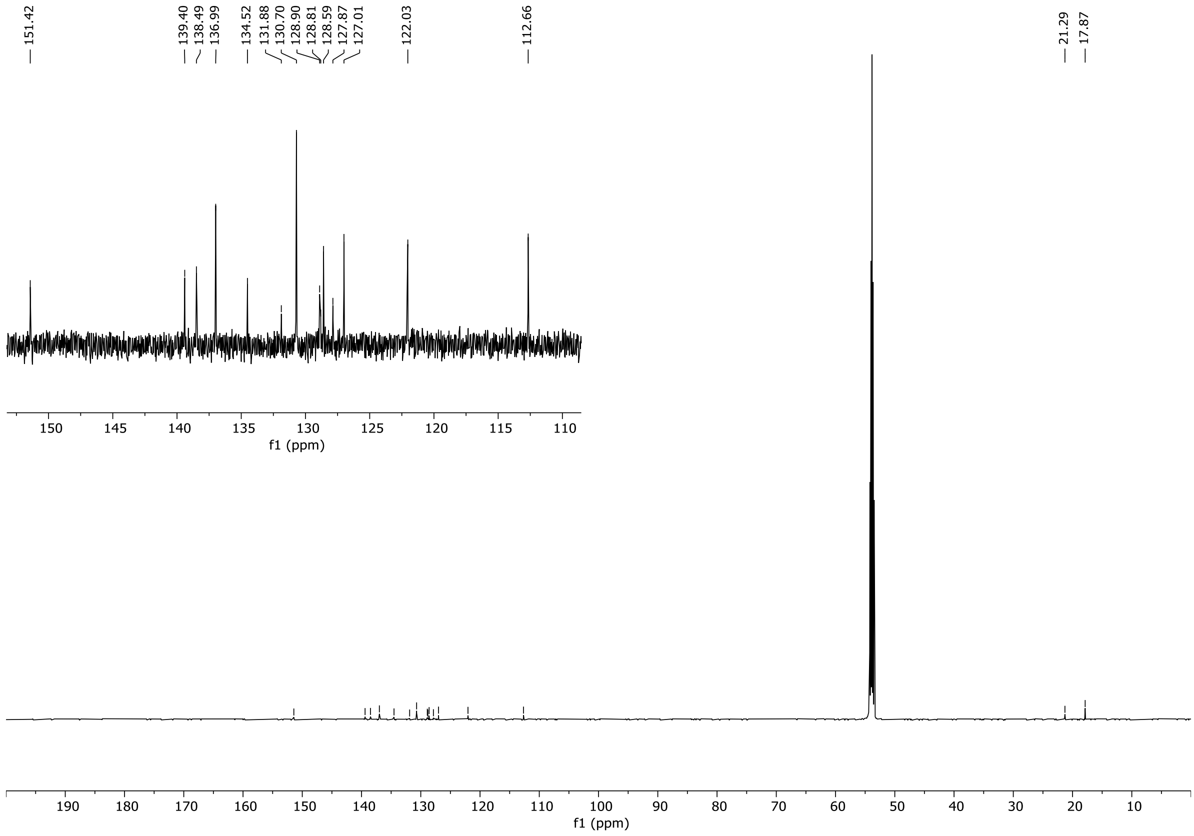

Figure S28. ^{13}C NMR (151 MHz, CD_2Cl_2) of molecule $\mathbf{2_{OBOBO}}$.

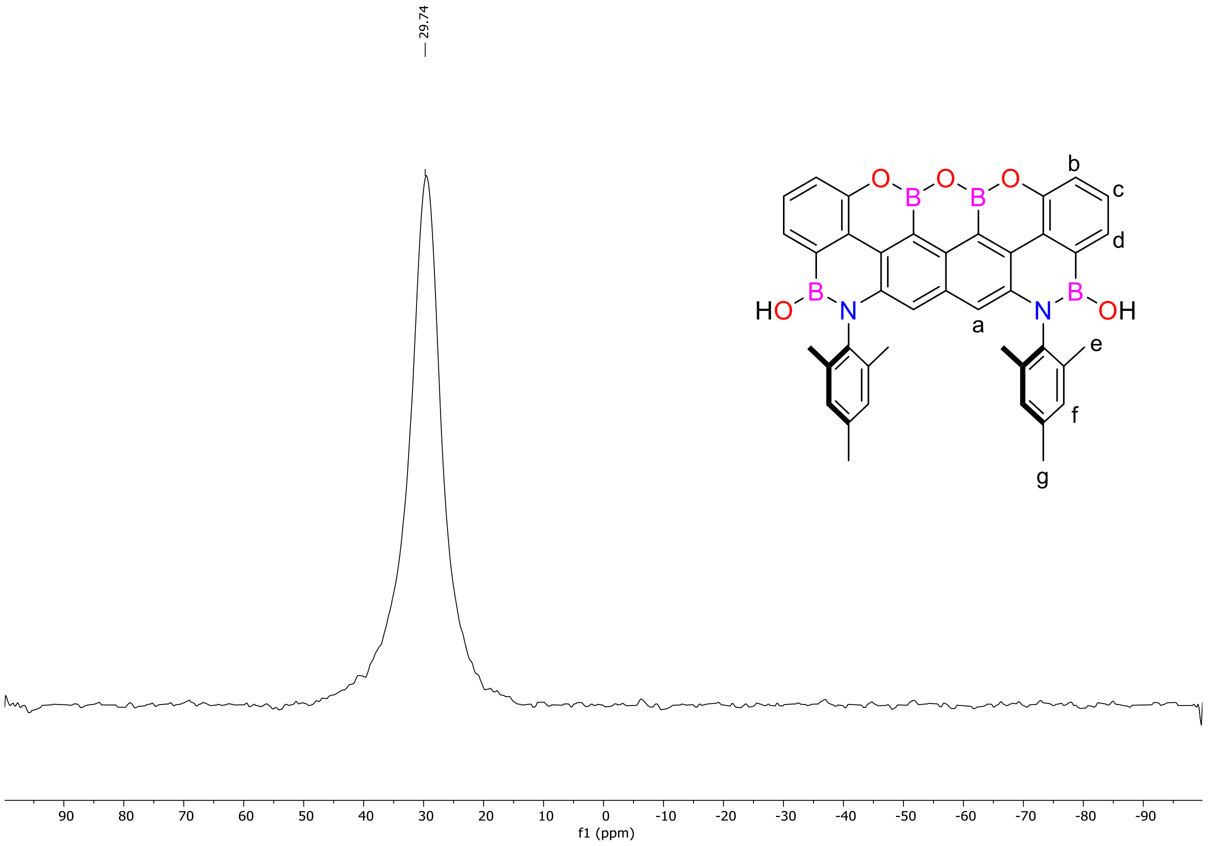

Figure S29. ^{11}B NMR (193 MHz, CD_2Cl_2) of molecule $\mathbf{2_{OBOBO}}$.

Molecule 2_{OBNBO}

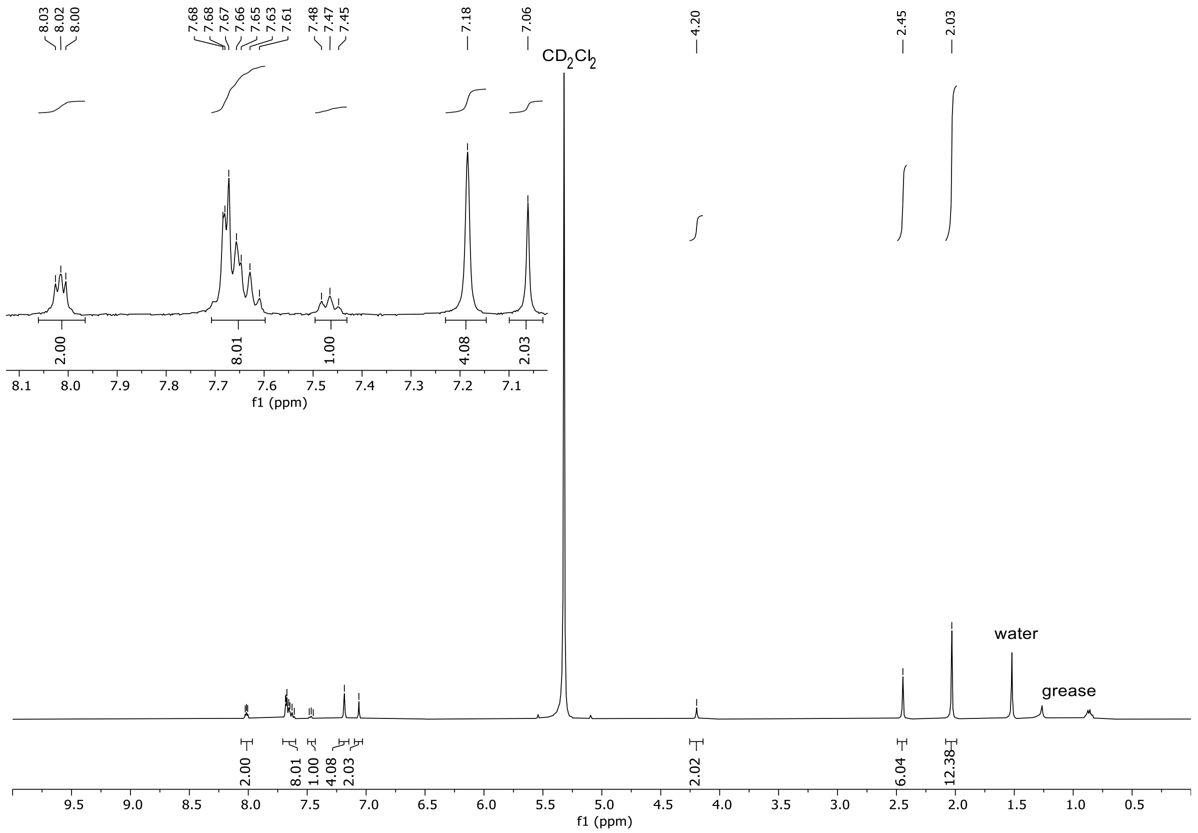

Figure S30. ^{1}H NMR (400 MHz, CD_2Cl_2) of molecule $\mathbf{2_{OBNBO}}$.

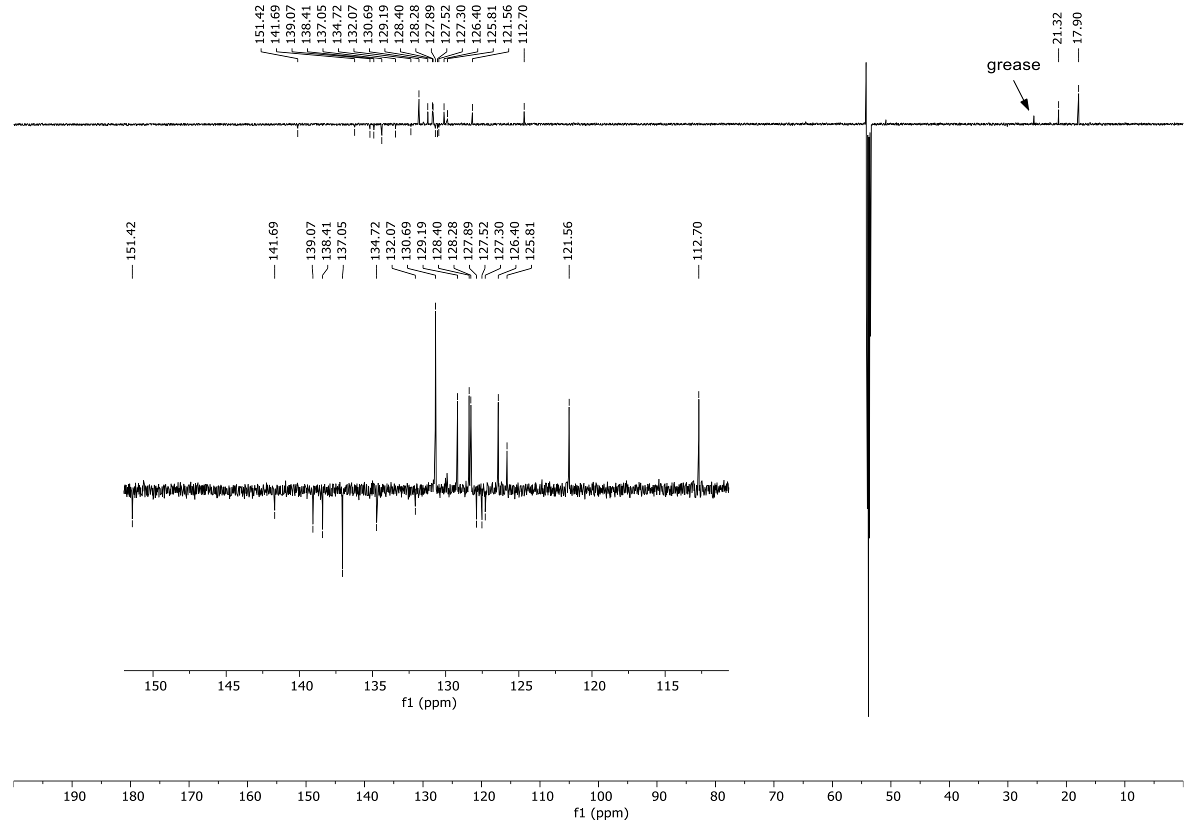

Figure S31. ^{13}C NMR (151 MHz, $CDCl_3$) of molecule $\mathbf{2_{OBNBO}}$.

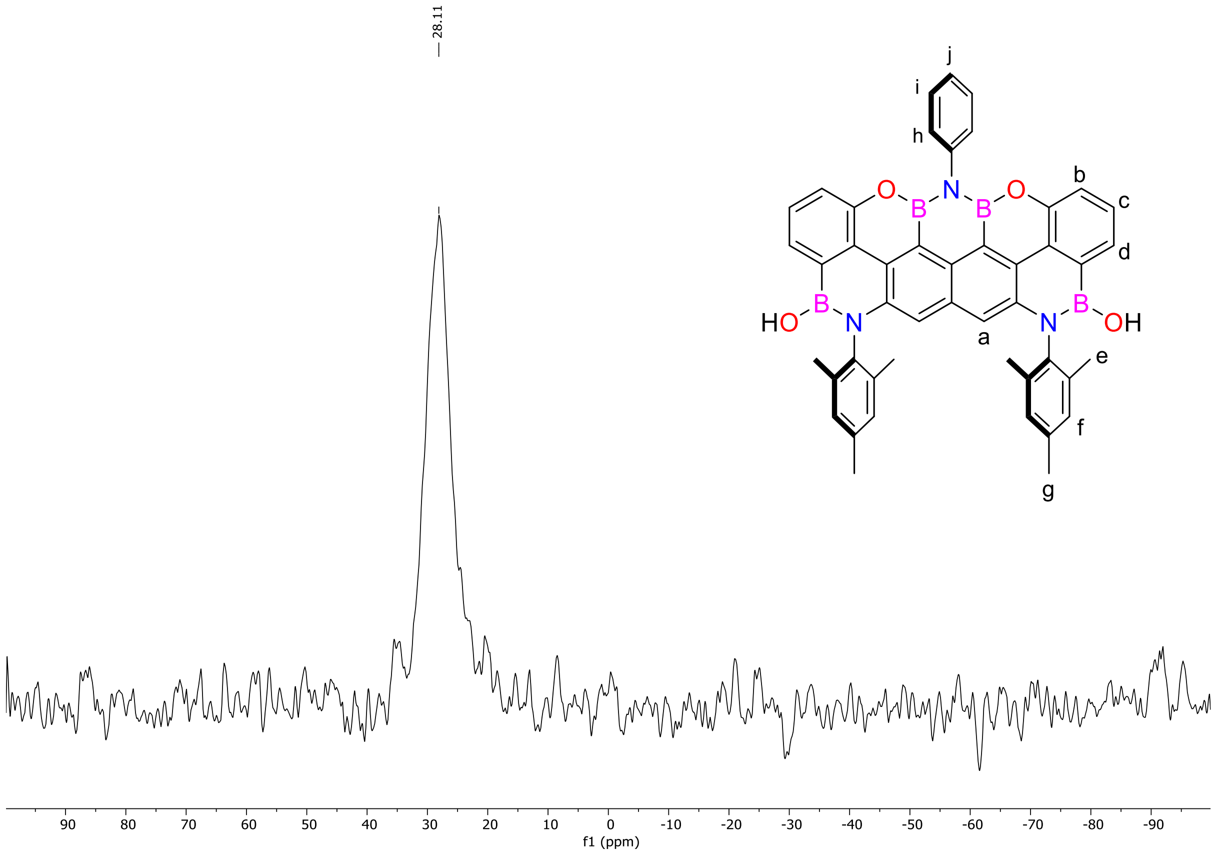

Figure S32. ^{11}B NMR (193 MHz, CD_2Cl_2) of molecule $\mathbf{2_{OBNBO}}$.

4. Mass spectra

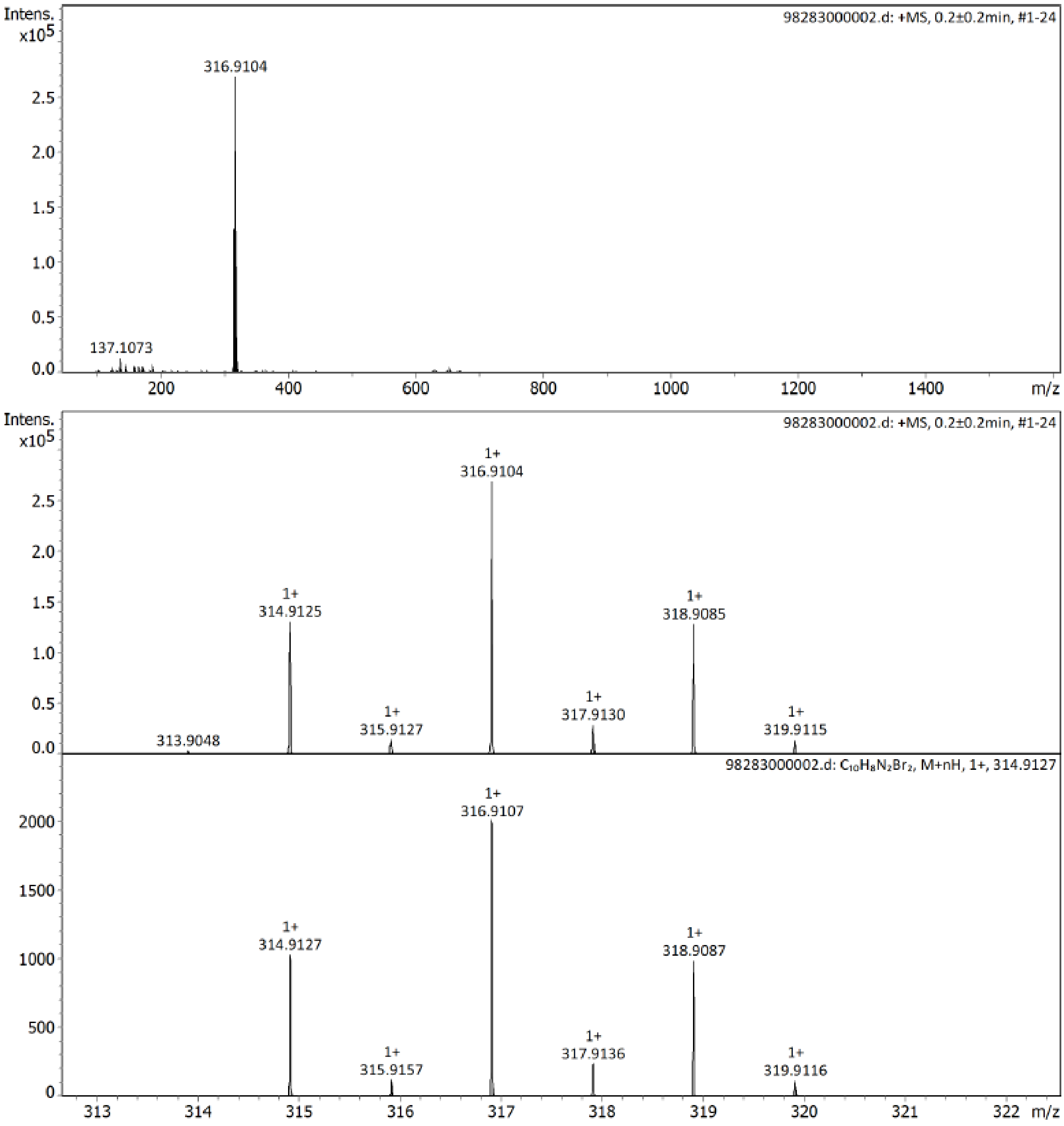

Figure S33. HRMS spectrum (ESI) of molecule **1**.

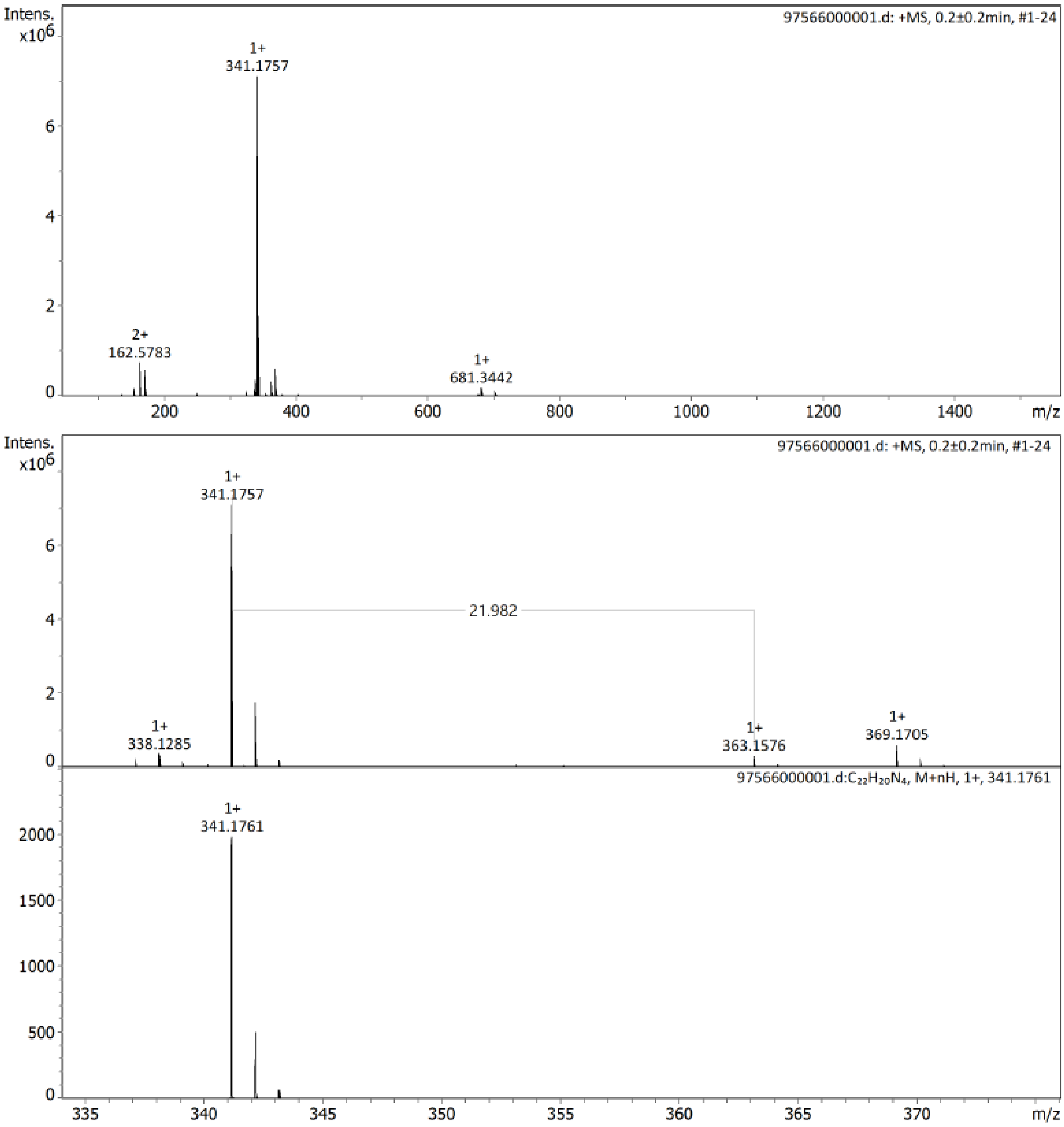

Figure S34. HRMS spectrum (ESI) of molecule **2**.

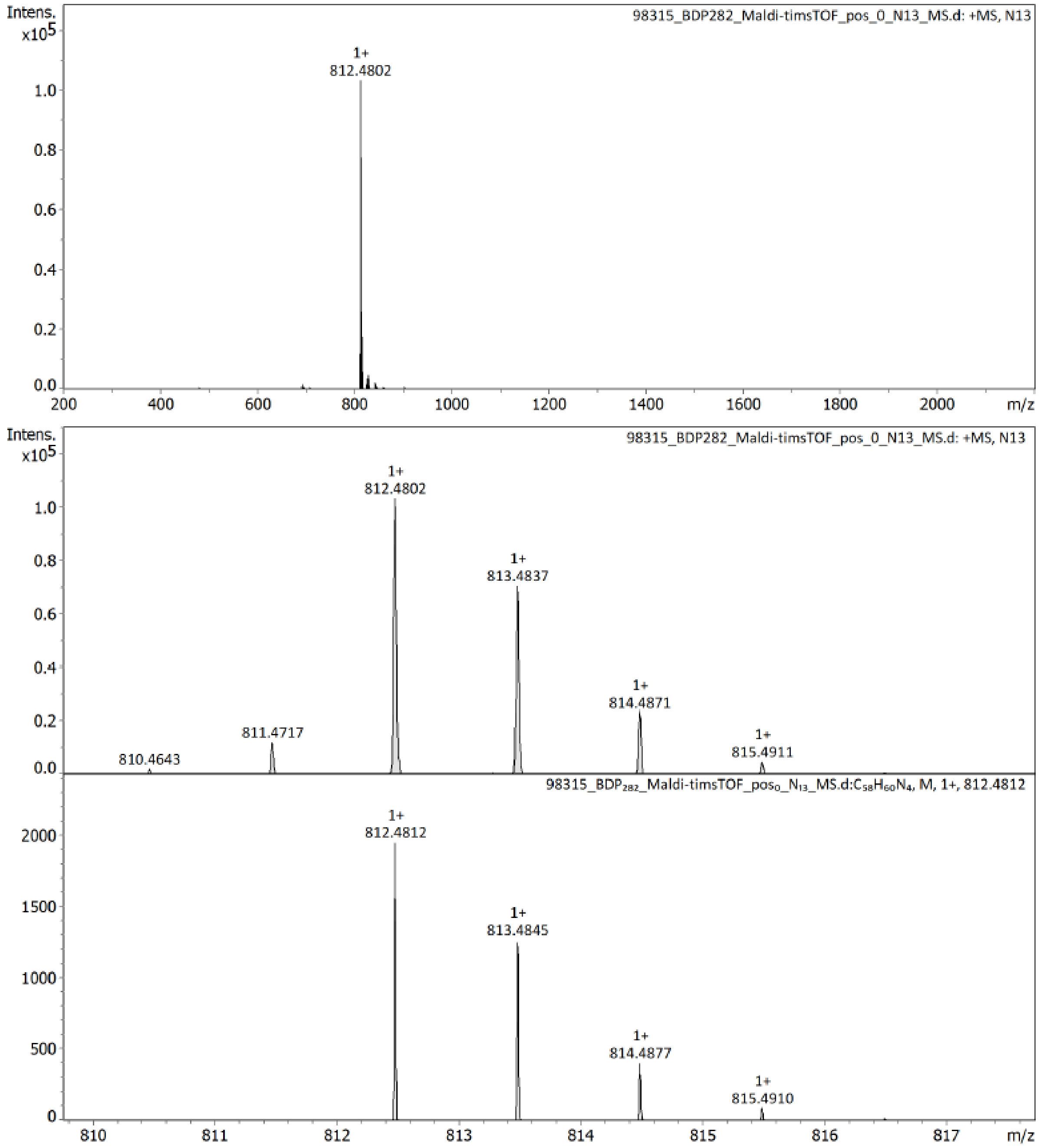

Figure S35. HRMS spectrum (MALDI) of molecule **3**.

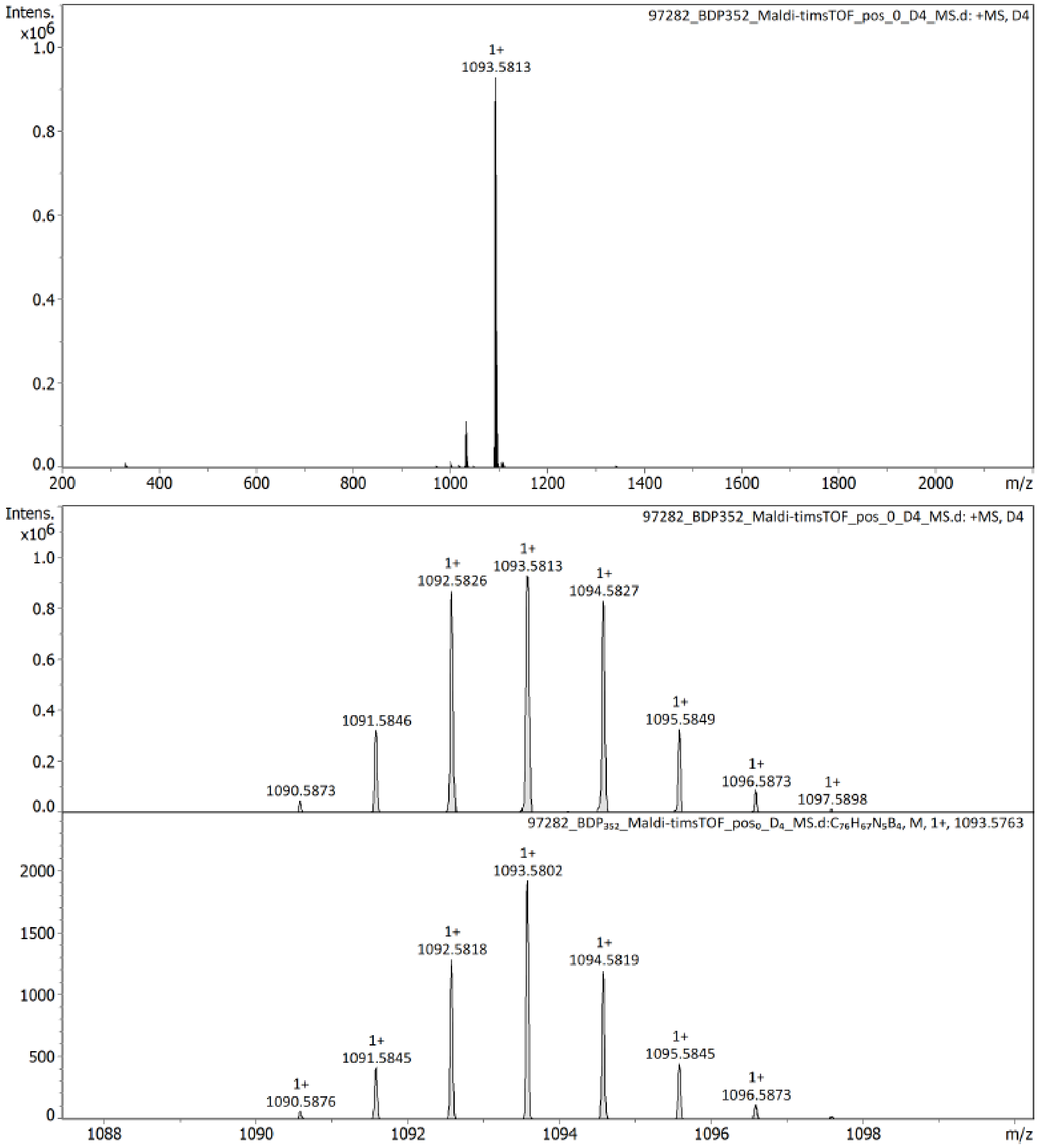

Figure S36. HRMS spectrum (MALDI) of molecule $\mathbf{1}_{\mathbf{NBNBN}}$.

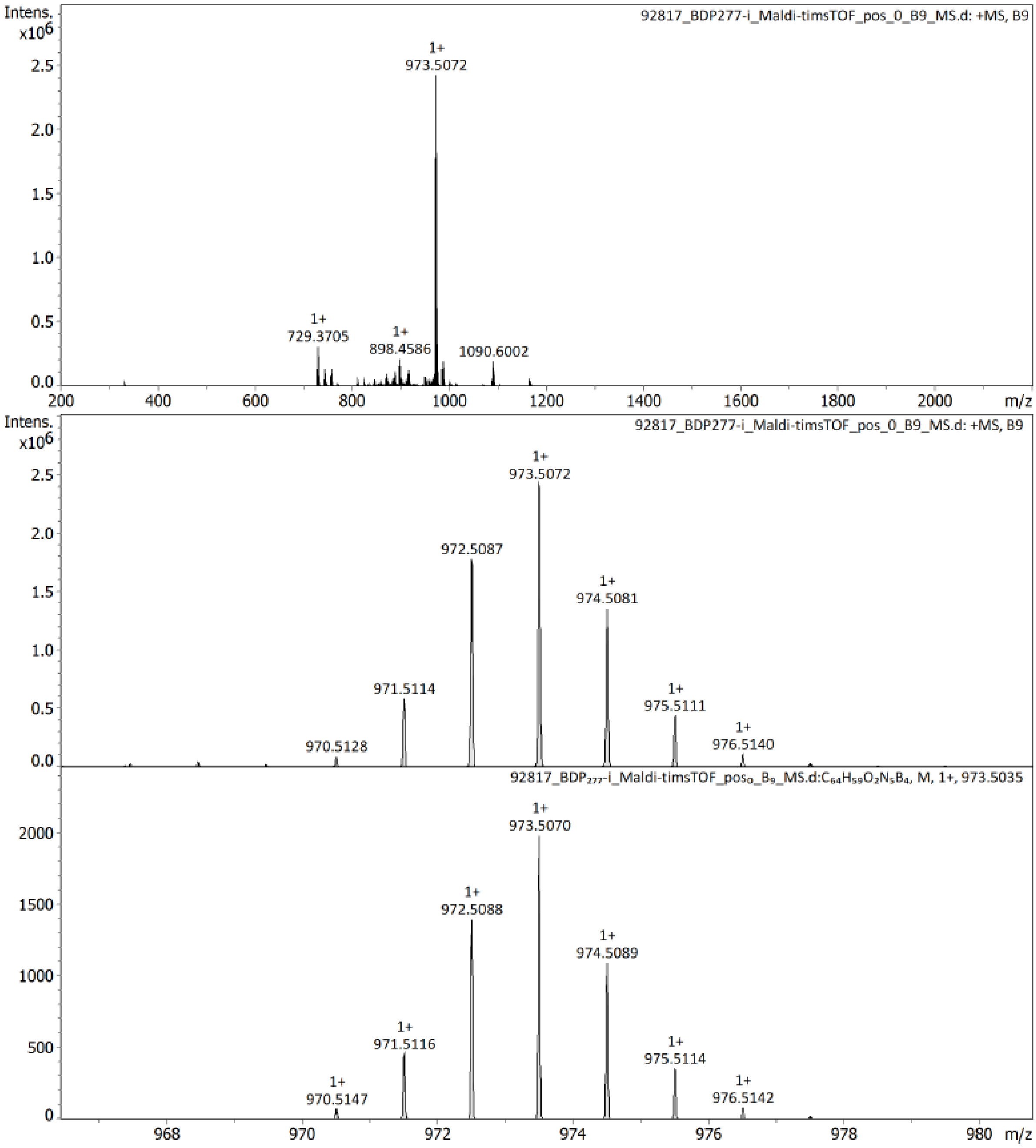

Figure S37. HRMS spectrum (MALDI) of molecule $\mathbf{2}_{\mathbf{NBNBN}}$.

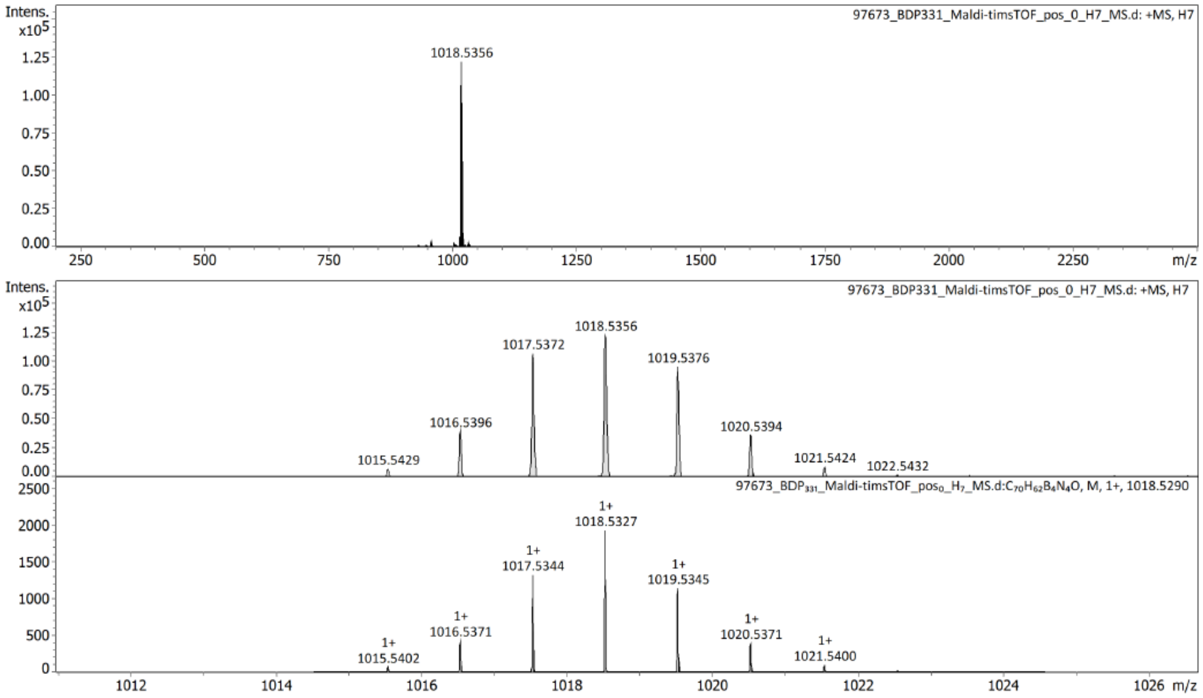

Figure S38. HRMS spectrum (MALDI) of molecule $\mathbf{1}_{\mathbf{NBOBN}}$.

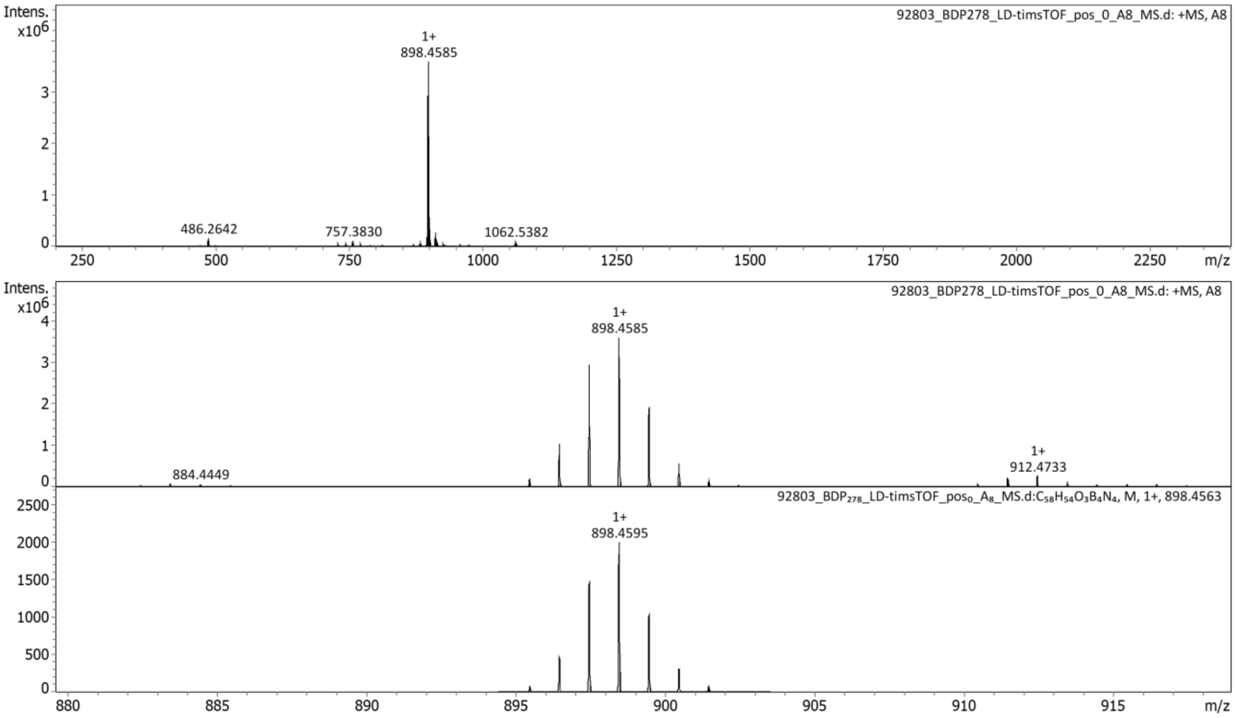

Figure S39. HRMS spectrum (LD) of molecule $\mathbf{2}_{\mathbf{NBOBN}}$.

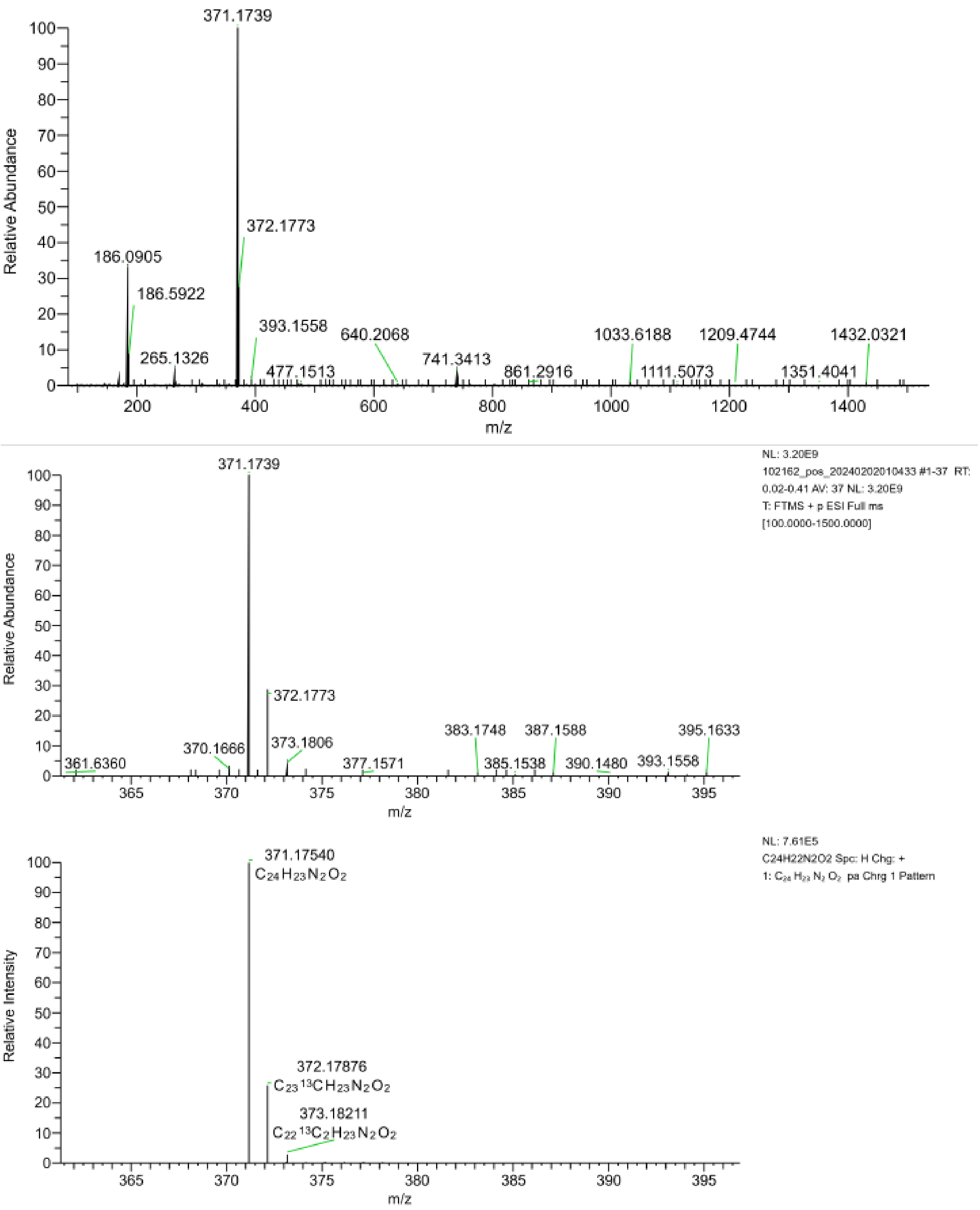

Figure S40. HRMS spectrum (ESI) of molecule **4**.

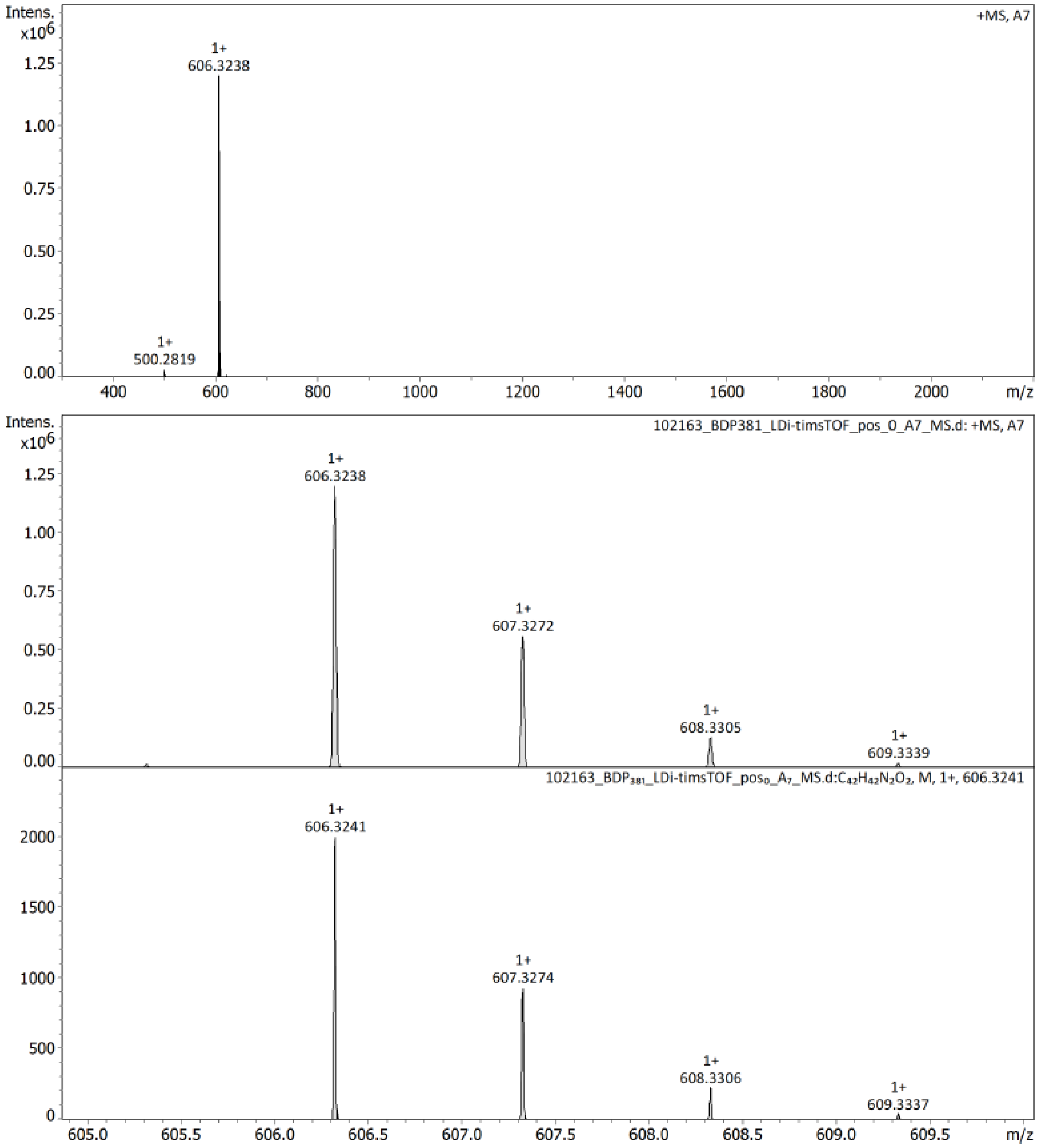

Figure S41. HRMS spectrum (LD) of molecule **5**.

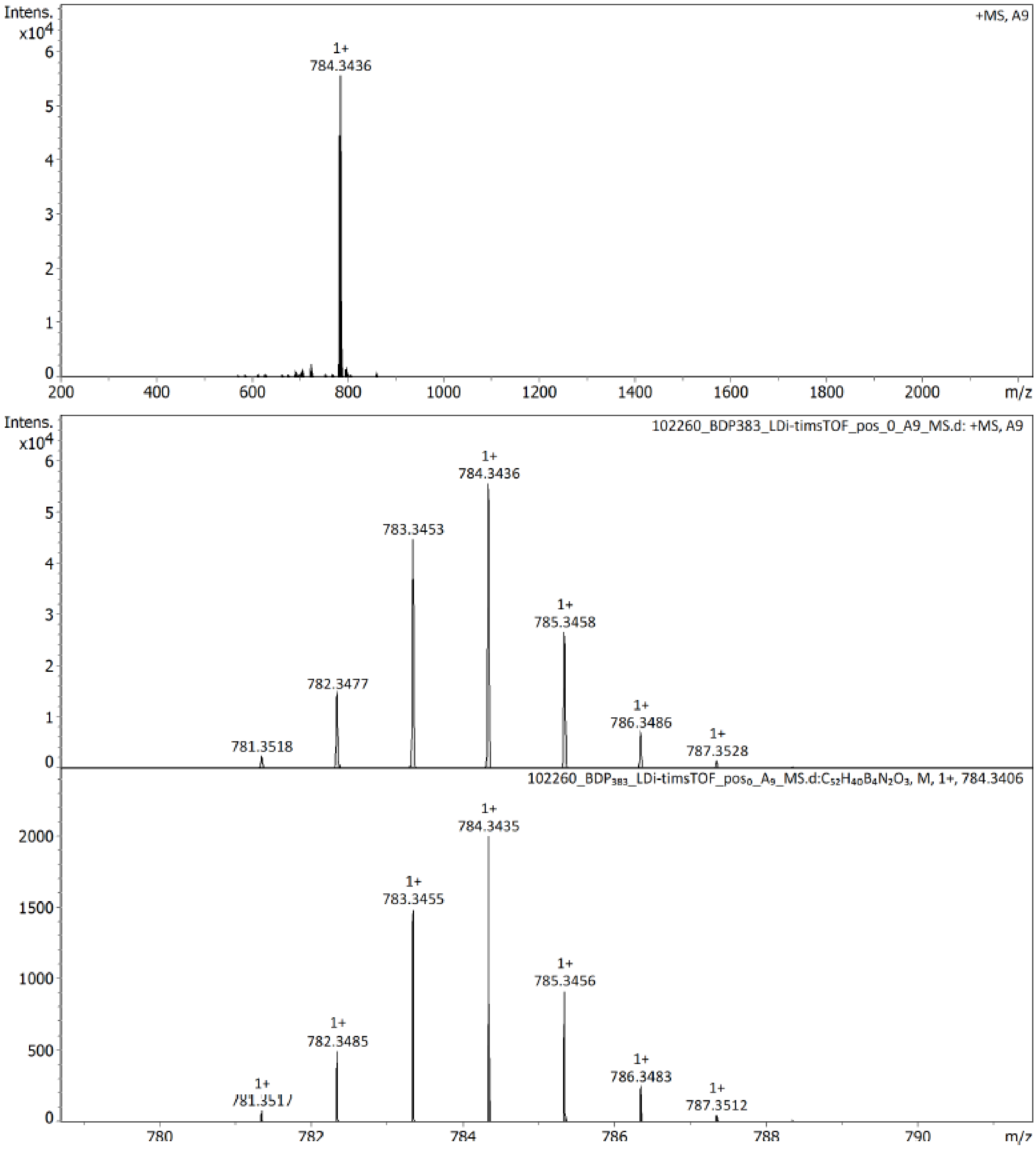

Figure S42. HRMS spectrum (LD) of molecule $\mathbf{1}_{\mathbf{OBOBO}}$.

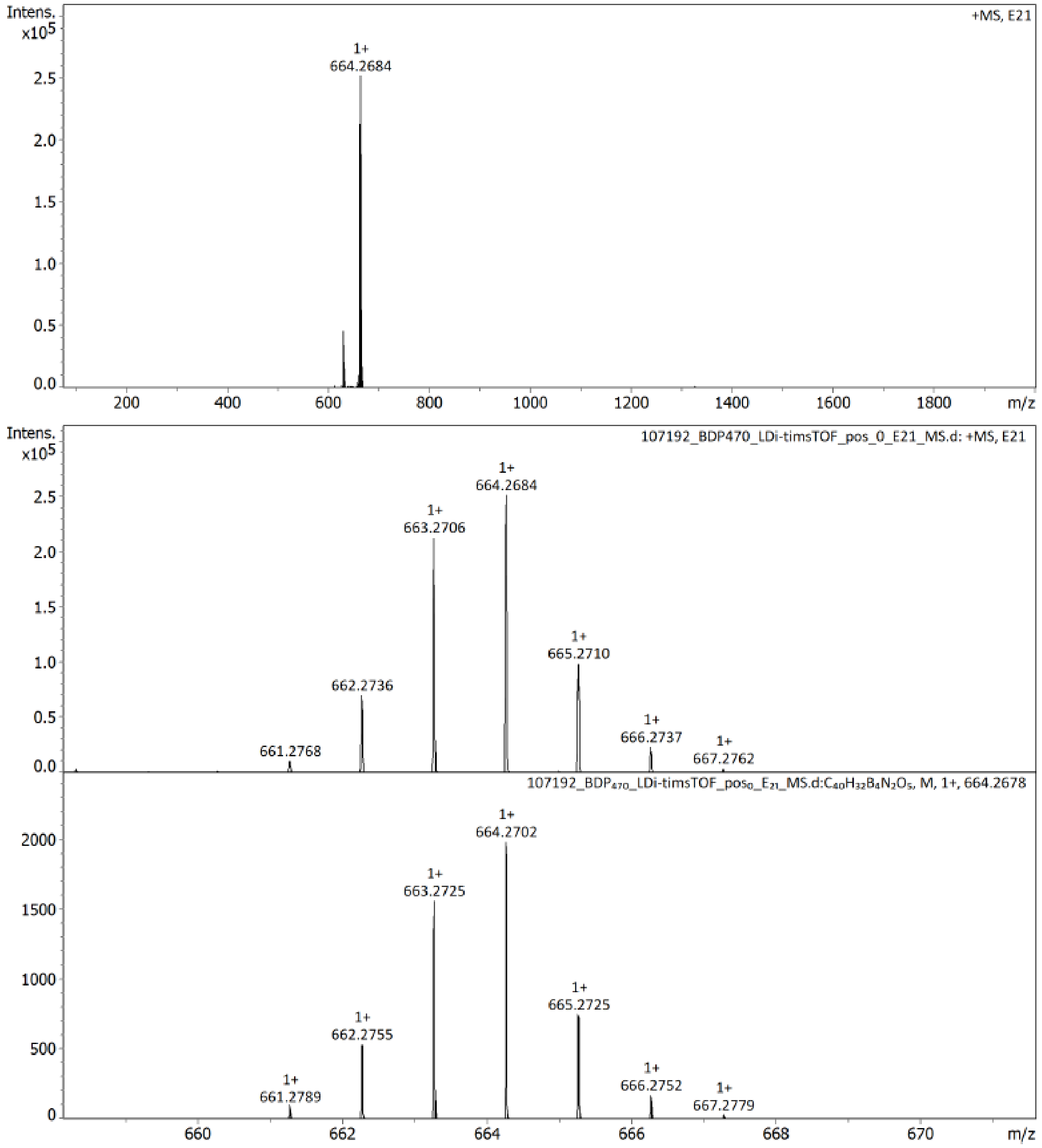

Figure S43. HRMS spectrum (LD) of molecule $\mathbf{2}_{\mathbf{OBOBO}}$.

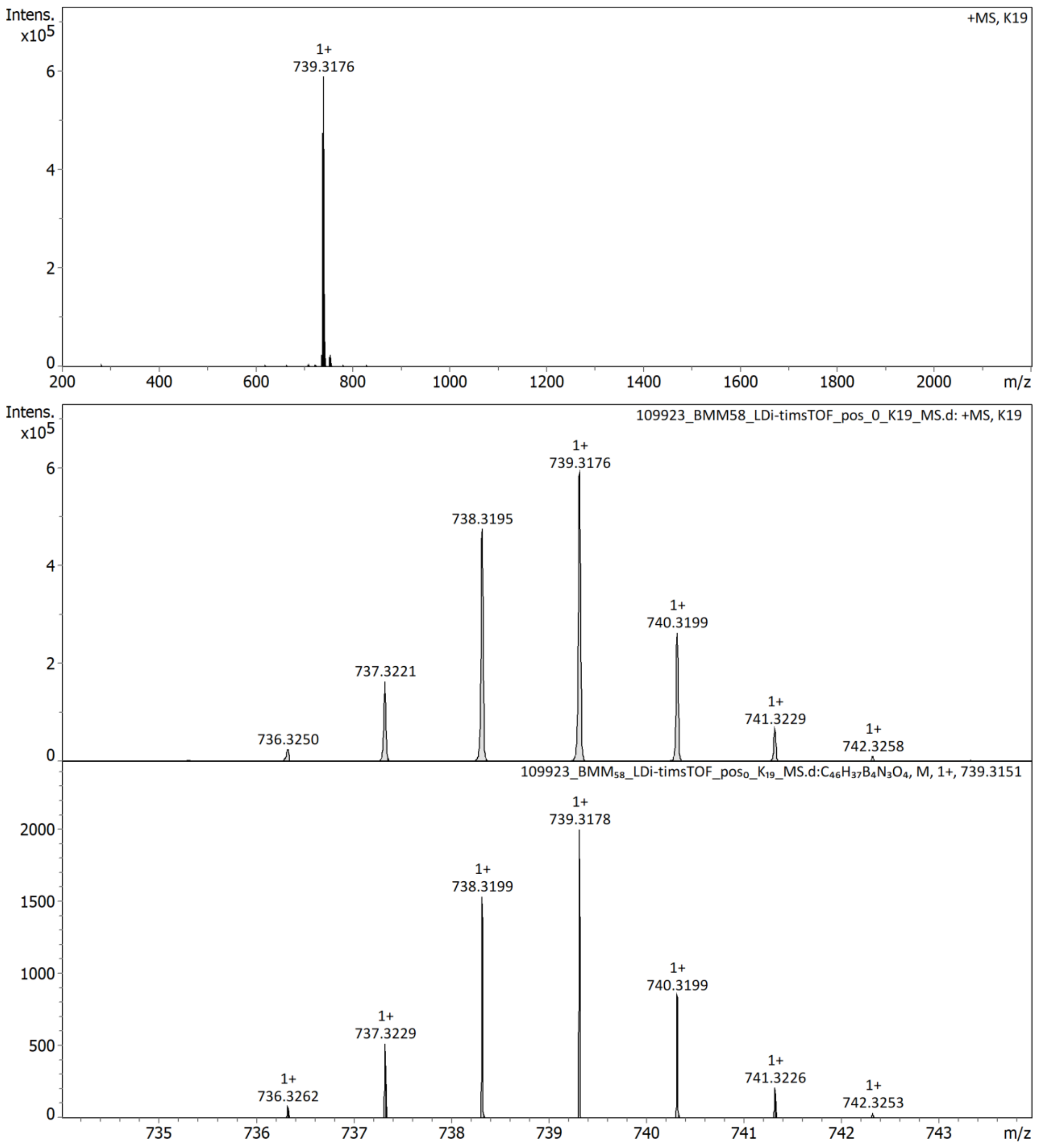

Figure S44. HRMS spectrum (LD) of molecule $\mathbf{2_{OBNBO}}$.

5. Photophysical characterization

Molecule 1_{NBNBN}

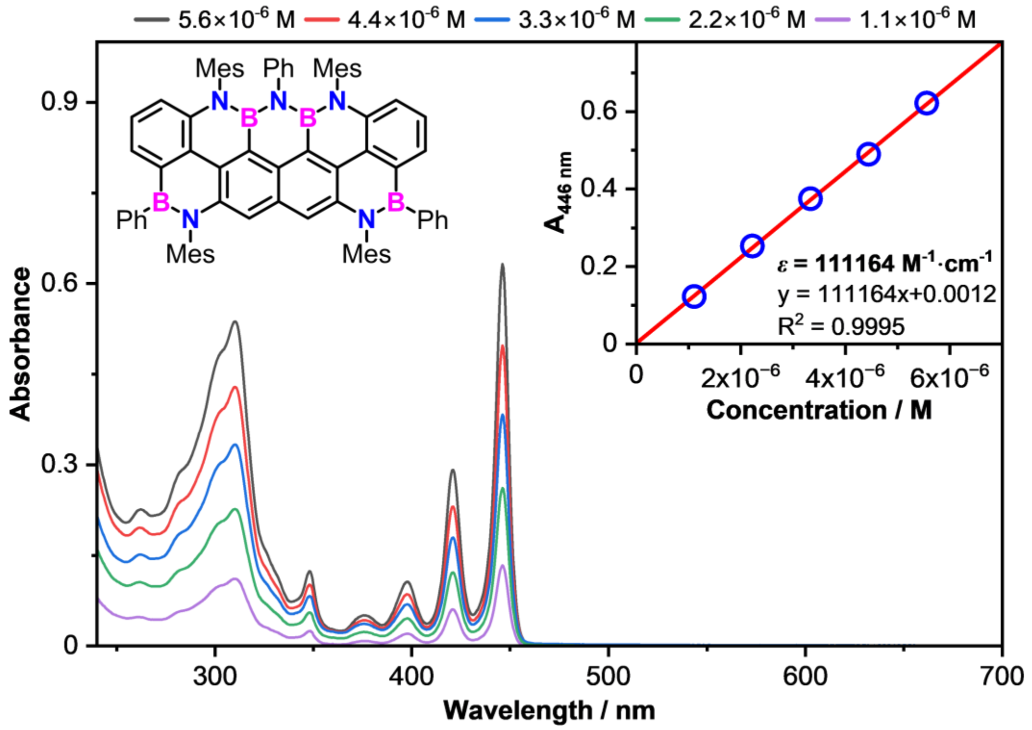

Figure S45. UV-vis absorption spectra of $\mathbf{1}_{\mathbf{NBNBN}}$ (1.1×10^{-6} – 5.6×10^{-6} M) in 2-MeTHF. Inset: determination of the molar attenuation coefficient (λ_{abs} = 446 nm).

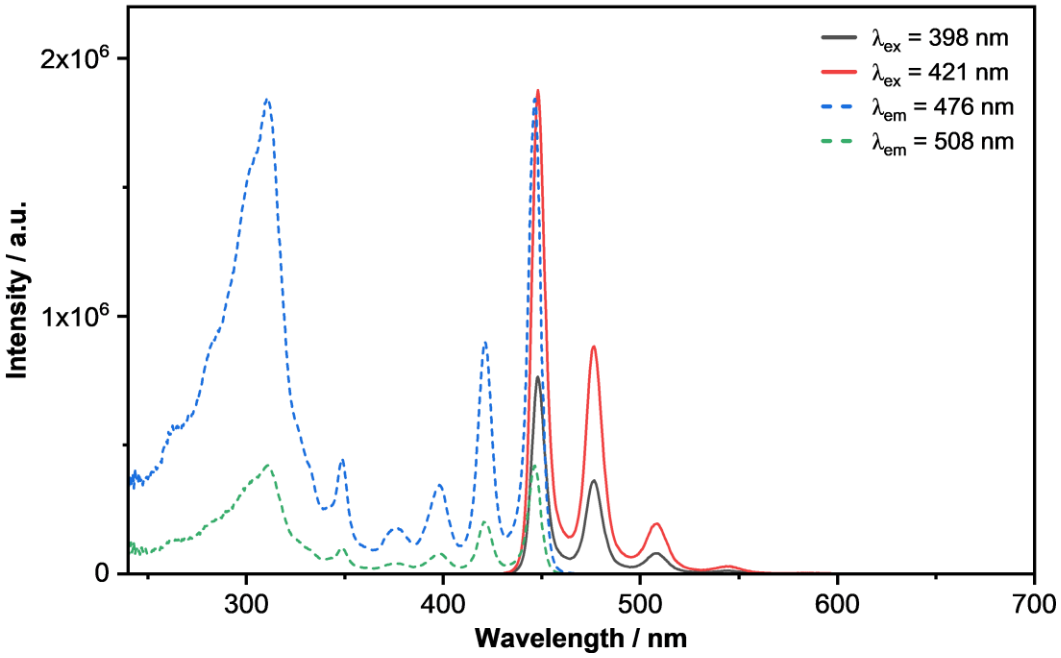

Figure S46. Steady-state excitation (dashed) and emission (solid) spectra of $\mathbf{1}_{\mathbf{NBNBN}}$ (1.1×10^{-6} M) in 2-MeTHF.

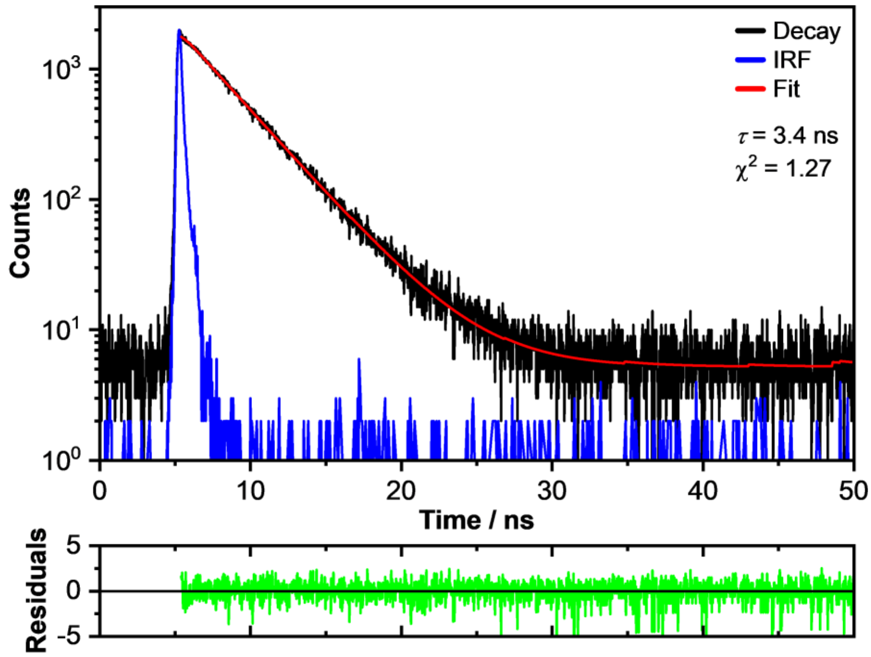

Figure S47. Fluorescence lifetime decay and residual analysis (λ_{ex} = 405.6 nm, λ_{em} = 448 nm) of $\mathbf{1}_{\mathbf{NBNBN}}$ (1.1×10^{-6} M) in 2-MeTHF.

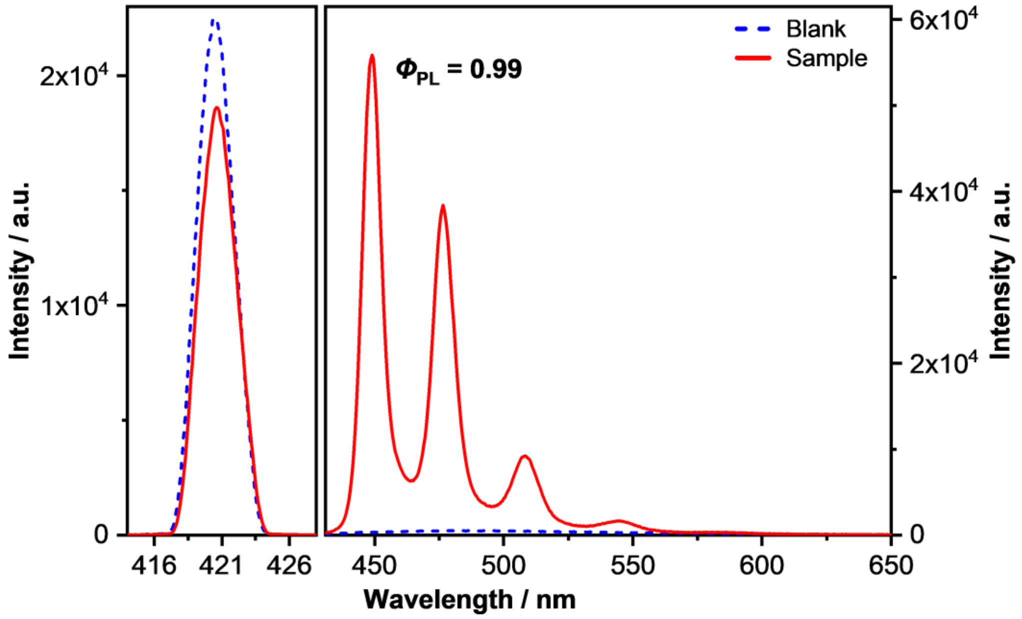

Figure S48. Excitation scatter region (left) and emission spectra (right, λ_{ex} = 421 nm) used to calculate the absolute quantum yield of $\mathbf{1}_{\mathbf{NBNBN}}$ (8.8×10^{-7} M) in 2-MeTHF.

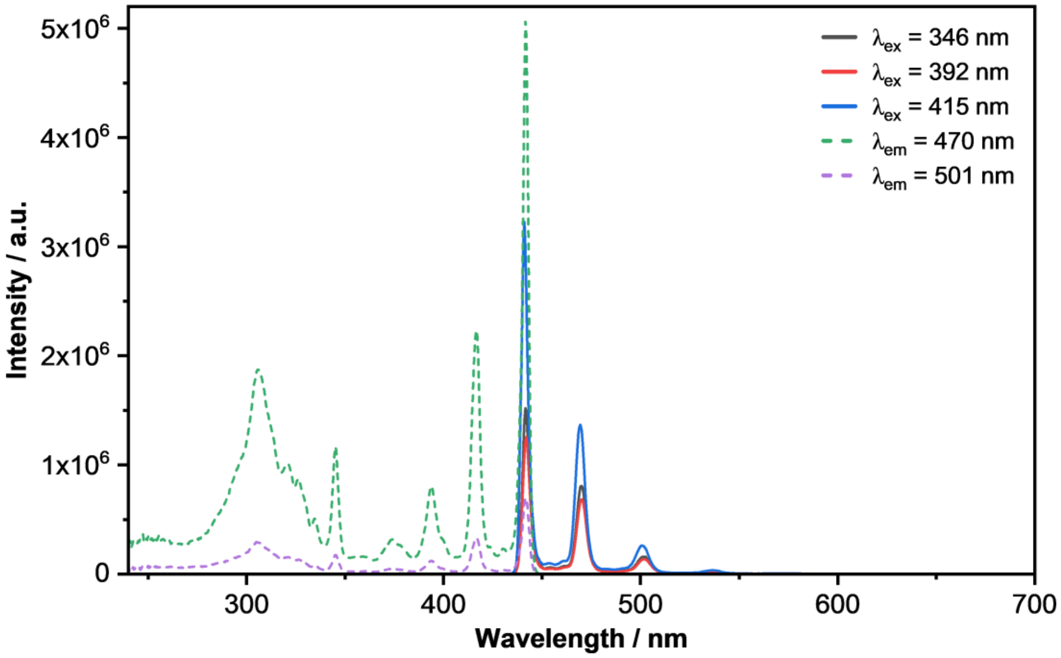

Figure S49. Steady-state excitation (dashed) and emission (solid) spectra of $\mathbf{1}_{\mathbf{NBNBN}}$ (1.1×10^{-6} M) in 2-MeTHF at 77 K.

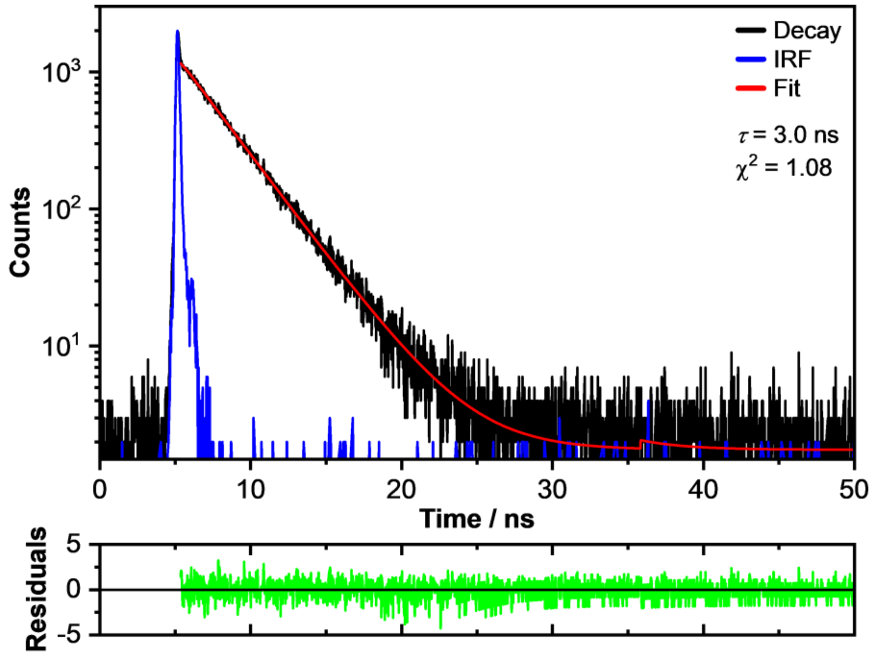

Figure S50. Fluorescence lifetime decay and residual analysis (λ_{ex} = 405.6 nm, λ_{em} = 448 nm) of $\mathbf{1}_{\mathbf{NBNBN}}$ (1.1×10^{-6} M) in 2-MeTHF at 77 K.

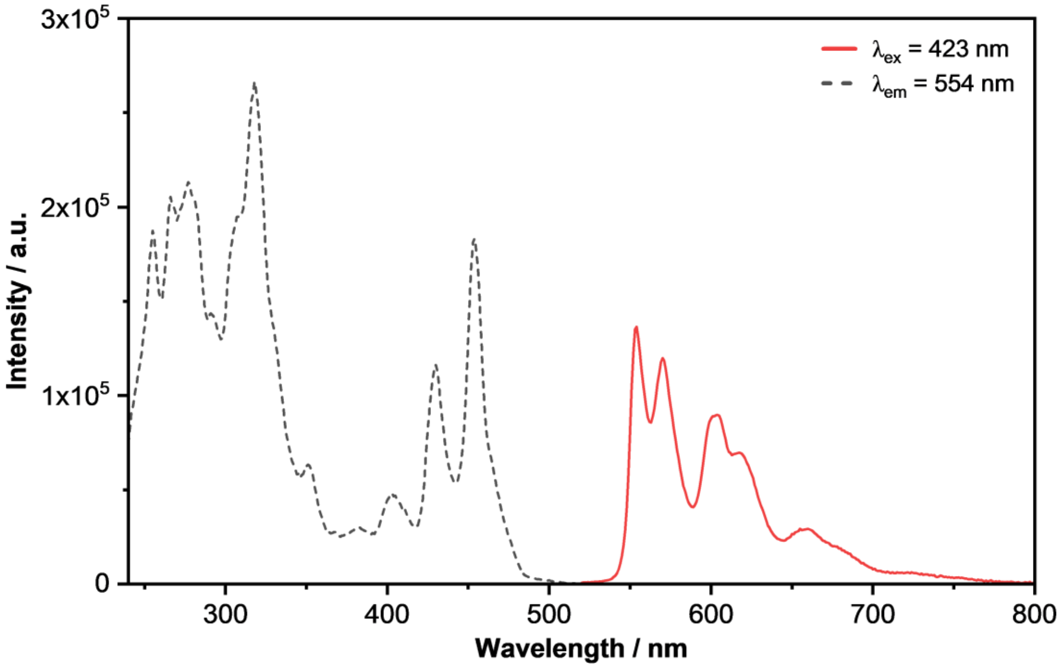

Figure S51. Transient excitation (dashed) and emission (solid) spectra of **1**$_{\mathbf{NBNBN}}$ (1.1×10^{-6} M) in 2-MeTHF at 77 K. Gate delay: 200 µs.

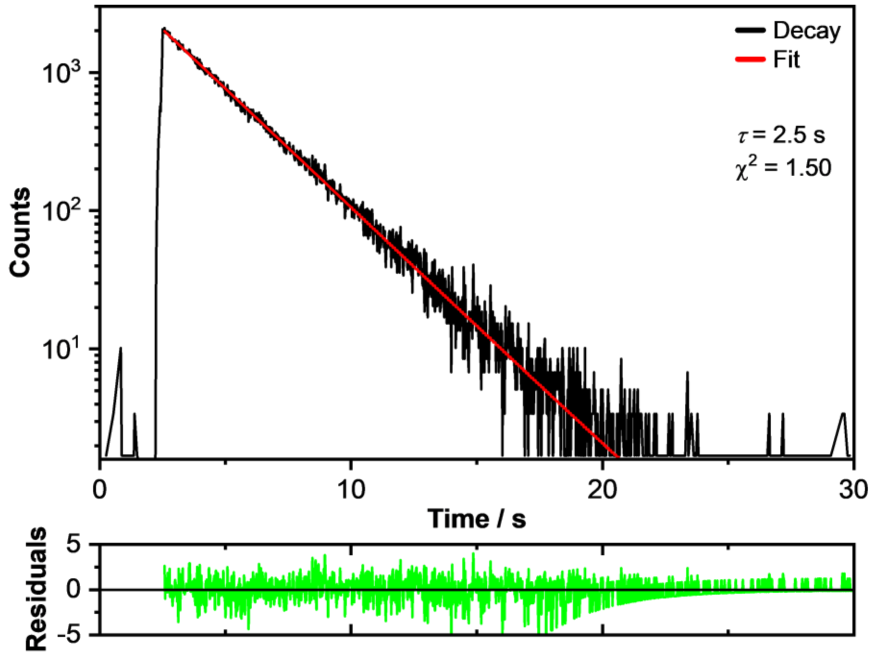

Figure S52. Phosphorescence lifetime decay and residual analysis (λ_{ex} = 421 nm, λ_{em} = 555 nm) of **1**$_{\mathbf{NBNBN}}$ (1.1×10^{-6} M) in 2-MeTHF at 77 K.

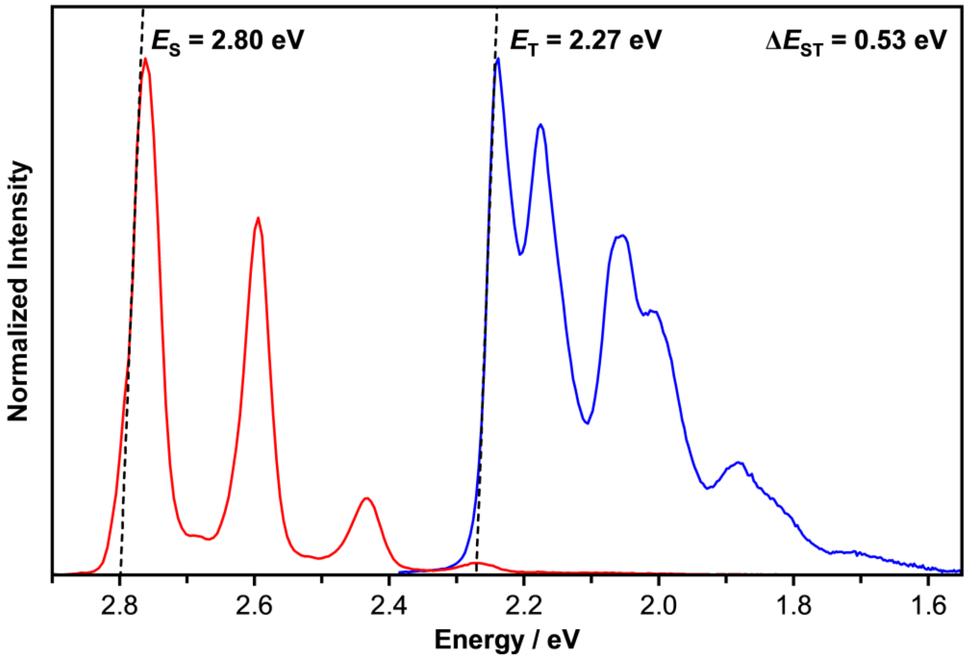

Figure S53. Determination of the singlet-triplet gap of $\mathbf{1}_{\mathbf{NBNBN}}$ ($1.1{\times}10^{-6}$ M) in 2-MeTHF at 77 K, using the onset of the stead-state fluorescence (red, λ_{ex} = 420 nm) and the phosphorescence (blue, λ_{ex} = 423 nm, gate delay: 200 µs) emission spectra in 2-MeTHF at 77 K.

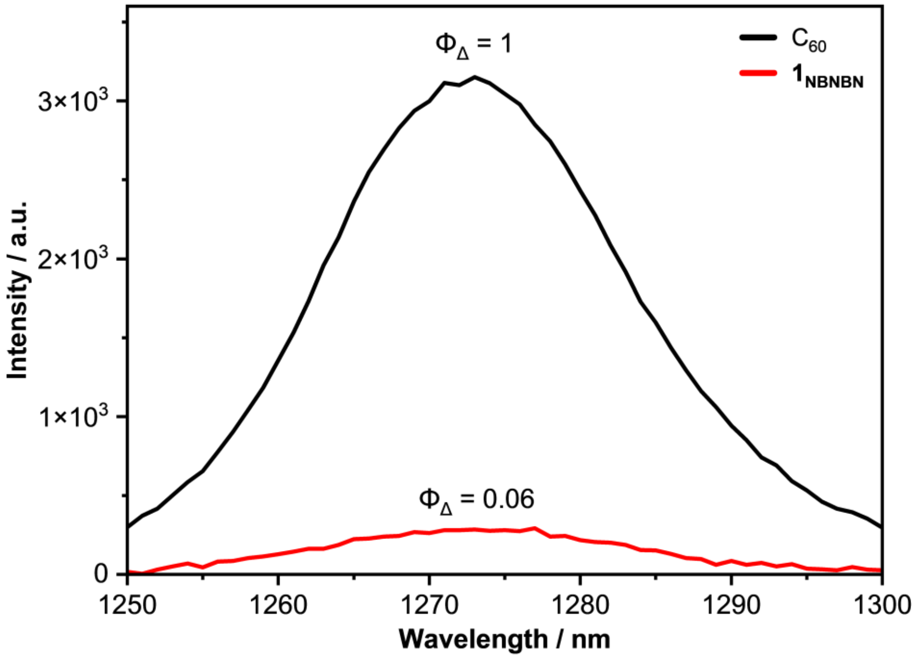

Figure S54. Singlet oxygen phosphorescence emission sensitized by C_{60} (black, $8.9{\times}10^{-6}$ M, λ_{ex} = 375 nm) and $\mathbf{1}_{\mathbf{NBNBN}}$ (red, $7.7{\times}10^{-7}$ M, λ_{ex} = 448 nm) in $CHCl_3$.

Molecule 1_{NBOBN}

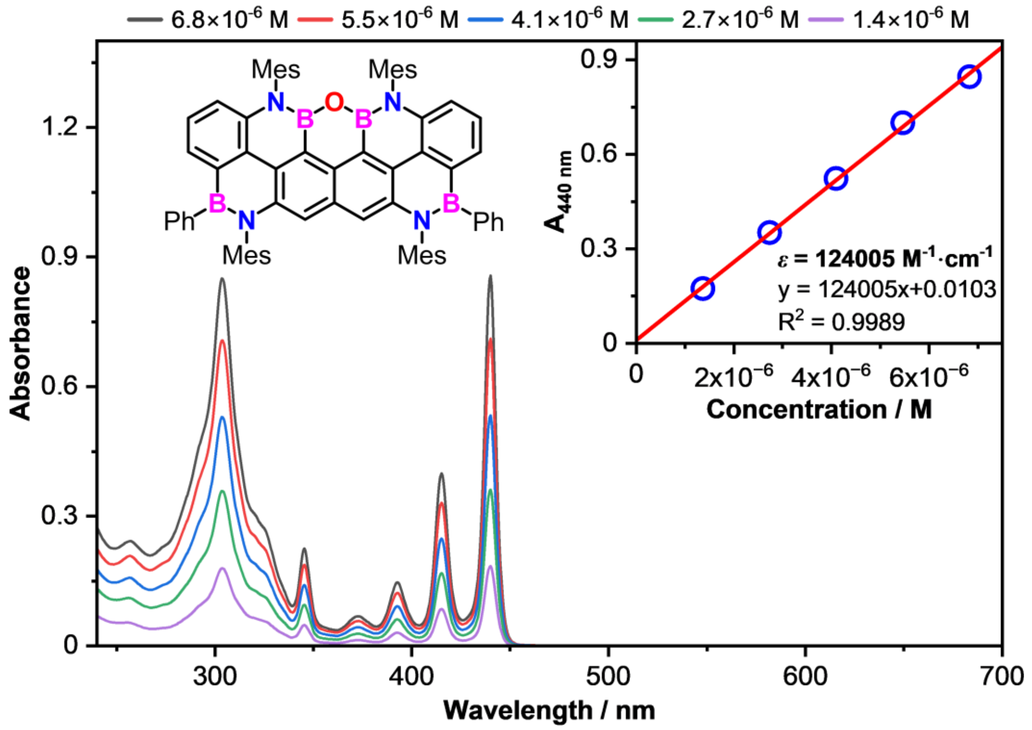

Figure S55. UV-vis absorption spectra of $\mathbf{1}_{\mathbf{NBOBN}}$ ($1.4\times10^{-6} – 6.8\times10^{-6}$ M) in 2-MeTHF. Inset: determination of the molar attenuation coefficient (λ_{abs} = 440 nm).

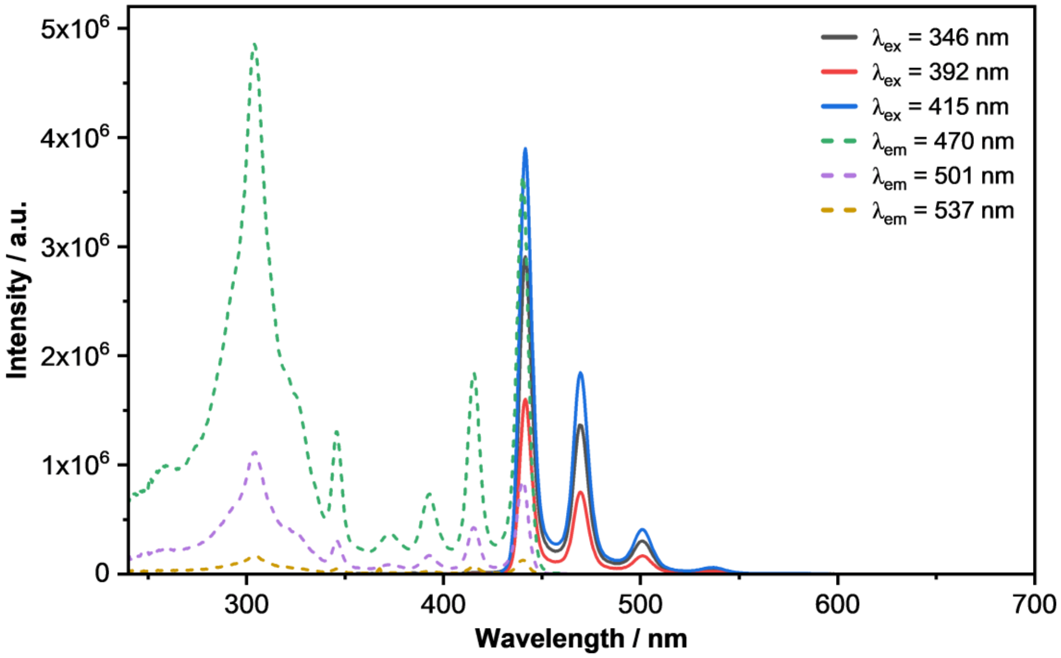

Figure S56. Steady-state excitation (dashed) and emission (solid) spectra of $\mathbf{1}_{\mathbf{NBOBN}}$ (8.9×10^{-7} M) in 2-MeTHF.

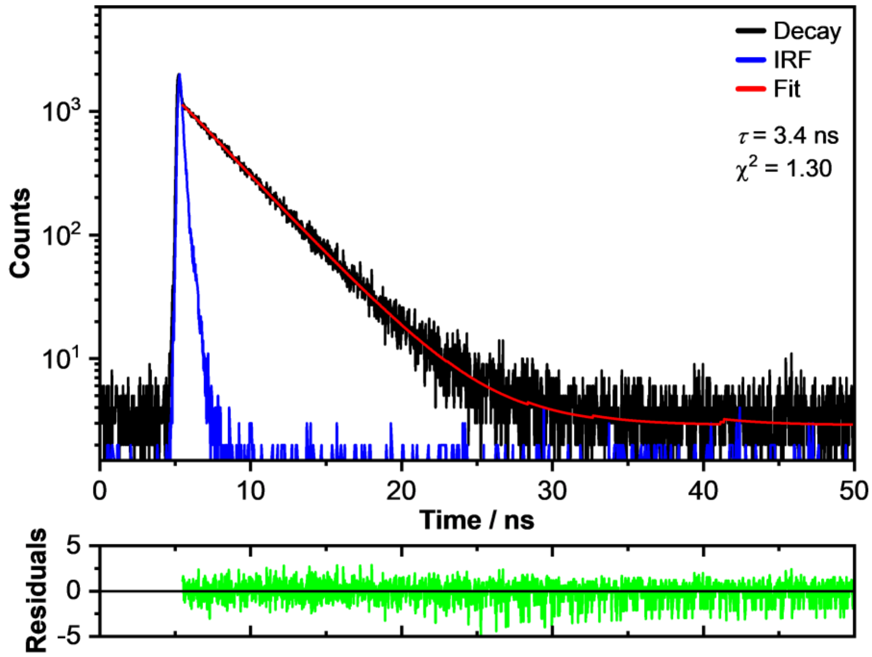

Figure S57. Fluorescence lifetime decay and residual analysis (λ_{ex} = 405.6 nm, λ_{em} = 442 nm) of $\mathbf{1_{NBOBN}}$ (8.9×10^{-7} M) in 2-MeTHF.

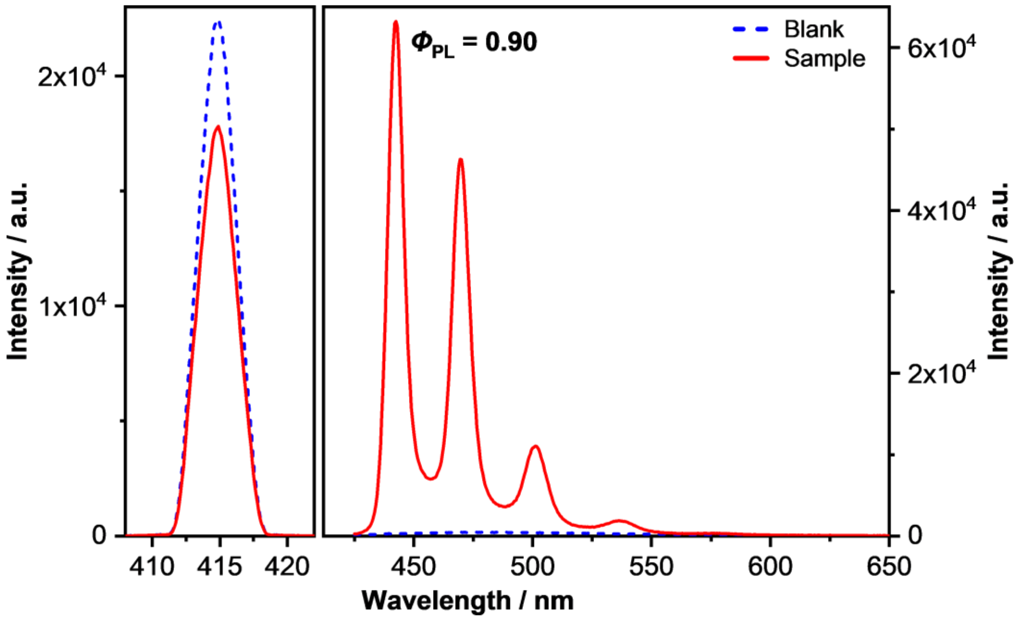

Figure S58. Excitation scatter region (left) and emission spectra (right, λ_{ex} = 415 nm) used to calculate the absolute quantum yield of $\mathbf{1_{NBOBN}}$ (9.5×10^{-7} M) in 2-MeTHF.

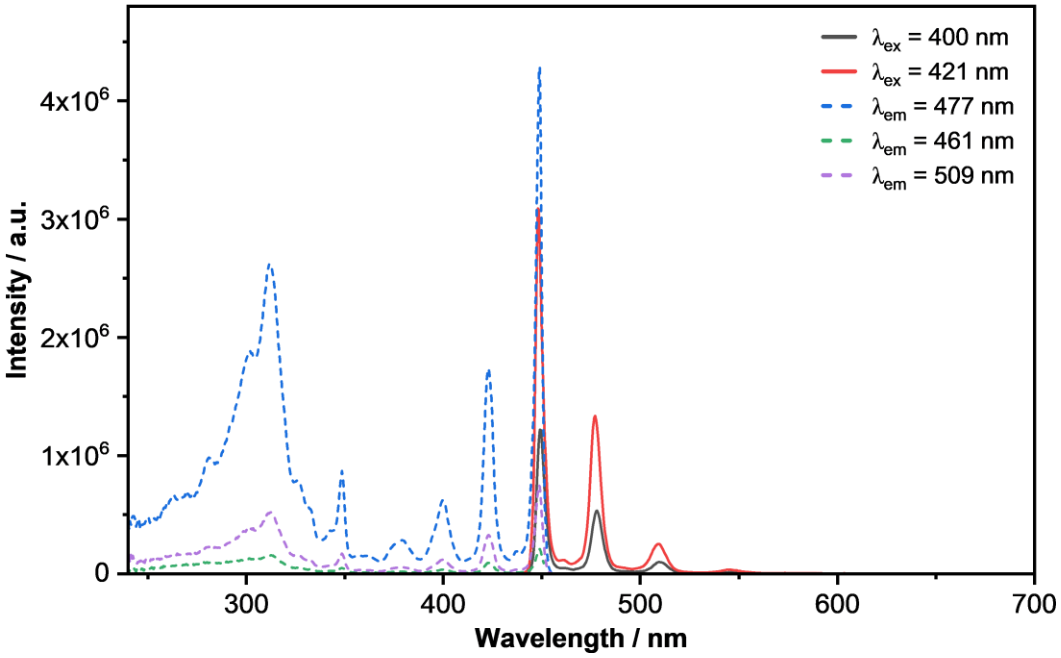

Figure S59. Steady-state excitation (dashed) and emission (solid) spectra of $\mathbf{1}_{\mathbf{NBOBN}}$ (8.9×10^{-7} M) in 2-MeTHF at 77 K.

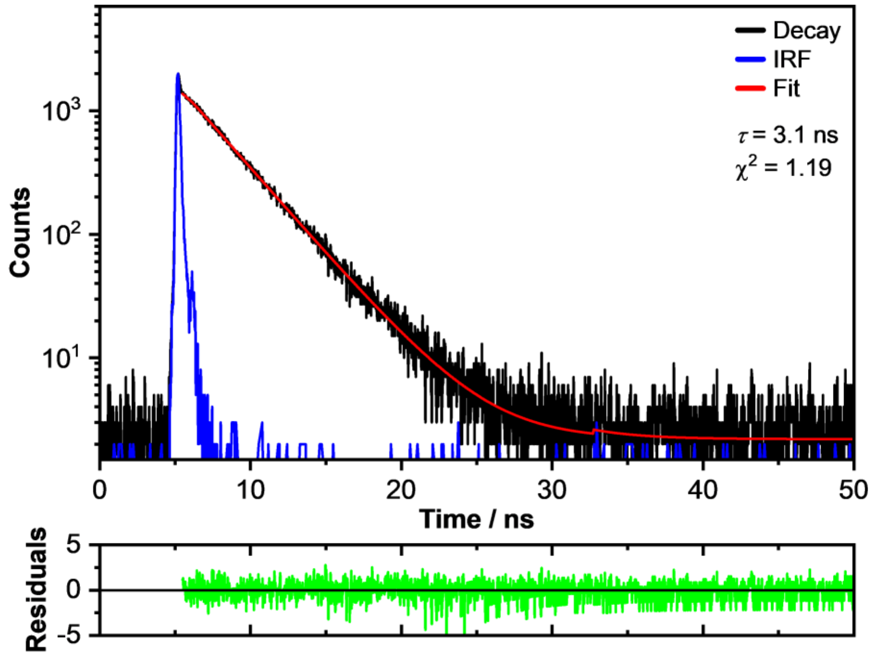

Figure S60. Fluorescence lifetime decay and residual analysis (λ_{ex} = 405.6 nm, λ_{em} = 441 nm) of $\mathbf{1}_{\mathbf{NBOBN}}$ (8.9×10^{-7} M) in 2-MeTHF at 77 K.

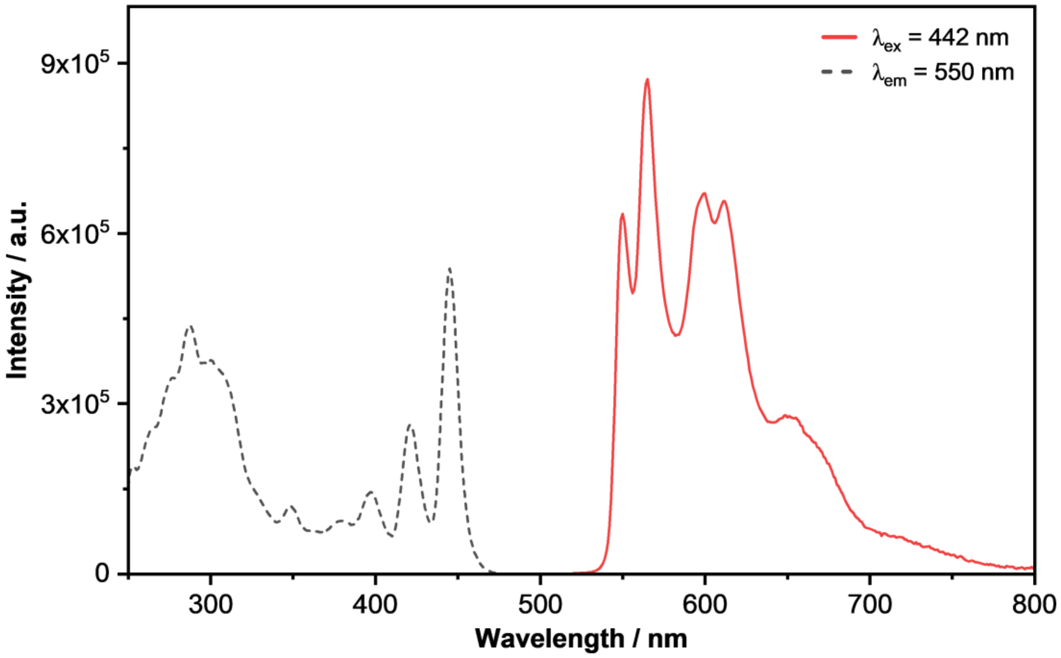

Figure S61. Transient excitation (dashed) and emission (solid) spectra of **1_{NBOBN}** (8.9×10^{-7} M) in 2-MeTHF at 77 K. Gate delay: 200 µs.

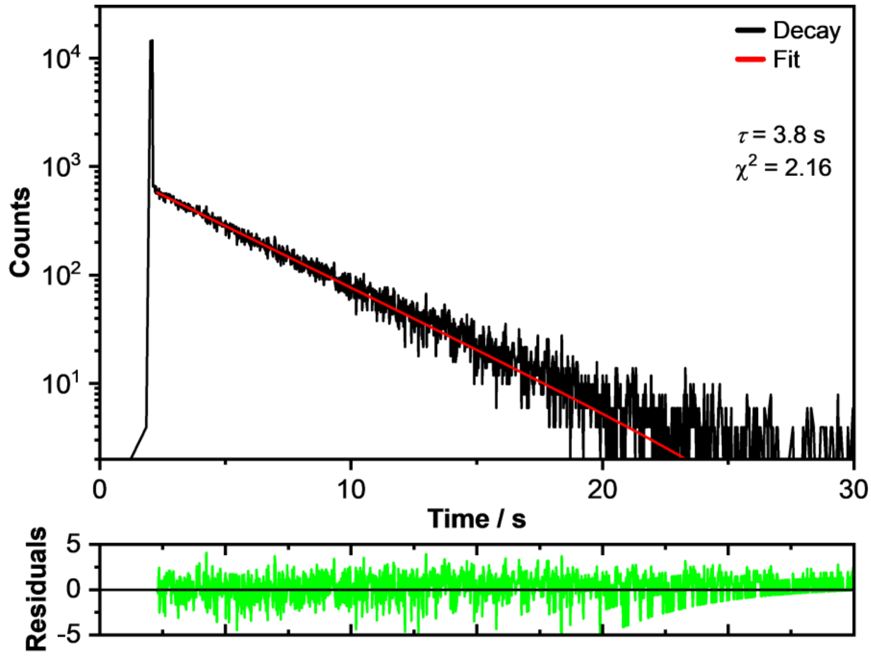

Figure S62. Phosphorescence lifetime decay and residual analysis (λ_{ex} = 415 nm, λ_{em} = 550 nm) of **1_{NBOBN}** (8.9×10^{-7} M) in 2-MeTHF at 77 K.

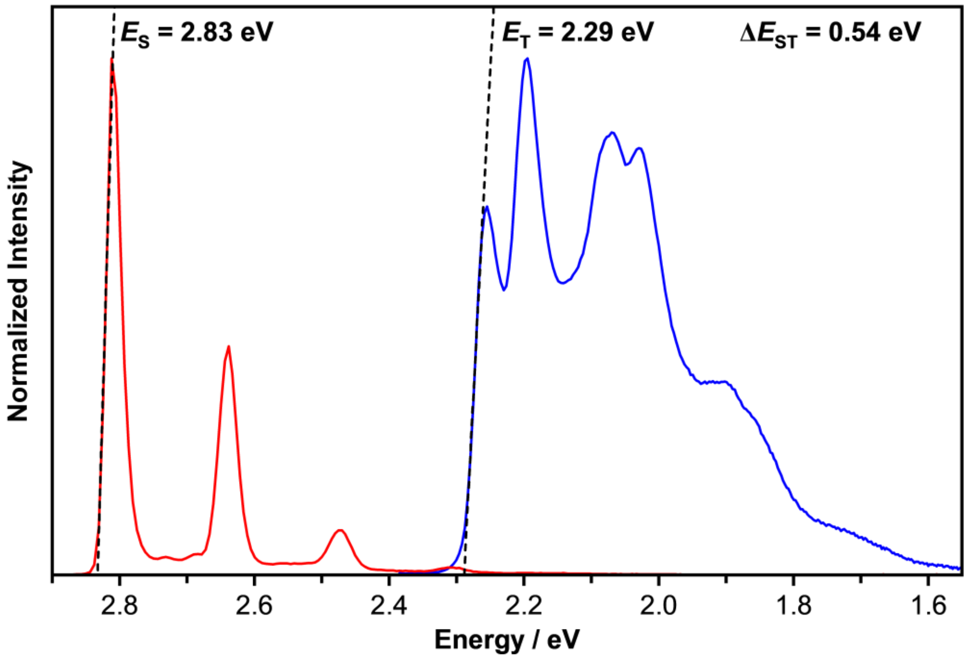

Figure S63. Determination of the singlet-triplet gap of **1**$_{\mathbf{NBOBN}}$ (8.9×10^{-7} M) in 2-MeTHF at 77 K, using the onset of the stead-state fluorescence (red, λ_{ex} = 415 nm) and the phosphorescence (blue, λ_{ex} = 442 nm, gate delay: 200 µs) emission spectra in 2-MeTHF at 77 K.

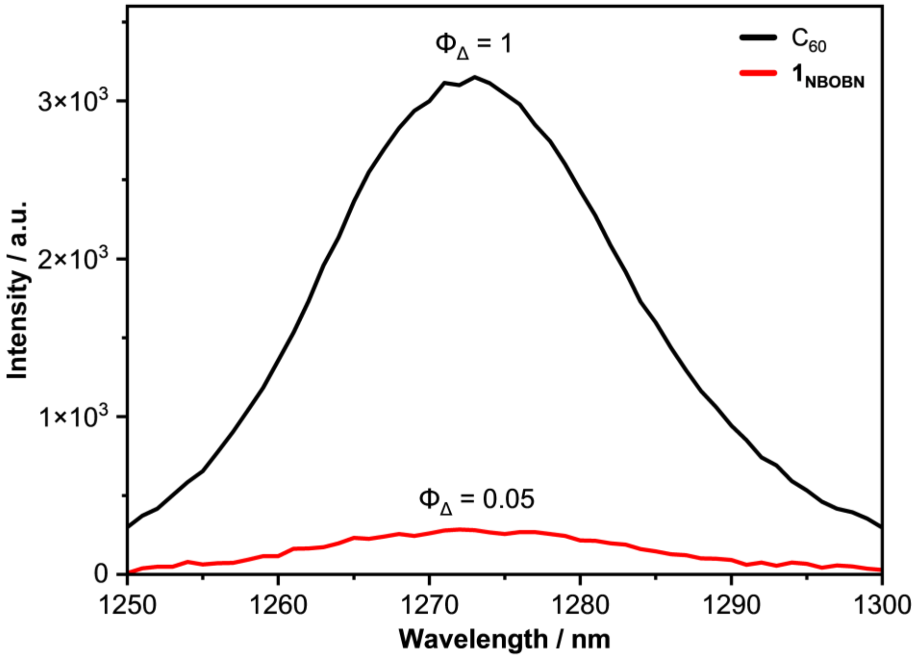

Figure S64. Singlet oxygen phosphorescence emission sensitized by C_{60} (black, 8.9×10^{-6} M, λ_{ex} = 375 nm) and **1**$_{\mathbf{NBOBN}}$ (red, 8.9×10^{-7} M, λ_{ex} = 441 nm) in $CHCl_3$.

Molecule 1_{OBOBO}

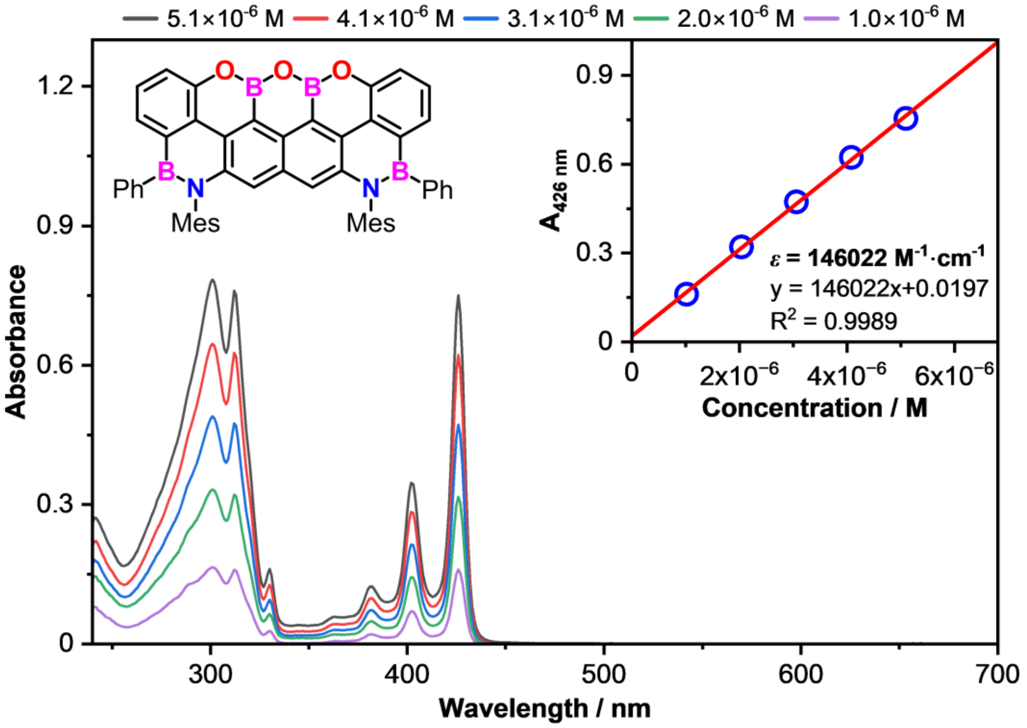

Figure S65. UV-vis absorption spectra of $\mathbf{1}_{\mathbf{OBOBO}}$ ($1.0\times10^{-6} – 5.1\times10^{-6}$ M) in 2-MeTHF. Inset: determination of the molar attenuation coefficient (λ_{abs} = 426 nm).

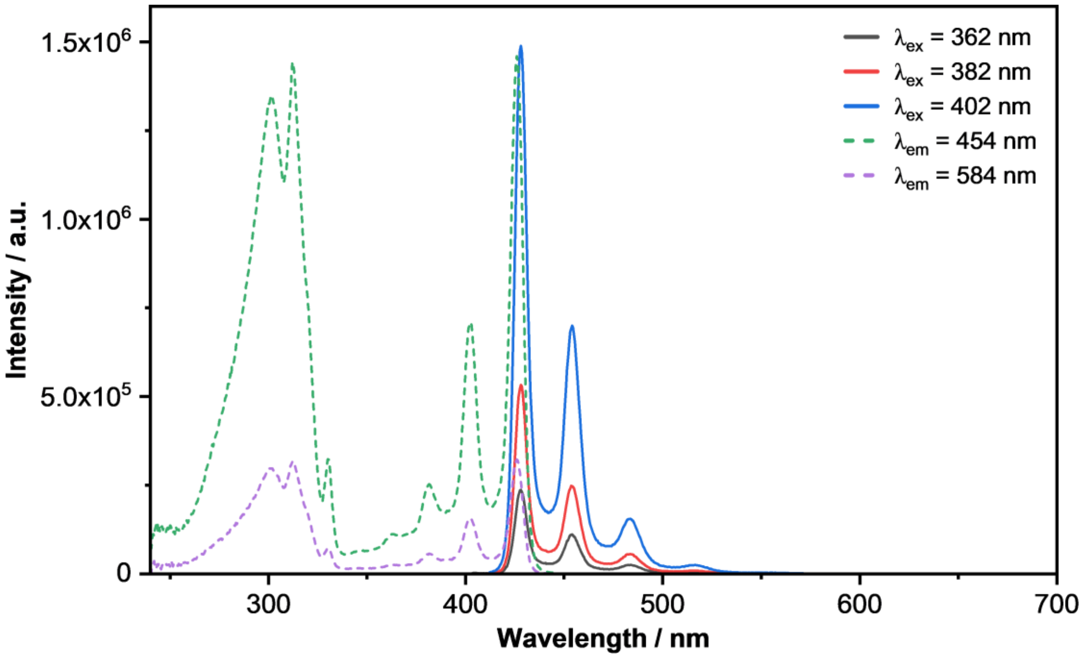

Figure S66. Steady-state excitation (dashed) and emission (solid) spectra of $\mathbf{1}_{\mathbf{OBOBO}}$ (6.9×10^{-7} M) in 2-MeTHF.

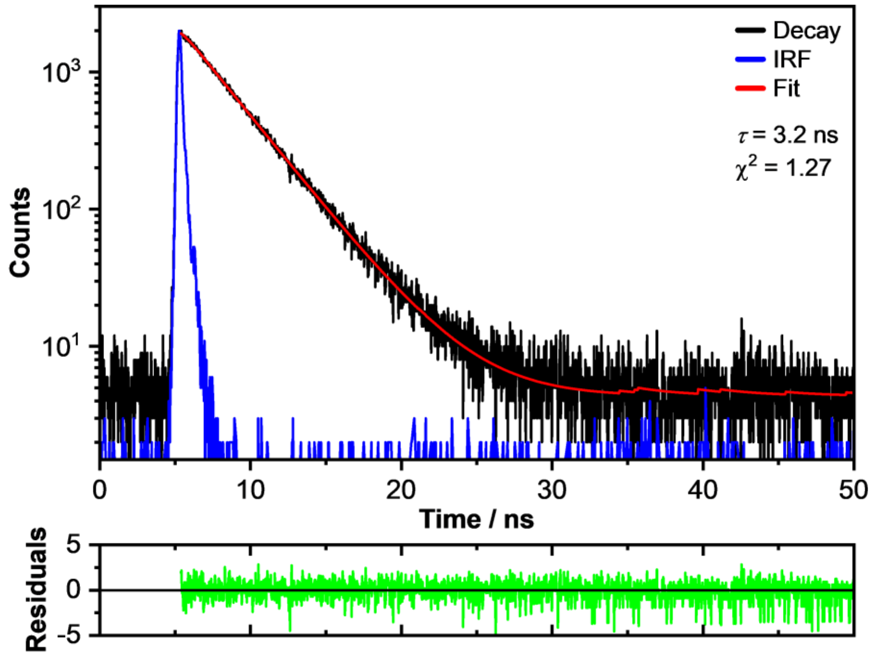

Figure S67. Fluorescence lifetime decay and residual analysis (λ_{ex} = 405.6 nm, λ_{em} = 428 nm) of $\mathbf{1}_{\mathbf{OBOBO}}$ (6.9×10^{-7} M) in 2-MeTHF.

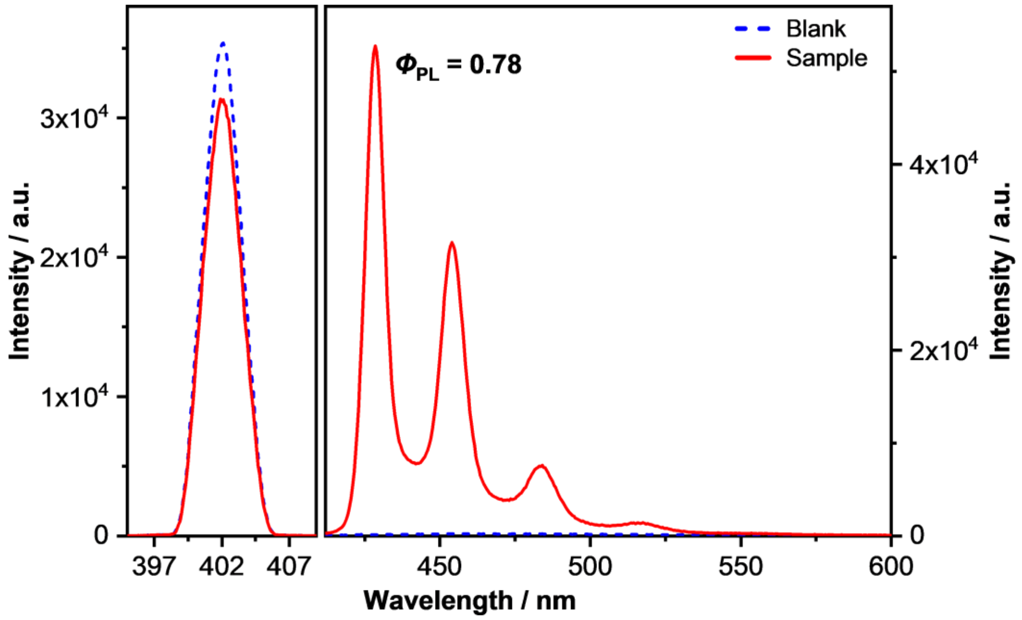

Figure S68. Excitation scatter region (left) and emission spectra (right, λ_{ex} = 402 nm) used to calculate the absolute quantum yield of $\mathbf{1}_{\mathbf{OBOBO}}$ (5.5×10^{-7} M) in 2-MeTHF.

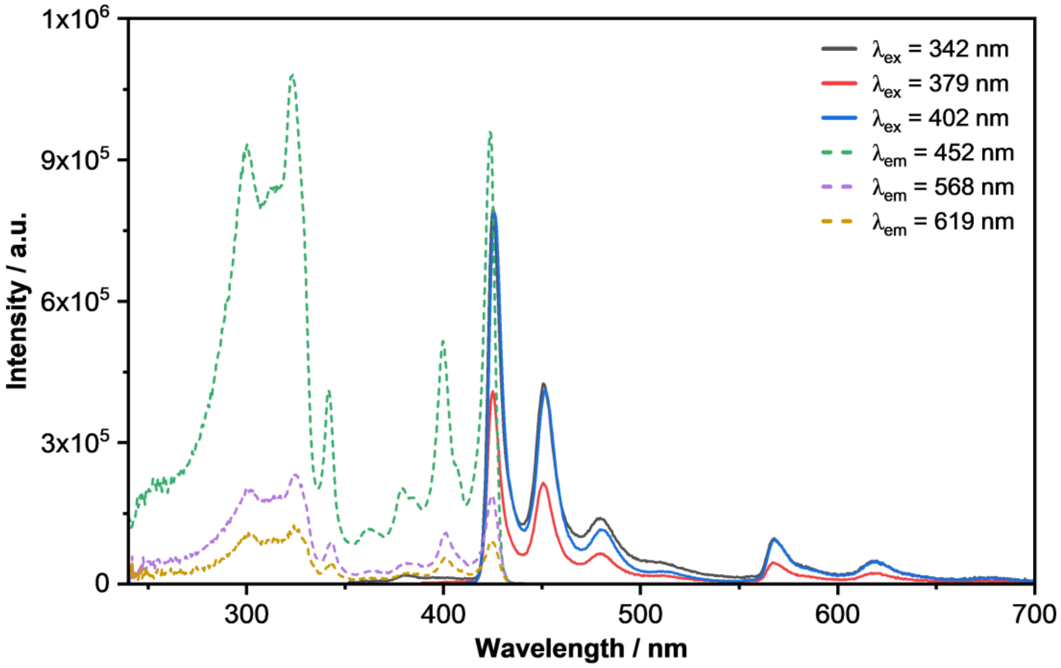

Figure S69. Steady-state excitation (dashed) and emission (solid) spectra of $\mathbf{1}_{\mathbf{OBOBO}}$ (6.9×10^{-7} M) in 2-MeTHF at 77 K.

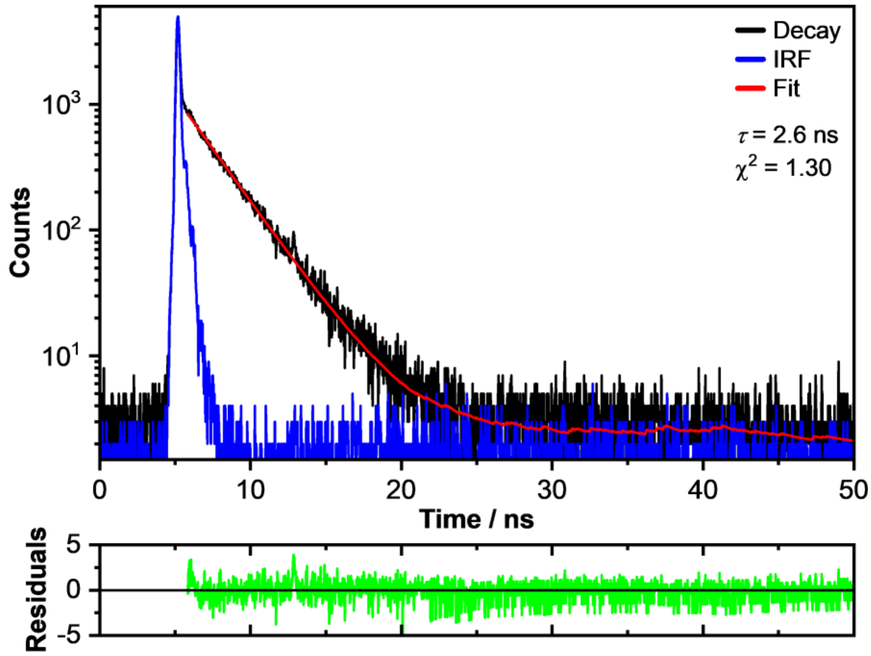

Figure S70. Fluorescence lifetime decay and residual analysis (λ_{ex} = 405.6 nm, λ_{em} = 428 nm) of $\mathbf{1}_{\mathbf{OBOBO}}$ (6.9×10^{-7} M) in 2-MeTHF at 77 K.

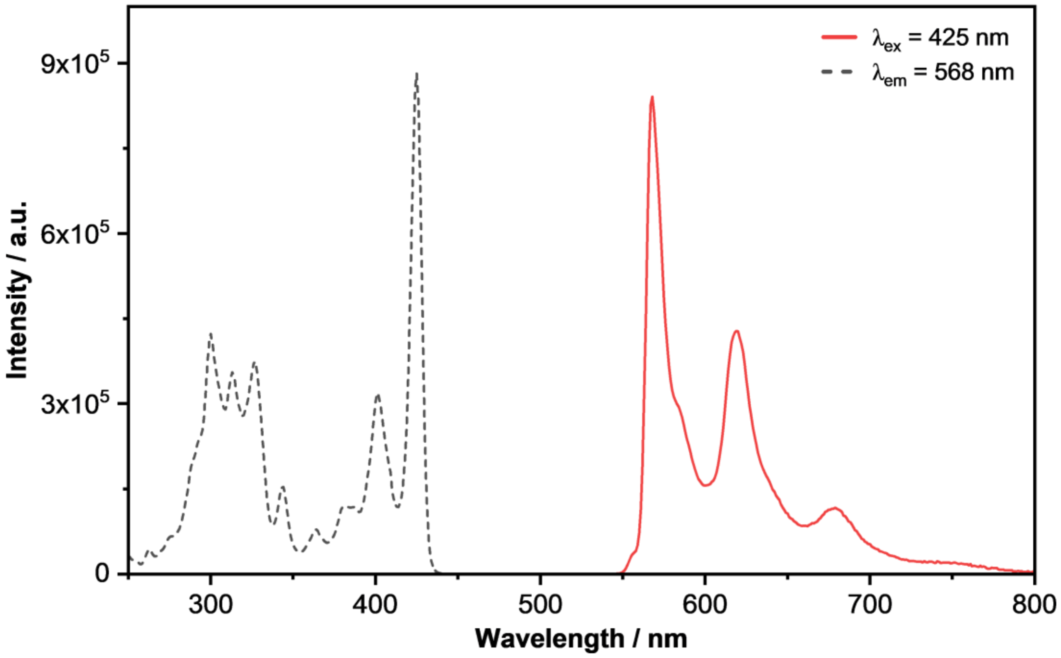

Figure S71. Transient excitation (dashed) and emission (solid) spectra of **1**$_{\mathbf{OBOBO}}$ (6.9×10^{-7} M) in 2-MeTHF at 77 K. Gate delay: 1000 µs.

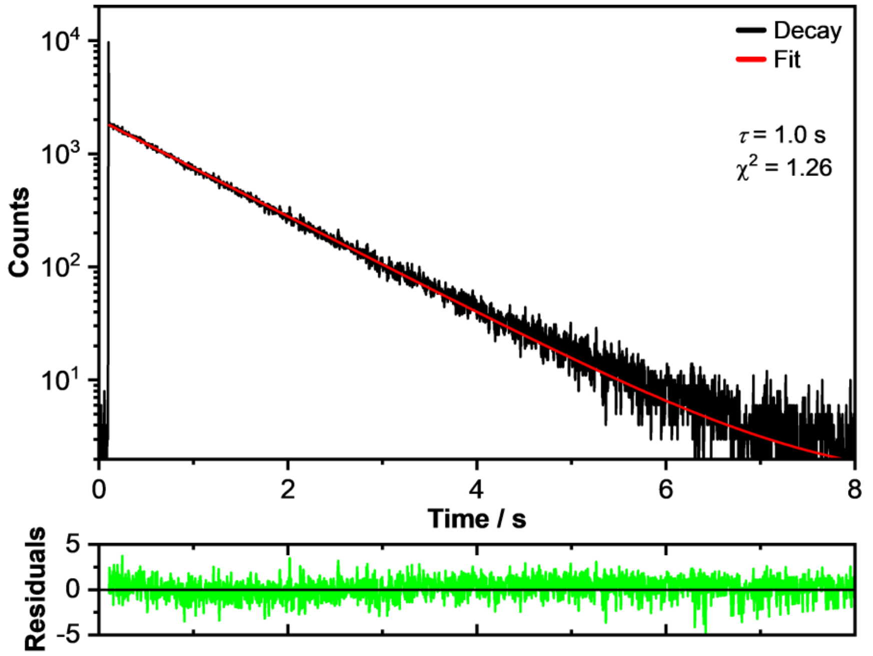

Figure S72. Phosphorescence lifetime decay and residual analysis (λ_{ex} = 424 nm, λ_{em} = 568 nm) of **1**$_{\mathbf{OBOBO}}$ (6.9×10^{-7} M) in 2-MeTHF at 77 K.

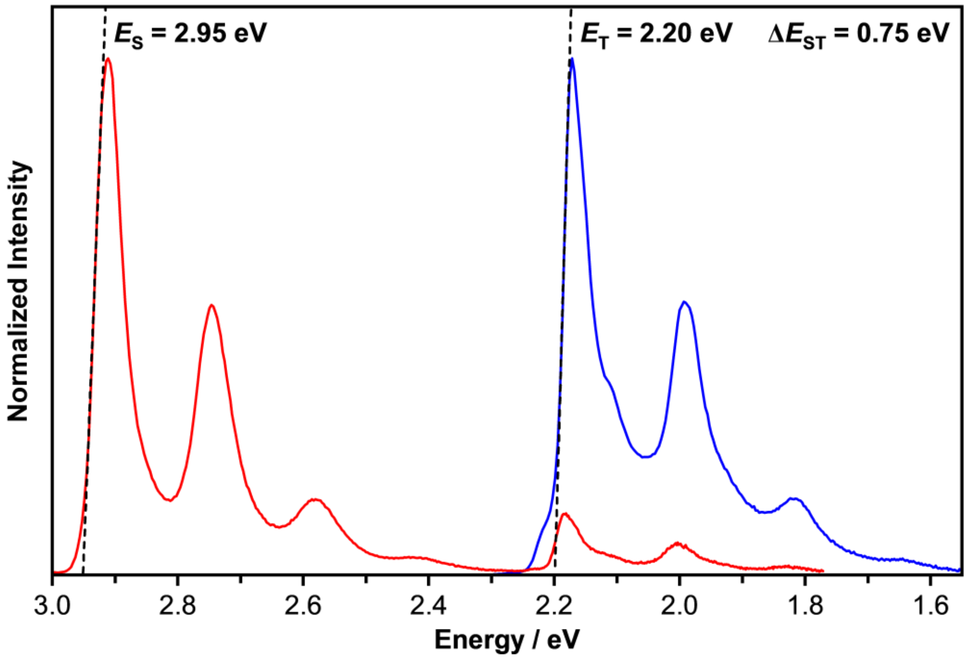

Figure S73. Determination of the singlet-triplet gap of $\mathbf{1}_{\mathbf{OBOBO}}$ (6.9×10^{-7} M) in 2-MeTHF at 77 K, using the onset of the stead-state fluorescence (red, λ_{ex} = 402 nm) and the phosphorescence (blue, λ_{ex} = 426 nm, gate delay: 1000 µs) emission spectra in 2-MeTHF at 77 K.

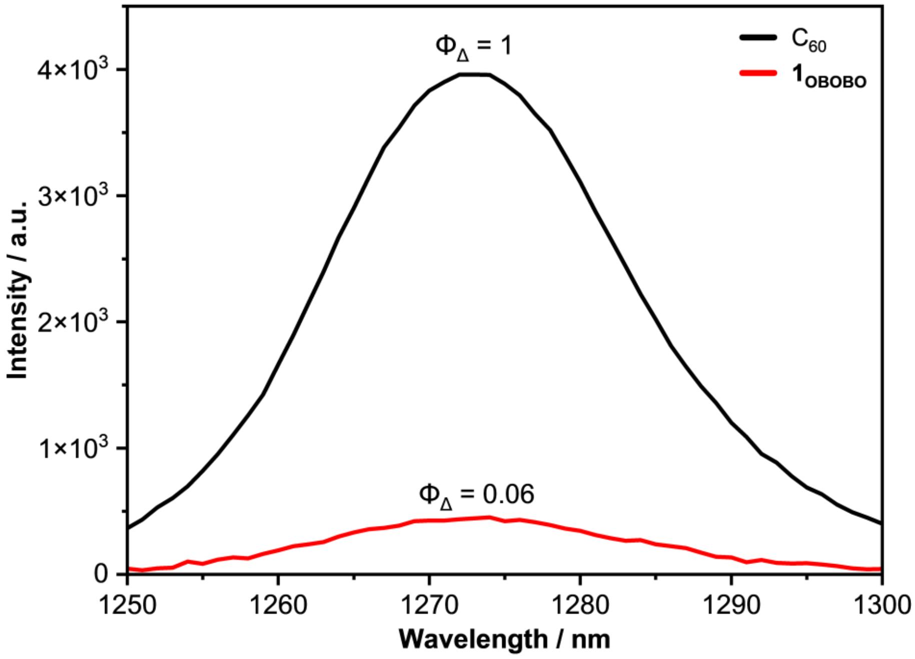

Figure S74. Singlet oxygen phosphorescence emission sensitized by C_{60} (black, 8.6×10^{-6} M, λ_{ex} = 375 nm) and $\mathbf{1}_{\mathbf{OBOBO}}$ (red, 7.1×10^{-7} M, λ_{ex} = 426 nm) in $CHCl_3$.

Molecule 2_{NBNBN}

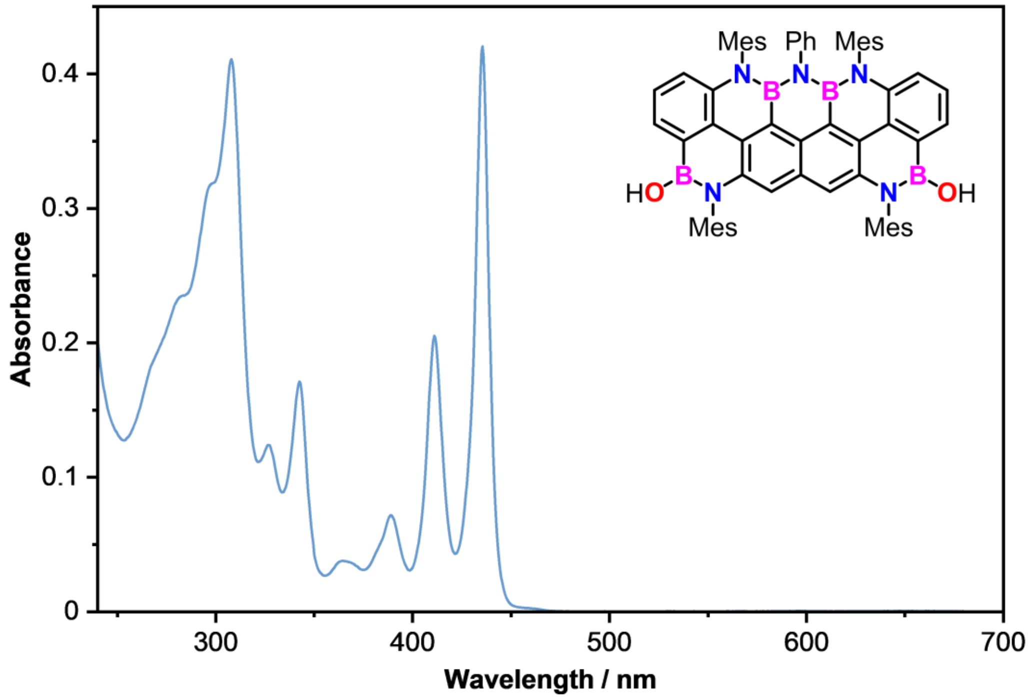

Figure S75. UV-vis absorption spectra of $\mathbf{2}_{NBNBN}$ (arb. conc.) in 2-MeTHF.

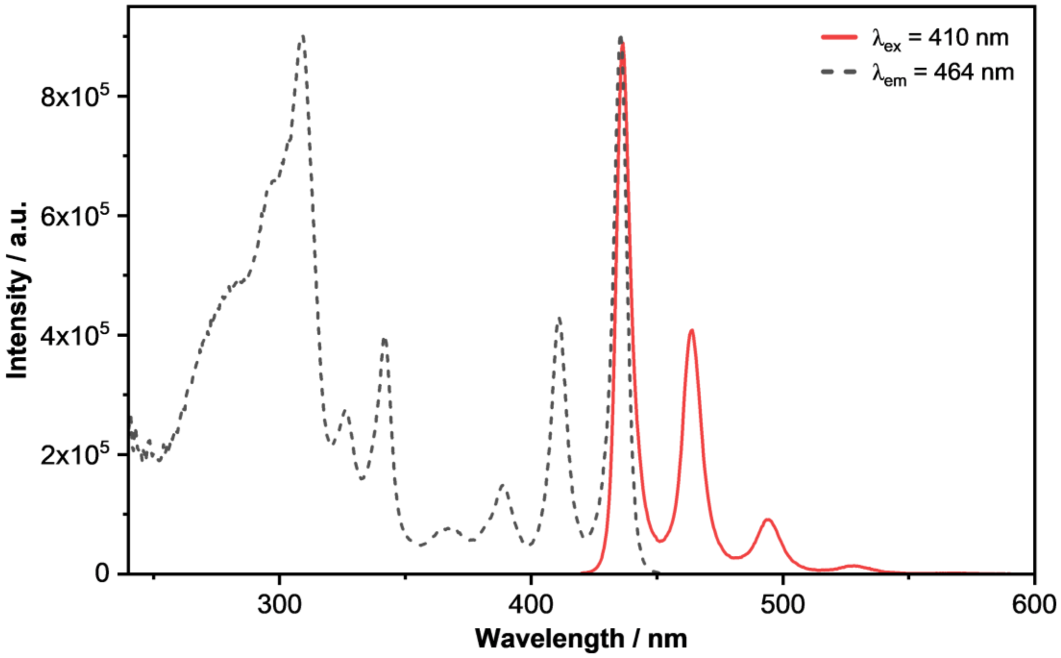

Figure S76. Steady-state excitation (dashed) and emission (solid) spectra of $\mathbf{2}_{NBNBN}$ (arb. conc.) in 2-MeTHF.

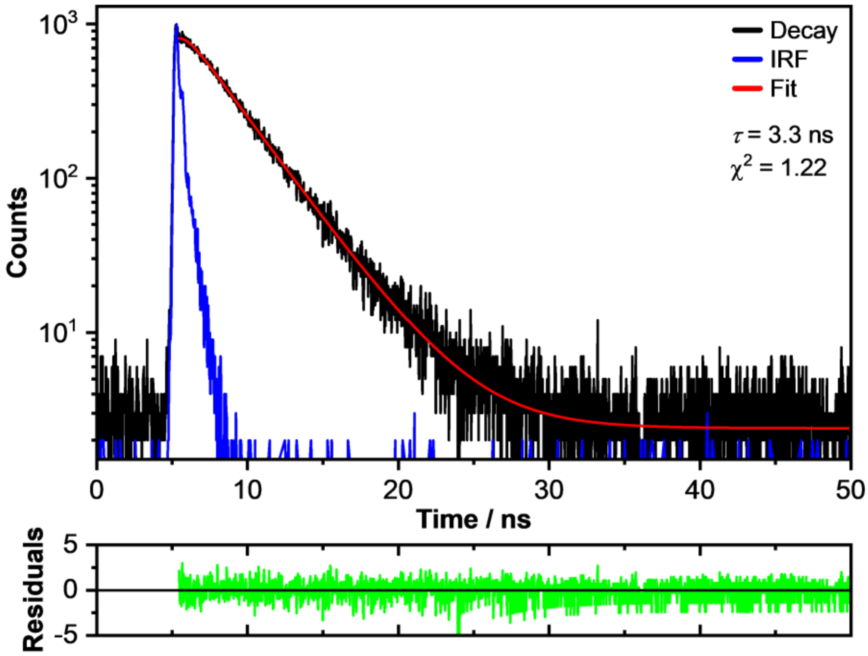

Figure S77. Fluorescence lifetime decay and residual analysis (λ_{ex} = 405.6 nm, λ_{em} = 440 nm) of $\mathbf{2_{NBNBN}}$ (arb. conc.) in 2-MeTHF.

Molecule $\mathbf{2_{NBOBN}}$

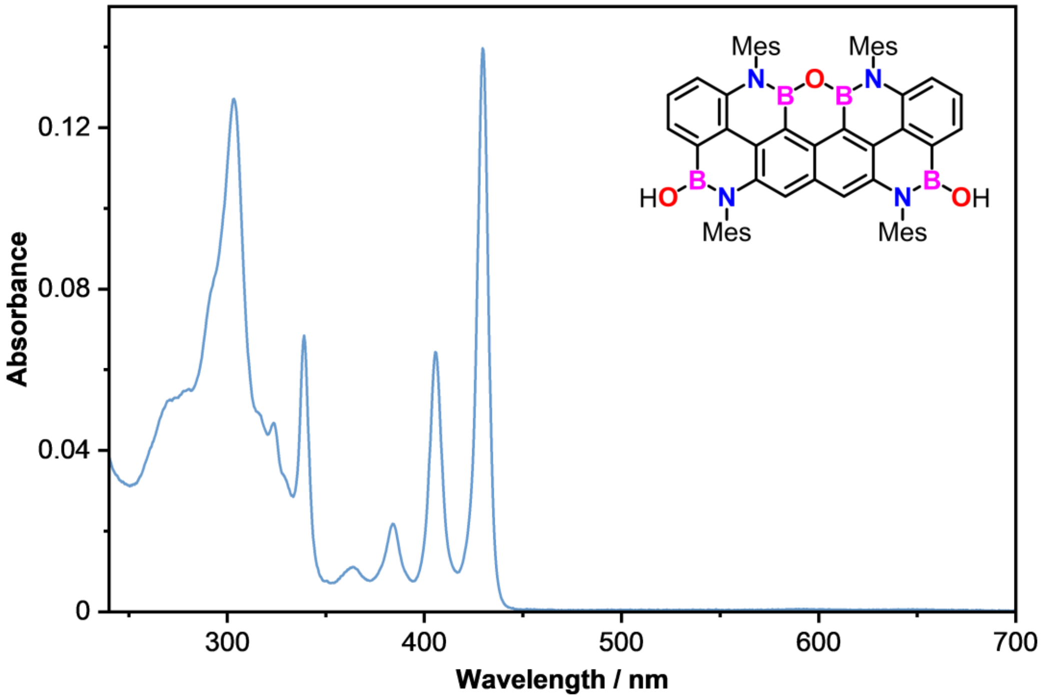

Figure S78. UV-vis absorption spectra of $\mathbf{2_{NBNBN}}$ (arb. conc.) in 2-MeTHF.

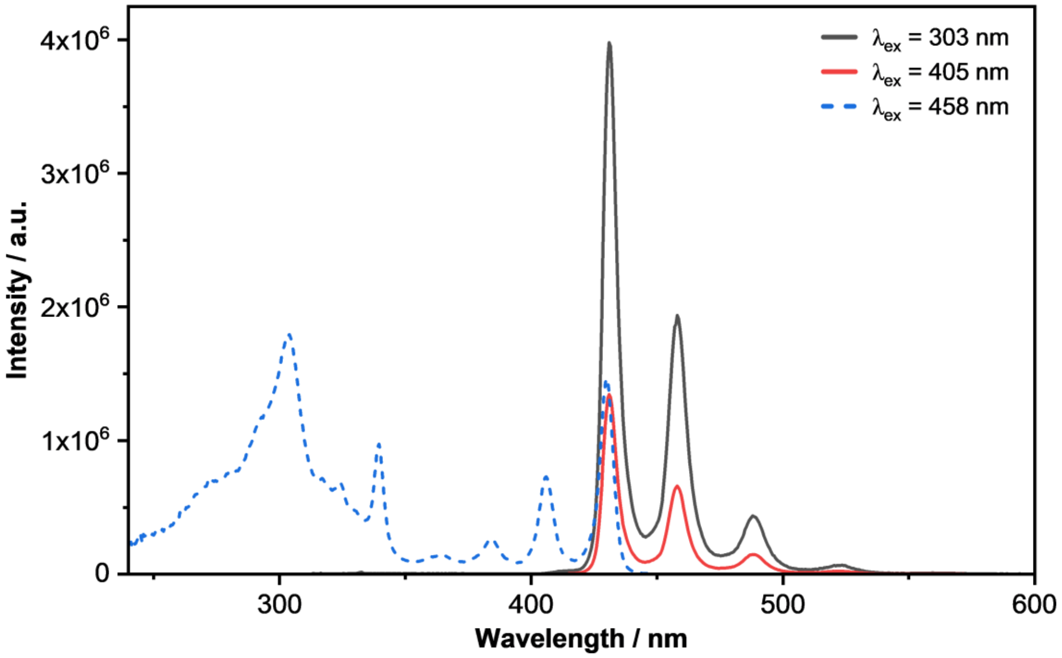

Figure S79. Steady-state excitation (dashed) and emission (solid) spectra of $\mathbf{2_{NBNBN}}$ (arb. conc.) in 2-MeTHF.

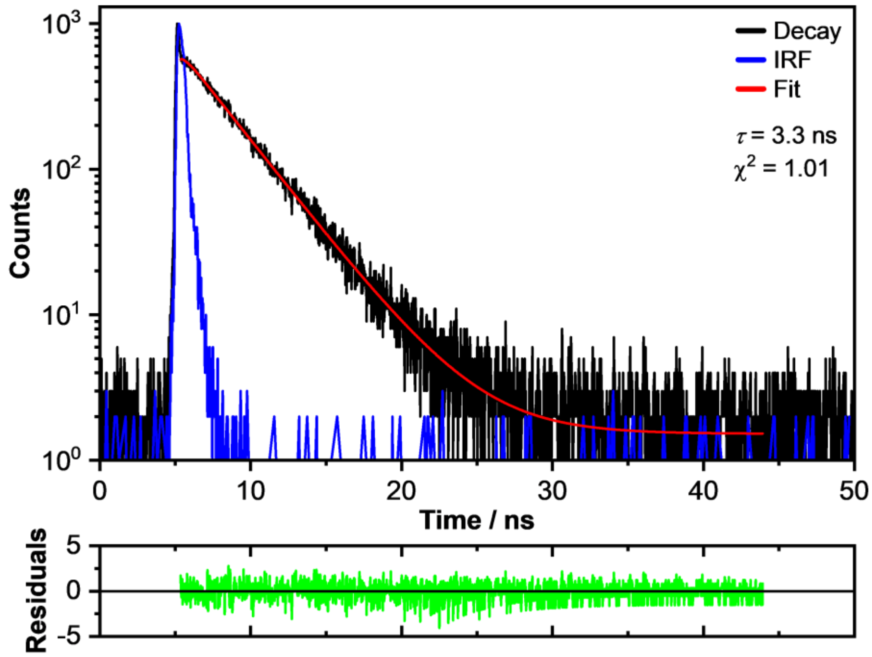

Figure S80. Fluorescence lifetime decay and residual analysis (λ_{ex} = 405.6 nm, λ_{em} = 434 nm) of $\mathbf{2_{NBNBN}}$ (arb. conc.) in 2-MeTHF.

Molecule 2_{OBNBO}

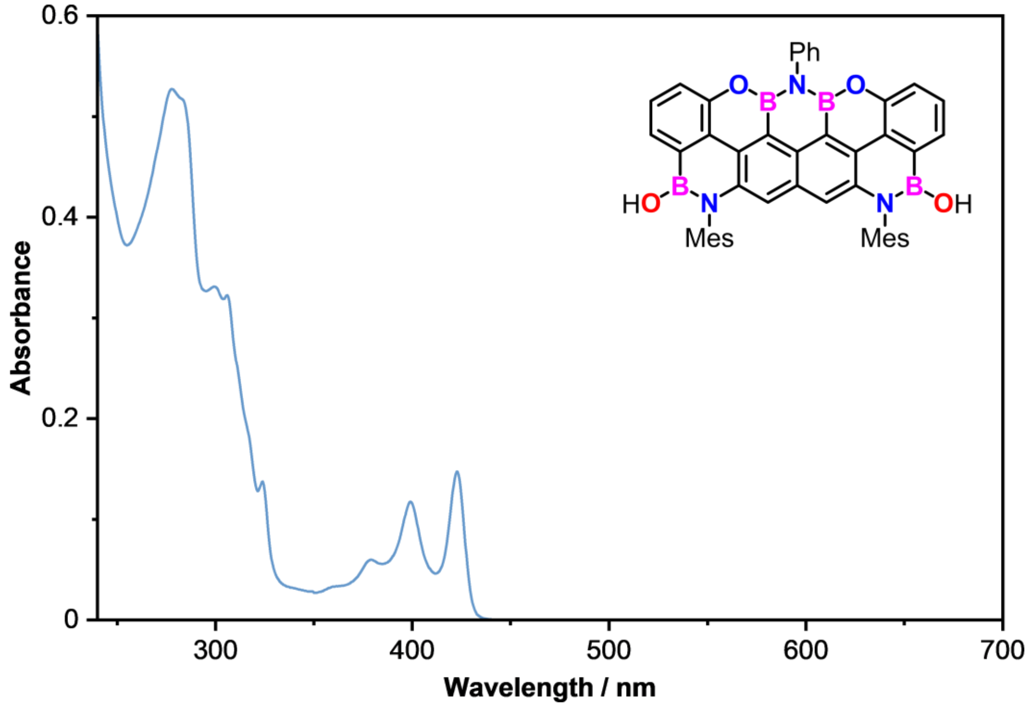

Figure S81. UV-vis absorption spectra of $\mathbf{2}_{\mathbf{OBNBO}}$ (arb. conc.) in 2-MeTHF.

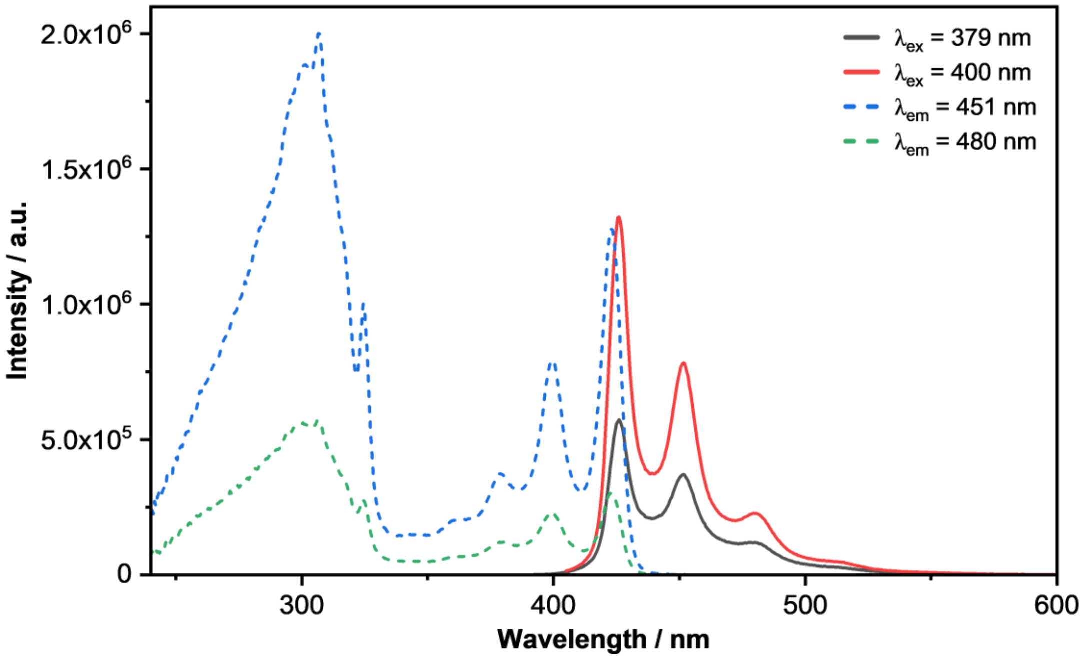

Figure S82. Steady-state excitation (dashed) and emission (solid) spectra of $\mathbf{2}_{\mathbf{OBNBO}}$ (arb. conc.) in 2-MeTHF.

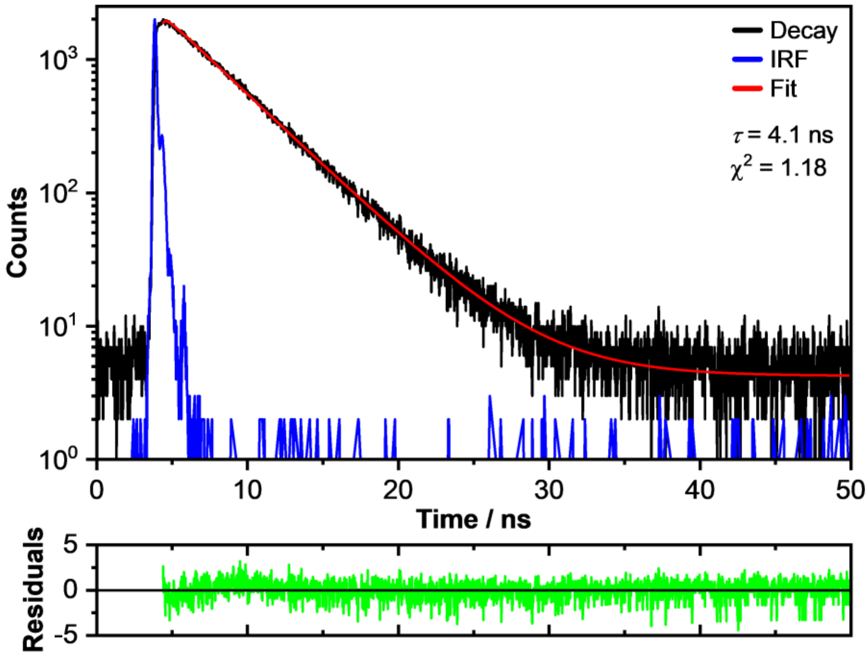

Figure S83. Fluorescence lifetime decay and residual analysis (λ_{ex} = 405.6 nm, λ_{em} = 426 nm) of $\mathbf{2_{OBNBO}}$ (arb. conc.) in 2-MeTHF.

Molecule $\mathbf{2_{OBOBO}}$

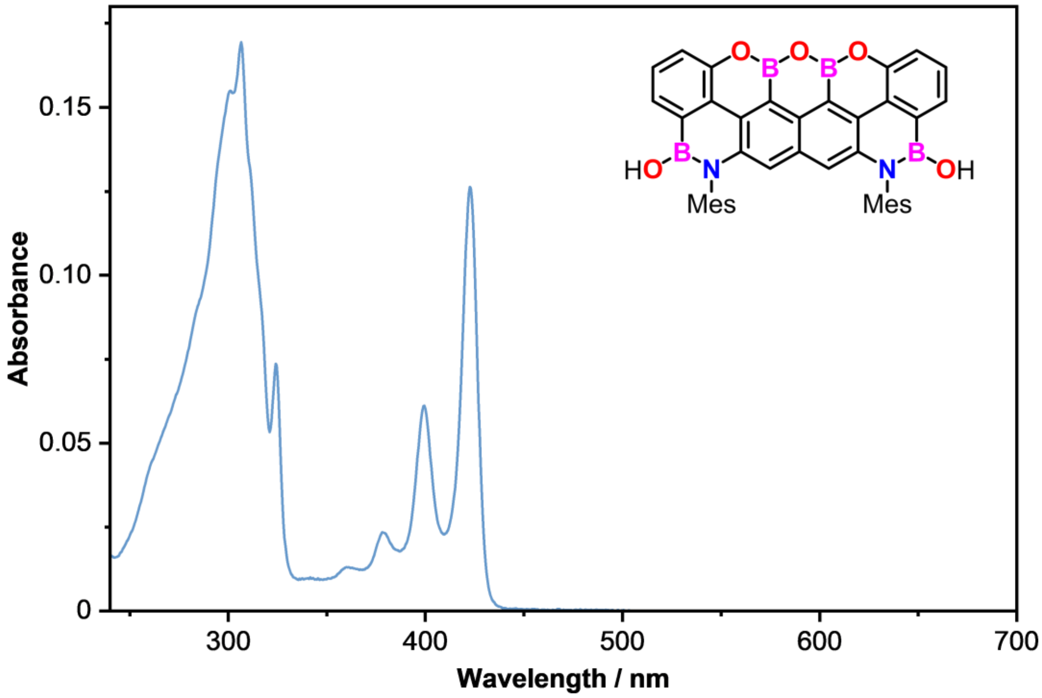

Figure S84. UV-vis absorption spectra of $\mathbf{2_{OBOBO}}$ (arb. conc.) in 2-MeTHF.

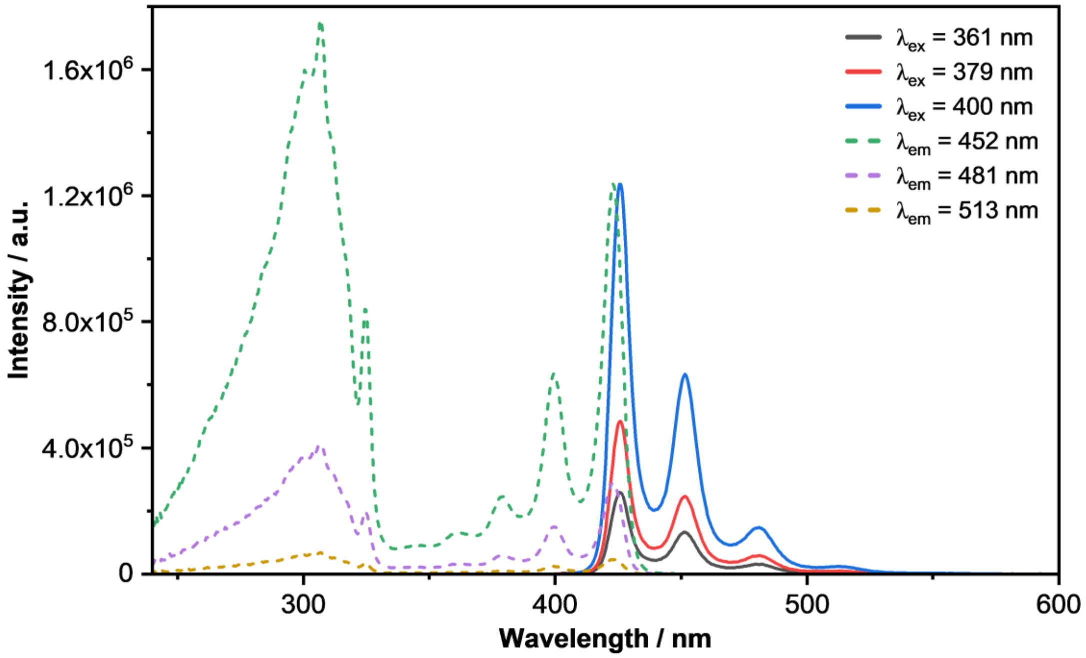

Figure S85. Steady-state excitation (dashed) and emission (solid) spectra of **2**$_{\mathbf{OBOBO}}$ (arb. conc.) in 2-MeTHF.

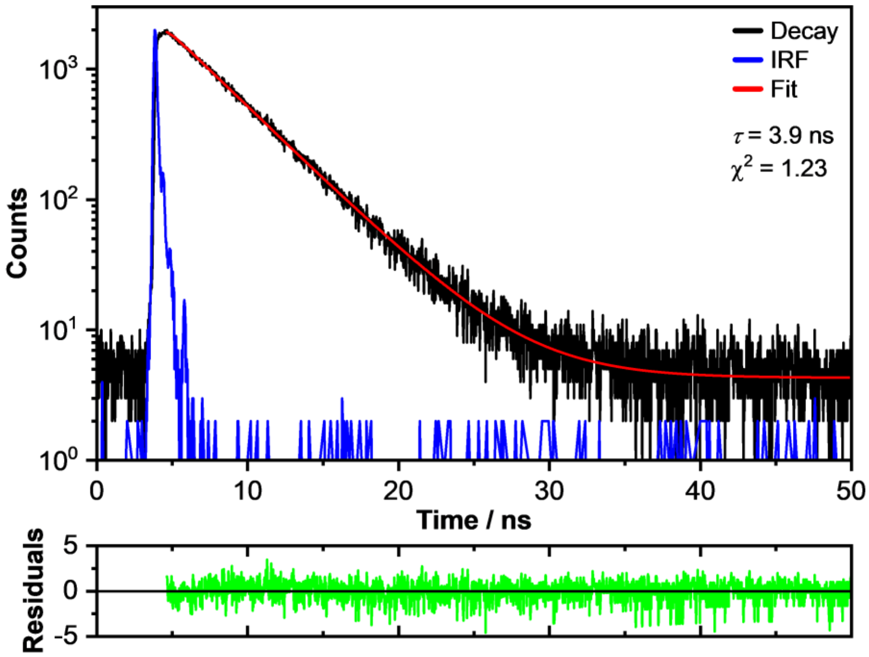

Figure S86. Fluorescence lifetime decay and residual analysis (λ_{ex} = 405.6 nm, λ_{em} = 426 nm) of **2**$_{\mathbf{OBOBO}}$ (arb. conc.) in 2-MeTHF.

Table S1. Summary of photophysical properties of **2y**.

	λ_{max} (nm)	λ_F (nm)	τ_F (ns)	E_{00}[a] (eV)
**2**$_{NBNBN}$	435	437	3.3	2.84
**2**$_{NBOBN}$	430	432	3.3	2.88
**2**$_{OBNBO}$	425	427	3.9	2.91
**2**$_{OBOBO}$	423	426	3.9	2.92

[a] Estimated from the crossing point between the absorbance and emission spectra.

6. Electrochemical characterization

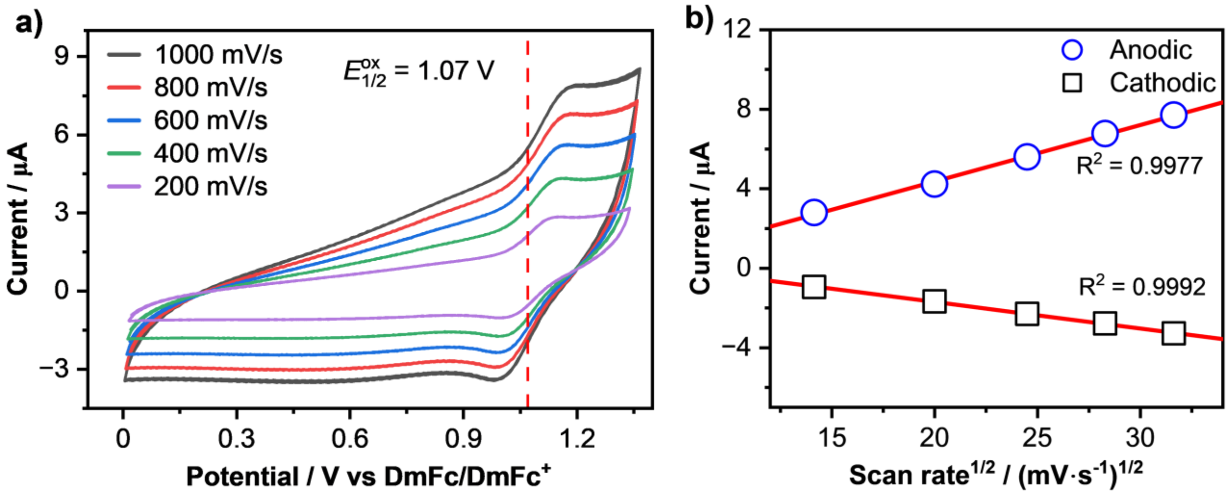

Figure S87. Electrochemical analysis of compound **1**$_{NBNBN}$ (0.2 mM) in TCE: a) Cyclic voltammogram at different scan rates (200 - 1000 mV/s); b) Linear dependence between peak current and scan rate$^{1/2}$.

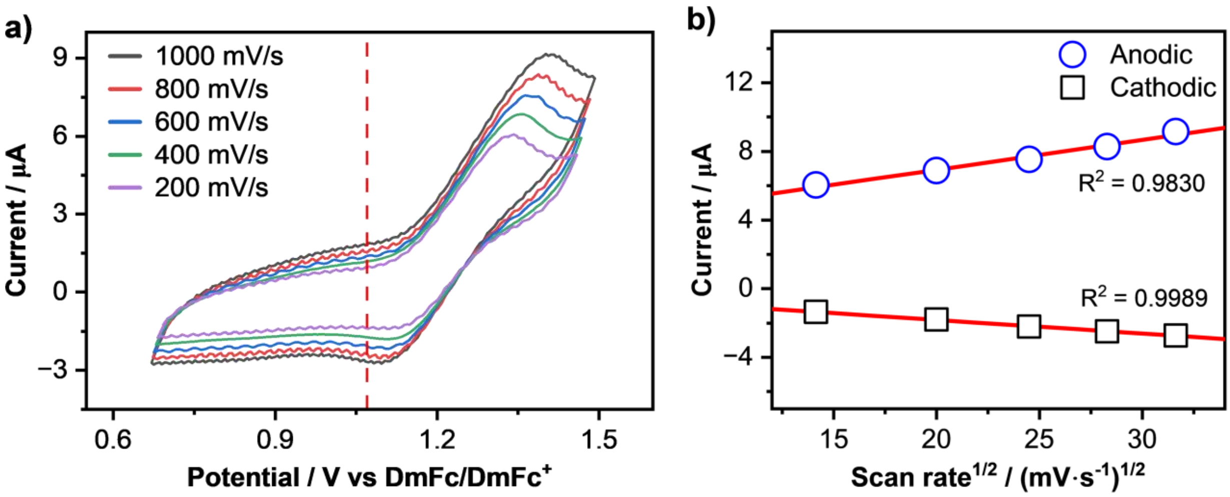

Figure S88. Electrochemical analysis of compound **1**$_{NBOBN}$ (0.2 mM) in TCE: a) Cyclic voltammogram at different scan rates (200 - 1000 mV/s); b) Linear dependence between peak current and scan rate$^{1/2}$.

7. Crystallographic data

Table S2. Crystal data and structure refinement for **1_{NBNBN}** (2279843).

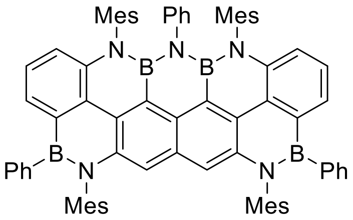

Empirical formula	$(C_{76}H_{67}B_4N_5)_2 \cdot C_7H_8$	
Formula weight	2279.30	
Crystal system	Triclinic	
Space group	*P*-1	
Unit cell dimensions	a = 13.040(3) Å	α = 94.40(3)°
	b = 13.320(3) Å	β = 100.00(3)°
	c = 19.710(4) Å	γ = 90.80(3)°
Volume	3360.2(12) Å^3	
Z	1	
Density (calculated)	1.126 mg/m^3	
Absorption coefficient	0.064 mm^{-1}	
F(000)	1206.0	
Crystal size	0.300 × 0.177 × 0.110 mm^3	
	**Data collection**	
Temperature	100 K	
Wavelength	0.71073 Å	
Theta range for data collection	2.919 to 26.022°	
Index ranges	-16 ≤ h ≤ 16, -15 ≤ k ≤ 16, -24 ≤ l ≤ 24	
Reflections collected	93252	
Independent reflections	13008 [R_{int} = 0.0835, R_{sigma} = 0.1851]	
Completeness to theta = 25.242°	98.4%	
	**Refinement**	
Absorption correction	multi-scan	
Refinement method	Full-matrix least-squares on F^2	
Data / restraints / parameters	13008 / 1491 / 812	
Goodness-of-fit on F^2	0.770	
Final R indices [I>2sigma(I)]	R_1 = 0.0483, wR_2 = 0.1061	
R indices (all data)	R_1 = 0.1121, wR_2 = 0.1126	
Largest diff. peak and hole	0.265 and -0.358 e·Å^{-3}	

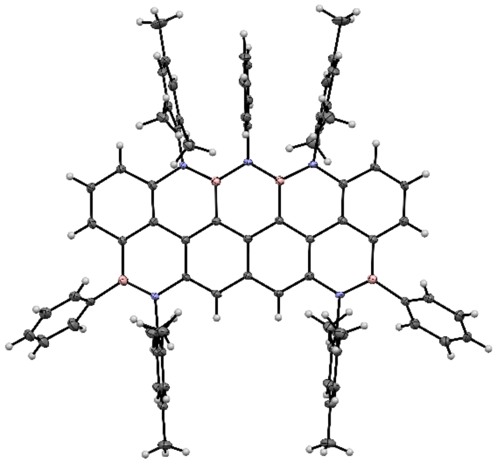

Figure S89. ORTEP representation (50% probability ellipsoids) of the X-ray crystal structure for **1_{NBNBN}**. Solvent molecule omitted for clarity. Atom colors: gray, C; pink, B; blue, N; red, O.

Table S3. Crystal data and structure refinement for **1_{NBOBN}** (2268752).

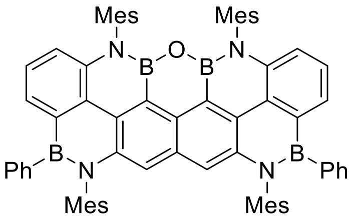

Empirical formula	$C_{70}H_{62}B_4N_4O\cdot C_7H_8$	
Formula weight	1110.61	
Crystal system	Triclinic	
Space group	*P*-1	
Unit cell dimensions	a = 12.156(2) Å	α = 70.80(3)°
	b = 22.540(5) Å	β = 87.70(3)°
	c = 23.900(5) Å	γ = 89.83(3)°
Volume	6179(2) $Å^3$	
Z	4	
Density (calculated)	1.194 mg/m^3	
Absorption coefficient	0.066 mm^{-1}	
F(000)	2352.0	
Crystal size	0.100 × 0.050 × 0.020 mm^3	
	**Data collection**	
Temperature	100 K	
Wavelength	0.700 Å (Synchrotron)	
Theta range for data collection	0.889 to 25.000°	
Index ranges	-14 ≤ h ≤ 14, -27 ≤ k ≤ 27, -28 ≤ l ≤ 28	
Reflections collected	22456	
Independent reflections	22456 [R_{sigma} = 0.0912]	
Completeness to theta = 24.835°	98.6%	
	**Refinement**	
Absorption correction	multi-scan	
Refinement method	Full-matrix least-squares on F^2	
Data / restraints / parameters	22456 / 192 / 1087	
Goodness-of-fit on F^2	1.023	
Final R indices [I>2sigma(I)]	R_1 = 0.1151, wR_2 = 0.3173	
R indices (all data)	R_1 = 0.1856, wR_2 = 0.3726	
Largest diff. peak and hole	0.89 and -0.62 $e\cdot Å^{-3}$	

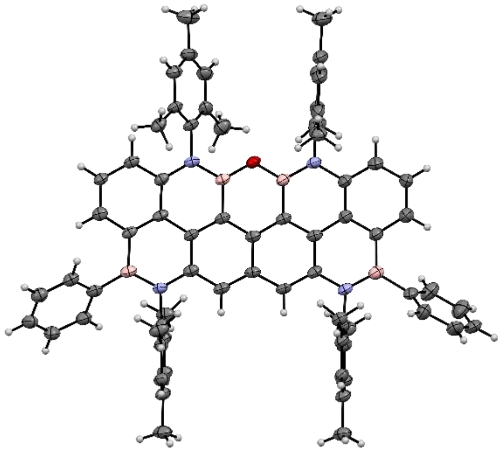

Figure S90. ORTEP representation (50% probability ellipsoids) of the X-ray crystal structure for **1_{NBOBN}**. A single, crystallographically independent, molecule representative of the crystal is shown. Solvent molecules omitted for clarity. Atom colors: gray, C; pink, B; blue, N; red, O.

Table S4. Crystal data and structure refinement for **1_{OBOBO}** (2331294).

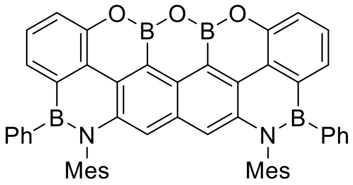

Empirical formula	$C_{52}H_{40}B_4N_2O_3 \cdot 3(C_7H_8)$	
Formula weight	1060.50	
Crystal system	Triclinic	
Space group	*P*-1	
Unit cell dimensions	a = 12.487(4) Å	α = 68.32(3)°
	b = 15.444(5) Å	β = 87.12(3)°
	c = 16.365(6) Å	γ = 75.37(2)°
Volume	2834.2(17) Å^3	
Z	2	
Density (calculated)	1.243 mg/m^3	
Absorption coefficient	0.074 mm^{-1}	
F(000)	1120.0	
Crystal size	0.580 × 0.237 × 0.020 mm^3	
	**Data collection**	
Temperature	100 K	
Wavelength	0.71073 Å	
Theta range for data collection	3.179 to 25.027°	
Index ranges	-12 ≤ h ≤ 14, -18 ≤ k ≤ 17, -19 ≤ l ≤ 19	
Reflections collected	50016	
Independent reflections	9756 [R_{int} = 0.1282, R_{sigma} = 0.3335]	
Completeness to theta = 25.027°	97.4%	
	**Refinement**	
Absorption correction	multi-scan	
Refinement method	Full-matrix least-squares on F^2	
Data / restraints / parameters	9756 / 1206 / 732	
Goodness-of-fit on F^2	0.831	
Final R indices [I>2sigma(I)]	R_1 = 0.0684, wR_2 = 0.1447	
R indices (all data)	R_1 = 0.2203, wR_2 = 0.1696	
Largest diff. peak and hole	0.33 and -0.30 e·Å^{-3}	

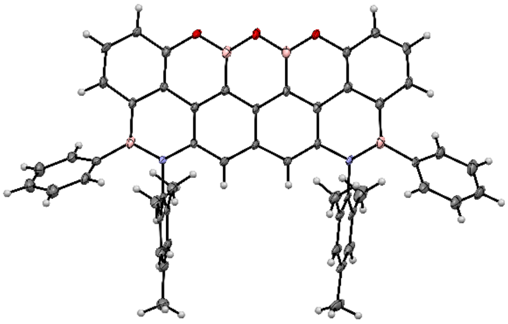

Figure S91. ORTEP representation (50% probability ellipsoids) of the X-ray crystal structure for **1_{OBOBO}**. Solvent molecules omitted for clarity. Atom colors: gray, C; pink, B; blue, N; red, O.

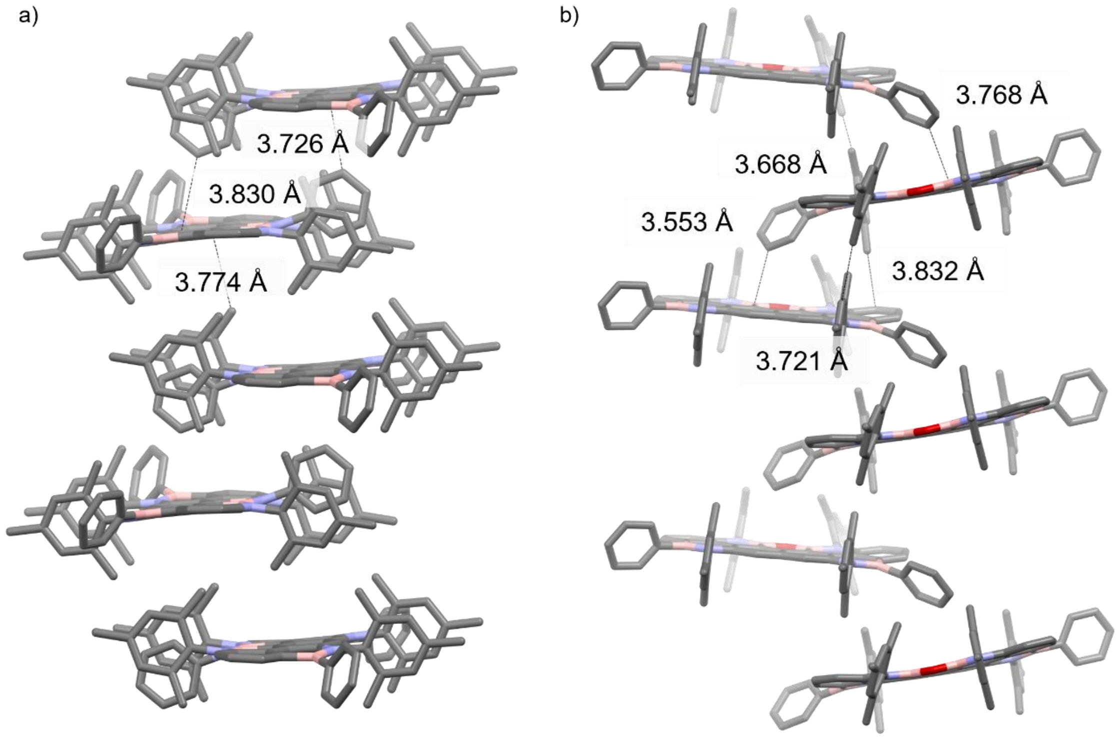

Figure S92. Solid-state organization of (a) **1_{NBNBN}** and (b) **1_{NBOBN}** developing through C-H…π interactions (expressed in Å). Hydrogen atoms and solvent molecules are omitted for clarity. Atom colors: gray, C; pink, B; blue, N; red, O.

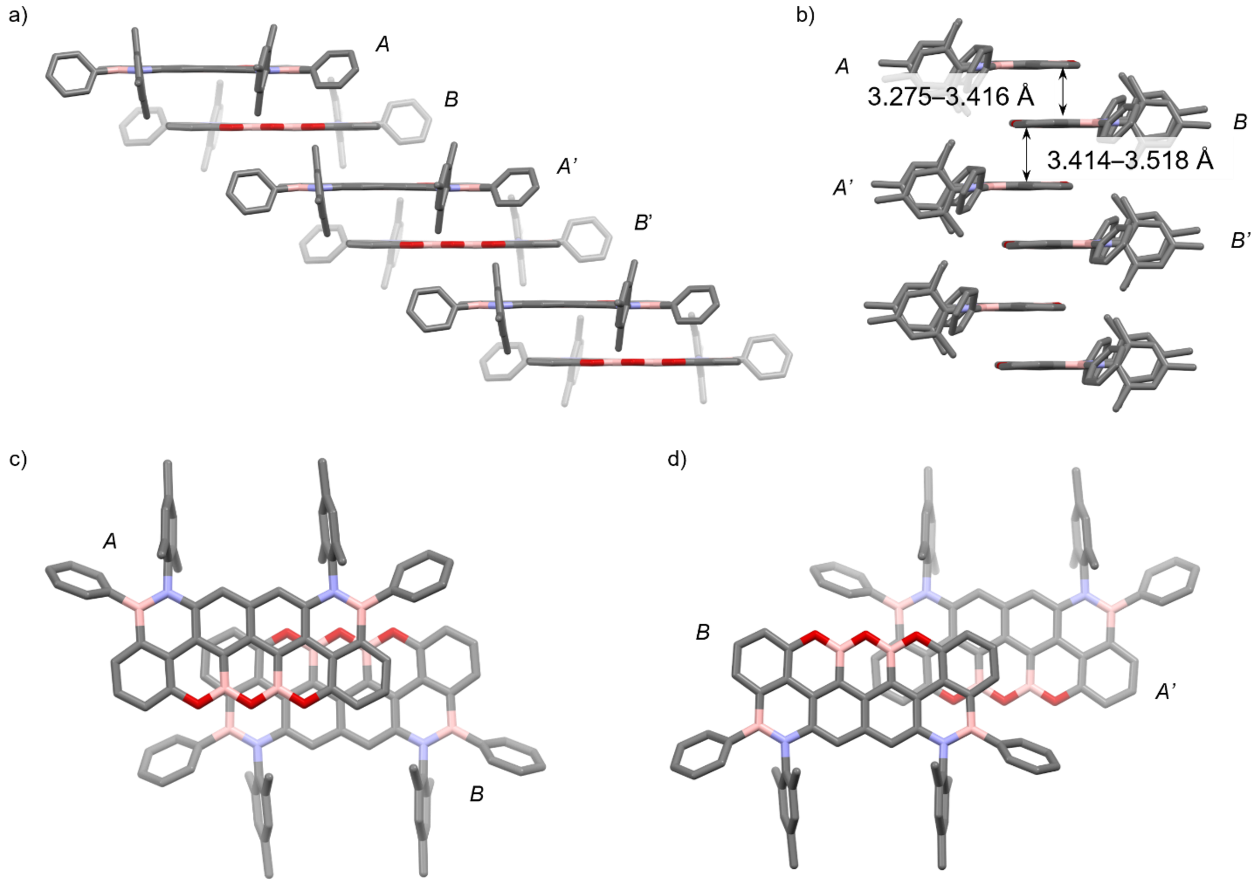

Figure S93. Solid-state columnar π-π stacks of **1**$_{OBOBO}$ (a,b) with interplanar spacings expressed in Å; (c,d) *anti* offset π-π stacking molecular arrangements (4.191 Å and 6.739 Å, respectively). Hydrogen atoms and solvent molecules are omitted for clarity. Atom colors: gray, C; pink, B; blue, N; red, O.

8. Computational studies

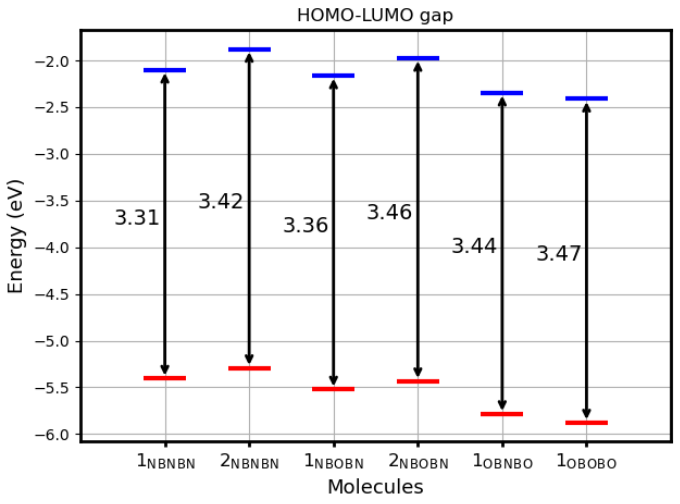

Figure S94. HOMO-LUMO energy level diagram of compounds **1**$_{NBNBN}$, **2**$_{NBNBN}$, **1**$_{NBOBN}$, **2**$_{NBOBN}$, **1**$_{OBNBO}$, and **1**$_{OBOBO}$, computed using DFT. The unsynthesized compound **1**$_{OBNBO}$ is included for comparison.

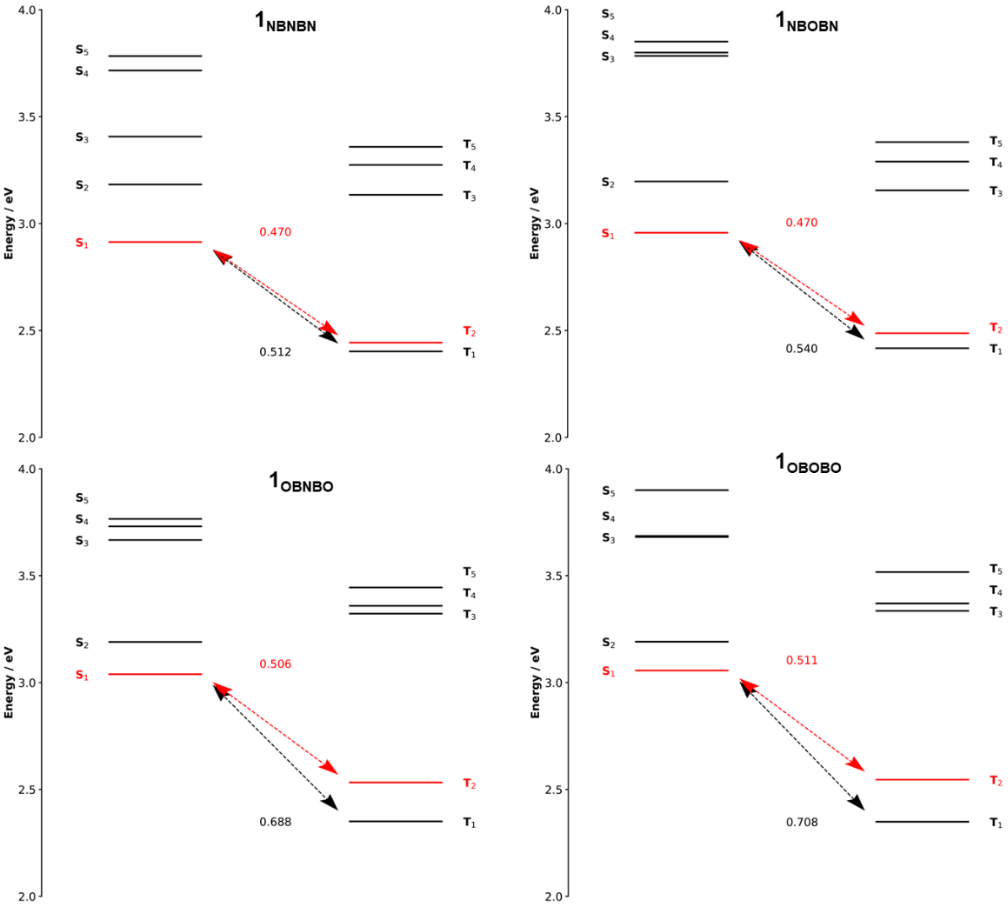

Figure S95. Energy level diagrams of the lowest singlet (S_1–S_5) and triplet (T_1–T_5) excited states for compounds **1**$_{NBNBN}$, **1**$_{NBOBN}$, **1**$_{OBNBO}$, and **1**$_{OBOBO}$, computed using TDA-DFT. The diagrams highlight the energetic proximity between the S_1 and T_2 states, with red and black arrows indicating the $S_1 \rightarrow T_2$ and $T_1 \rightarrow S_1$ energy gaps, respectively. The values next to the arrows correspond to the energy differences (in eV), emphasizing trends in intersystem crossing accessibility across the series. The unsynthesized compound **1**$_{OBNBO}$ is included as a reference to complete the comparison of N→O substitution effects on excited-state energies.

9. References

[1] A. Lausi, M. Polentarutti, S. Onesti, J. R. Plaisier, E. Busetto, G. Bais, L. Barba, A. Cassetta, G. Campi, D. Lamba, A. Pifferi, S. C. Mande, D. D. Sarma, S. M. Sharma, G. Paolucci, *Eur. Phys. J. Plus* **2015**, *130*, 43.

[2] W. Kabsch, *Acta Crystallogr., Sect. D* **2010**, *66*, 133–144.

[3] O. V. Dolomanov, L. J. Bourhis, R. J. Gildea, J. A. K. Howard, H. Puschmann, *J. Appl. Crystallogr.* **2009**, *42*, 339–341.

[4] G. Sheldrick, *Acta Crystallogr., Sect. A* **2015**, *71*, 3–8.

[5] G. Sheldrick, *Acta Crystallogr., Sect. C* **2015**, *71*, 3–8.

[6] J. Koziskova, F. Hahn, J. Richter, J. Kožíšek, *Acta Chim. Slovaca* **2016**, *9*, 136–140.

[7] C. B. Hubschle, G. M. Sheldrick, B. Dittrich, *J. Appl. Crystallogr.* **2011**, *44*, 1281–1284.

[8] C. Lee, W. Yang, R. G. Parr, *Phys. Rev. B* **1988**, *37*, 785–789.

[9] A. D. Becke, *J. Chem. Phys.* **1993**, *98*, 5648–5652.

[10] R. Krishnan, J. S. Binkley, R. Seeger, J. A. Pople, *J. Chem. Phys.* **1980**, *72*, 650–654.

[11] S. Grimme, S. Ehrlich, L. Goerigk, *J. Comput. Chem.* **2011**, *32*, 1456–1465.

[12] V. Barone, M. Cossi, *J. Phys. Chem. A* **1998**, *102*, 1995–2001.

[13] M. Cossi, N. Rega, G. Scalmani, V. Barone, *J. Comput. Chem.* **2003**, *24*, 669–681.

[14] M. J. Frisch, G. W. Trucks, H. B. Schlegel, G. E. Scuseria, M. A. Robb, J. R. Cheeseman, G. Scalmani, V. Barone, G. A. Petersson, H. Nakatsuji, X. Li, M. Caricato, A. V. Marenich, J. Bloino, B. G. Janesko, R. Gomperts, B. Mennucci, H. P. Hratchian, J. V. Ortiz, A. F. Izmaylov, J. L. Sonnenberg, Williams, F. Ding, F. Lipparini, F. Egidi, J. Goings, B. Peng, A. Petrone, T. Henderson, D. Ranasinghe, V. G. Zakrzewski, J. Gao, N. Rega, G. Zheng, W. Liang, M. Hada, M. Ehara, K. Toyota, R. Fukuda, J. Hasegawa, M. Ishida, T. Nakajima, Y. Honda, O. Kitao, H. Nakai, T. Vreven, K. Throssell, J. A. Montgomery Jr., J. E. Peralta, F. Ogliaro, M. J. Bearpark, J. J. Heyd, E. N. Brothers, K. N. Kudin, V. N. Staroverov, T. A. Keith, R. Kobayashi, J. Normand, K. Raghavachari, A. P. Rendell, J. C. Burant, S. S. Iyengar, J. Tomasi, M. Cossi, J. M. Millam, M. Klene, C. Adamo, R. Cammi, J. W. Ochterski, R. L. Martin, K. Morokuma, O. Farkas, J. B. Foresman, D. J. Fox, Wallingford, CT, **2016**.

[15] A. L. Fetter, J. D. Walecka, *Quantum Theory Of Many Particle Systems*, McGraw-Hill, New York, **1971**.

[16] S. Hirata, M. Head-Gordon, *Chem. Phys. Lett.* **1999**, *314*, 291–299.

[17] F. Santoro, A. Lami, R. Improta, J. Bloino, V. Barone, *J. Chem. Phys.* **2008**, *128*.

[18] V. Barone, J. Bloino, M. Biczysko, F. Santoro, *J. Chem. Theory Comput.* **2009**, *5*, 540–554.

[19] T. Lu, F. Chen, *J. Comput. Chem.* **2012**, *33*, 580–592.

[20] H. A. Staab, K. Elbl-Weiser, C. Krieger, *Eur. J. Org. Chem.* **2000**, *2000*, 327–333.
